# A structural remedy toward bright dipolar fluorophores in aqueous media†
†Electronic supplementary information (ESI) available: Materials and procedures regarding the synthesis of all the dyes and the biothiol probes, one-photon and two-photon spectroscopic analysis, tissue and cell imaging experiments, and theoretical computations. See DOI: 10.1039/c5sc01076d


**DOI:** 10.1039/c5sc01076d

**Published:** 2015-05-18

**Authors:** Subhankar Singha, Dokyoung Kim, Basab Roy, Sunderraman Sambasivan, Hyunsoo Moon, Alla Sreenivasa Rao, Jin Yong Kim, Taiha Joo, Jae Woo Park, Young Min Rhee, Taejun Wang, Ki Hean Kim, Youn Ho Shin, Junyang Jung, Kyo Han Ahn

**Affiliations:** a Department of Chemistry , Pohang University of Science and Technology (POSTECH) , 77 Cheongam-Ro, Nam-Gu , Pohang , Gyungbuk , Republic of Korea 790-784 . Email: ahn@postech.ac.kr; b Division of Integrative Biosciences and Bio-technology , Pohang University of Science and Technology (POSTECH) , 77 Cheongam-Ro, Nam-Gu , Pohang , Gyungbuk , Republic of Korea 790-784; c Department of Anatomy and Neurobiology , School of Medicine , Biomedical Science Institute , Kyung Hee University , 26 Kyungheedae-Ro, Dongdaemun-Gu , Seoul , Republic of Korea 130-701

## Abstract

The donor–acceptor (D–A) type dipolar fluorophores, an important class of luminescent dyes with two-photon absorption behaviour, generally emit strongly in organic solvents but poorly in aqueous media. To understand and enhance the poor emission behaviour of dipolar dyes in aqueous media, we undertake a rational approach that includes a systematic structure variation of the donor, amino substituent of acedan, an important two-photon dye. We identify several factors that influence the emission behaviour of the dipolar dyes in aqueous media through computational and photophysical studies on new acedan derivatives. As a result, we can make acedan dyes emit bright fluorescence under one- and two-photon excitation in aqueous media by suppressing the liable factors for poor emission: 1,3-allylic strain, rotational freedom, and hydrogen bonding with water. We also validate that these findings can be generally extended to other dipolar fluorophores, as demonstrated for naphthalimide, coumarin and (4-nitro-2,1,3-benzoxadiazol-7-yl)amine (NBD) dyes. The new acedan and naphthalimide dyes thus allow us to obtain much brighter two-photon fluorescent images in cells and tissues than in their conventional forms. As an application of these findings, a thiol probe is synthesized based on a new naphthalimide dye, which shows greatly enhanced fluorescence from the widely used *N*,*N*-dimethyl analogue. The results disclosed here provide essential guidelines for the development of efficient dipolar dyes and fluorescence probes for studying biological systems, particularly by two-photon microscopy.

## Abstract 2

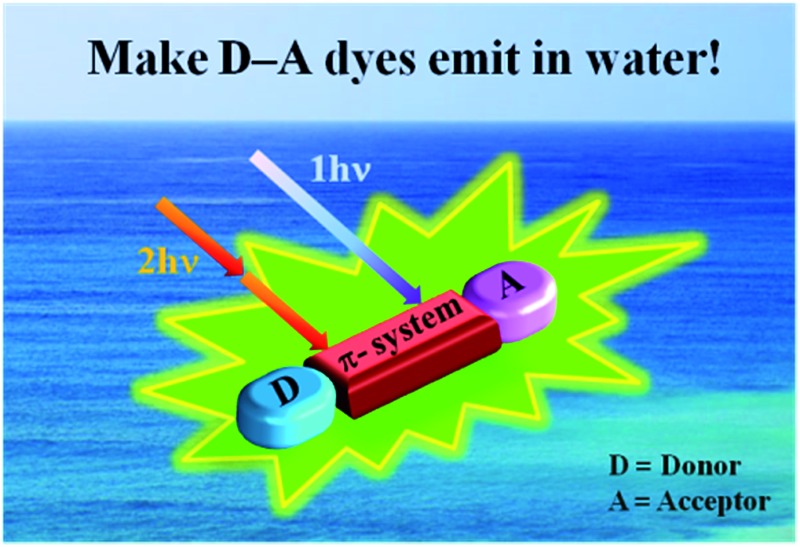
Structural factors governing the poor emission of dipolar dyes in aqueous media are identified, leading to new acedan derivatives with brighter fluorescence and enhanced two-photon properties.

## Introduction

Donor–acceptor (D–A) type dipolar fluorophores have been widely used in molecular probes and biological tags owing to their highly emissive nature.^1^,^2^ Representative classes of dipolar dyes include acedan (2-acetyl-6-(dimethylamino)naphthalene), naphthalimide (4-amino-1,8-naphthalimide), coumarin (7-aminocoumarin), benzocoumarin, NBD ((4-nitro-2,1,3-benzoxadiazol-7-yl)amine), dansyl (5-amino-naphthalene-1-sulfonyl), rhodol, Nile red and blue, and so on. These dyes used so far mostly contained a dialkylamino group as the electron-donor group and an electron-withdrawing moiety (acetyl, nitro, cyanide, dicyanovinyl, *etc.*), both conjugated to an aromatic system in such a way that the donor and acceptor are electronically conjugated. Such dipolar dyes generate intramolecular charge transfer (ICT) excited states upon irradiation with light, which bestows the dipolar dyes with environment-sensitive photophysical properties: typically, they emit at longer wavelengths as the polarity of the medium increases. This environment-sensitive property of the dipolar fluorophores has been importantly utilized in the development of polarity-sensitive molecular probes.^2^ On the other hand, they emit weakly in polar and poorly in aqueous media, in contrast to the “symmetric” dyes that have symmetric resonance structures, such as BODIPY, rhodamine, cyanine, and fluorescein derivatives, which generally show medium polarity-insensitive emission properties. In spite of numerous applications of dipolar fluorophores in areas of chemistry, biology, and medical sciences, the factors that make them emit poorly in aqueous media have not been fully understood. Considering that these dipolar dyes have been used in various types of molecular probes and tags for biological analytes, a better understanding of the factors that influence their emission properties in water is of fundamental importance. Furthermore, if we could improve their poor emission properties in water, the results would hold great promise for the development of molecular probes, in particular, two-photon imaging probes for biological systems as dipolar dyes constitute an important class of two-photon excitable fluorophores.^3^

Among the dipolar dyes, acedan and its analogues constitute an important class of two-photon excitable fluorophores for bioimaging of tissues, as they are small in size, can be readily modified, and show good two-photon absorption properties.^4^ Two-photon (absorbing) dyes that can be excited in the near-infrared wavelength region have received great interest as they enable deeper tissue imaging with reduced autofluorescence as well as with very high spatial and temporal resolution by two-photon microscopy (TPM).^5^ In spite of the promising two-photon properties of acedan and its derivatives, and other various dipolar fluorophores in general, their poor emission behaviour in aqueous media has not been fully addressed yet.^6^

Our interest in the development of two-photon fluorescent probes based on acedan7a,b and benzocoumarins7*c* brought about questions on the factors that influence the emission intensity of dipolar fluorophores in aqueous media. Herein, we disclose a rational approach to identify those factors, which has led to new acedan derivatives with significant enhancement in their fluorescence, particularly two-photon properties, in aqueous media. Accordingly, it was possible to achieve very bright and sharp two-photon images in cells/tissues with the new acedan derivatives. We also validate that the same approach can be generally extended to other important D–A type fluorophores such as naphthalimide, coumarin and NBD dyes, thereby greatly expanding the applicability of such dipolar dyes in search of molecular probes and imaging agents for biological systems.

## Results and discussion

**Fig. 1:**
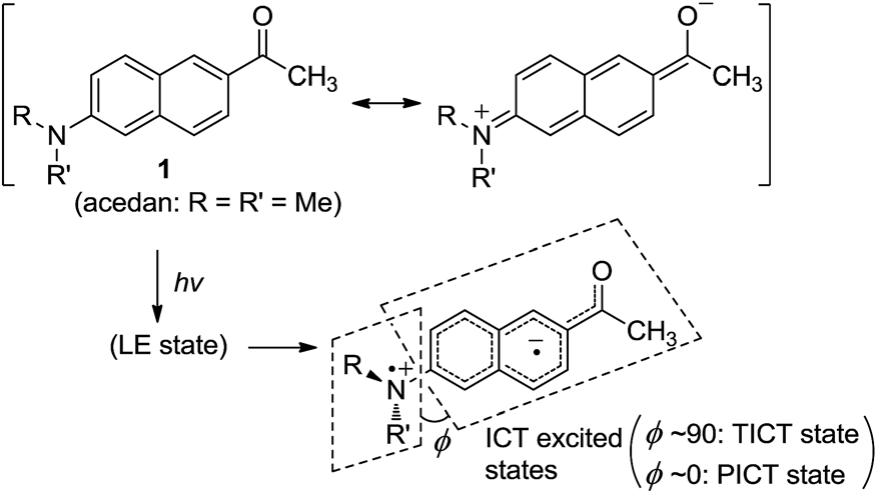
Acedan (**1**) and its ICT excited states, where *φ* is the torsional angle between the naphthalene and the R–N–R′ planes when viewed along the C_naphthyl_–N bond.

Acedan has an electron-donating 6-dimethylamino group and an electron-accepting 2-acetyl group, allowing ICT through the π-electron delocalization (Fig. 1). Similar to a typical D–A type dye (*e.g.*, 4-dimethylaminobenzonitrile),^8^ emission of acedan and its N-substituted derivatives is expected to come from the locally excited (LE) state, or the ICT excited states—twisted (TICT) or planarized (PICT), depending on the medium and the N-substituents.^9^

### Our rationale

**Fig. 2:**
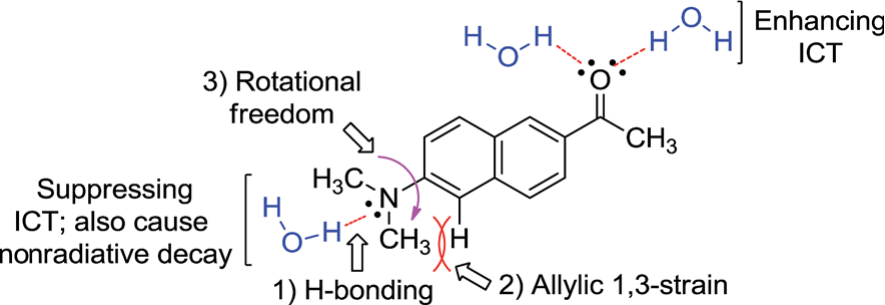
The proposed factors in this study that would affect ICT in the dipolar fluorophores with an amino donor group: acedan as an example.

In general, for a given D–A type fluorophore, reducing the donor or acceptor ability causes a decrease in the emission intensity. In other words, any factors that suppress the ICT character in acedan or in other dipolar fluorophores would lead to a reduction in the transition dipole moment and hence a reduction in the fluorescence intensity.^8^ The dipolar fluorophores emit strongly in organic media generally but poorly in water, from which we presume that hydrogen (H)-bonding between the dye and water molecules offers the major nonradiative deactivation pathway, in addition to the highly polar nature of water. Hence, we supposed that H-bonding to the amine nitrogen by water molecules would lower the fluorescence emission (Fig. 2). Our approach to suppress the nitrogen H-bonding is to introduce a hydrophobic N-substituent in acedan, which may obstruct the accessibility of water molecules to the nitrogen (for supporting results by theoretical calculations, see ESI†). Suppressing the nitrogen H-bonding would also exclude an initial solvent reorganization process at the nitrogen lone pair, a required process for attaining the ICT excited state.^10^

The second factor effecting the ICT process (PICT or TICT) is the substitution pattern at the donor nitrogen, which determines the electron-donating ability and the 1,3-allylic strain (Fig. 2). *N*,*N*-Dialkylamino groups are better in terms of the electron donating ability compared to the corresponding monoalkyl derivatives; instead, a larger 1,3-allylic strain^11^ is expected in the former case. Initial quantum chemical calculations show that the *N*-methylamino analogue has a transition moment (1.57 D) larger than that of acedan (1.45 D), an *N*,*N*-dimethylamino derivative, indicative of the importance of the allylic strain. Thus, the N-substitution pattern affects the excitation and hence the emission intensity.^12^,^13^

The third factor that can affect the fluorescence intensity of acedan derivatives is the rotational freedom of the amino group (Fig. 2). A bulky amino group is expected to undergo a slower or hindered internal rotation in the excited state, which can also contribute to the fluorescence enhancement of acedan derivatives by reducing the nonradiative decay.

**Fig. 3:**
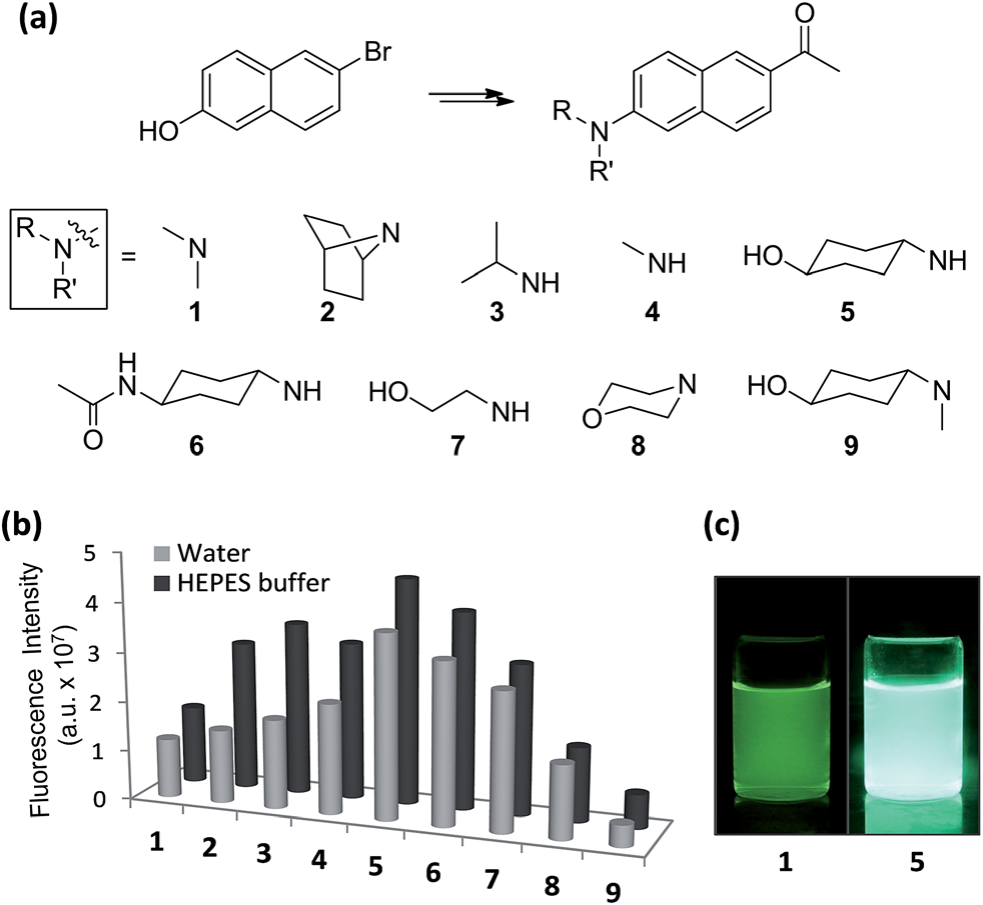
One-photon emission properties of acedan derivatives. (a) Structures of acedan (**1**) and its derivatives **2–9** investigated in this study. (b) Comparison of one-photon fluorescence intensity of acedan (**1**) and its derivatives **2–9** (each at 10 μM) in aqueous media; the fluorescence was measured by excitation at the maximum absorbance wavelength (*λ*_abs_) of each compound. The uncertainty is less than ±10% for all the measurements. (c) Photos of acedan (**1**) and **5** in water (10 μM) under UV irradiation (365 nm).

With the aforementioned rationales in mind and confirming their computational results (details in ESI†), we have prepared various *N*-alkyl- and *N*,*N*-dialkyl-substituted acedan derivatives with different steric bulkiness, and compared their photophysical properties. A series of compounds **2–9** including acedan (Fig. 3a) are thus synthesized by adopting our improved method of introducing the acetyl group,^14^ and their photophysical properties are thoroughly evaluated.^15^

### Photophysical properties of acedan and its derivatives

Absorption spectra for acedan (**1**) and its derivatives **2–9** were obtained in solvents with a wide polarity span (HEPES buffer, water,^16^ ethanol, acetonitrile, *N*,*N*-dimethylformamide, dichloromethane, and cyclohexane) (Fig. S1, ESI†). We confirmed that all the compounds are soluble in the concentration range evaluated. A comparison of the absorbance for compounds **1–9** in different solvents (Tables S1 and S2, ESI†) shows that acedan derivatives with *N*-cyclohexyl groups (**5** and **6**) have increased absorbance, particularly in water; compound **5** shows more than 1.5-fold enhanced molar absorptivity (*ε*) from that of acedan (Table 1).

**Table 1:** Spectroscopic data for acedan (**1**) and its derivatives **2–9** in water

Compd	*λ*_abs_ ^*a*^ (nm)	*ε* ^*b*^ (M^–1^ cm^–1^)	*λ*_em_ ^*c*^ (nm)	*Φ*_F_ ^*d*^	Brightness (*ε* × *Φ*_F_)	*τ* ^*e*^ (ns)
*1*	*362*	*11* *200*	*515*	*0.20*	*2240*	*1.29*
2	338	14 600	515	0.30	4350	n.d.^*f*^
3	357	14 500	501	0.31	4495	n.d.^*f*^
4	355	13 500	501	0.32	4320	n.d.^*f*^
*5*	*357*	*17* *400*	*503*	*0.40*	*6960*	*3.15*
6	357	15 400	503	0.39	6006	3.05
7	351	11 000	495	0.31	3410	3.40
8	332	12 600	515	0.23	2898	n.d.^*f*^
9	368	9100	517	0.09	819	n.d.^*f*^

^*a*^The maximum absorbance wavelength.

^*b*^Molar extinction coefficient.

^*c*^The maximum emission wavelength.

^*d*^Fluorescence quantum yield, measured by one-photon excitation at 370 nm.

^*e*^Fluorescence life time measured at the center region of the emission spectra.

^*f*^n.d. = Not determined. The uncertainty is less than ±10% for all the measurement.

Emission spectra for compounds **1–9** in various solvents show a significant variation in the intensity, particularly in aqueous media (Fig. S2, ESI†). All the compounds emit more strongly in the organic solvents than in water as usual, except for nonpolar cyclohexane. A comparison of the emission intensity for compounds **1–9** in aqueous media shows significant improvement for compounds **5** and **6** over acedan (Fig. 3b). Clearly *N*-monoalkyl derivatives (**3–7**) emit enhanced fluorescence from those of *N*,*N*-dialkyl derivatives (**1**, **2**, **3** and **9**), which confirms that the N-substitution pattern significantly influences the formation of the ICT states and their nonradiative decay processes.^8^ Although further study is required to discuss the emissive state, acedan derivatives with an *N*-monoalkyl donor seem to attain a PICT state that emits strongly, rather than a TICT state that emits poorly.^8^,^17^ *N*-Cyclohexanol analogue **5** give the largest enhancement (threefold) among the acedan derivatives, emitting much brighter fluorescence in water compared with that of acedan when excited at 365 nm (Fig. 3c). Its *N*-(4-acetamido)cyclohexyl analogue **6** also shows a similar but slightly lower enhancement. On the other hand, compound **9** shows very weak fluorescence in water, probably through a TICT excited state caused by a strong allylic strain. Then, we compared quantum yields of compounds **1–9** measured in different solvents (Tables 1 and S5, ESI†). The high quantum yields of acedan in organic solvents (*Φ*_F_ = 0.48 in CH_2_Cl_2_; 0.52 in CH_3_CN) decrease in water (*Φ*_F_ = 0.20). In contrast, compound **5** still shows a comparable quantum yield (*Φ*_F_ = 0.40) in water to those observed with acedan in organic solvents. A similar level of quantum yields is observed in the case of *N*-acetyl analogue **6**. Other *N*-monoalkyl derivatives (**3**, **4** and **7**) show values between those of acedan and **5**. In fact, with enhanced absorbance and quantum yield, the brightness (*ε* × *Φ*_F_) for compound **5** improved more than three times from that of acedan in water (Table 1).

We measured the picosecond time-resolved photoluminescence for acedan and its derivatives **5–7** at the blue, center and red regions of the emission spectra in different solvents by using the time-correlated single photon counting (TCSPC) technique (Fig. S7–S9, ESI†). The average fluorescence lifetimes (*τ*) calculated from the spectra (Tables S7–S9, ESI†) show that acedan has a much shorter lifetime in water (1.05–1.49 ns) than in other solvents (2.99–3.19 ns in CH_3_CN; 3.04–3.46 ns in CH_2_Cl_2_), whereas the acedan derivatives **5–7** have longer lifetimes in water (2.75–3.69 ns) similar to those in the organic solvents (2.51–3.09 ns in CH_3_CN; 2.51–3.19 ns in CH_2_Cl_2_). These results also support that the ICT state of acedan undergoes a rapid nonradiative decay in water, but such a process is retarded in the case of the acedan derivatives **5–7**.

At this point, it is necessary to comment on the possible emissive states of acedan and its derivatives. It is a still controversial issue whether acedan has the PICT or TICT as the emissive state.^9^ Recently it was proposed that a molecule whose conformation in the ground state is close to that of the ICT excited state may undergo ultrafast ICT, whereas a molecule whose conformation is distinct from that of the ICT excited state may undergo a slower ICT process *via* a locally excited (LE) state, which involves the twisting motion of the amino group.^18^ Also, the potential energy surface of the ground state along the twisting coordinate of the amino group is rather shallow to allow a distribution of the torsional angle (*φ*) in the ground state.^8^ From our molecular dynamics (MD) simulations results, compound **5** shows a nearly flat conformation (*φ* = 1.66°) plausibly due to the reduced allylic strain, whereas the calculated ground state conformation of acedan shows a small torsional angle (*φ* = 10.47°). Upon excitation, compound **5** thus seems to attain a PICT state readily; whereas acedan may initially generate a partially-twisted ICT state, which is further twisted to form a fully-twisted ICT state (a TICT state) that can be stabilized in water to give poor emission. Again, the PICT state would become more emissive as the amino donor group experiences a restricted rotational freedom by favourable structural factors (as in the cases of **5** and **6**). Thus, the allylic strain and the rotational freedom associated with the donor amino group govern the emission behaviour of the dipolar dyes in water. Additionally, the H-bonding to the donor amino group by water affects the ICT at both the ground and excited states, and also provides the solvent-mediated relaxation route for the excited state, thereby affecting the emission behaviour of dipolar dyes in water. Although it is difficult to separate the effect of one factor from others and also the emissive sate is under debate, still the allylic strain seems to play the major role based on a rough analysis of our experimental results (see ESI† for more detailed analysis).

The dipolar dyes generally emit strongly in most organic solvents through the ICT excited states. Accordingly, compound **9**, which emits poorly in water, fluoresces strongly in CH_2_Cl_2_ and CH_3_CN. The H-bonding-mediated relaxation of the excited state is absent in organic media, which leads to strong fluorescence in general. As we pointed out above, compound **9** seems to emit *via* a partially-twisted ICT state in organic solvent, rather than through the fully twisted charge-separated ICT state (the TICT state) that is stabilized in the highly polar water. In organic solvent, the emission intensity of dipolar dyes seems to be dependent mostly on the degree of the ICT; a more electron-donating amino group would cause more ICT and thus cause the corresponding dye to emit more strongly.

**Fig. 4:**
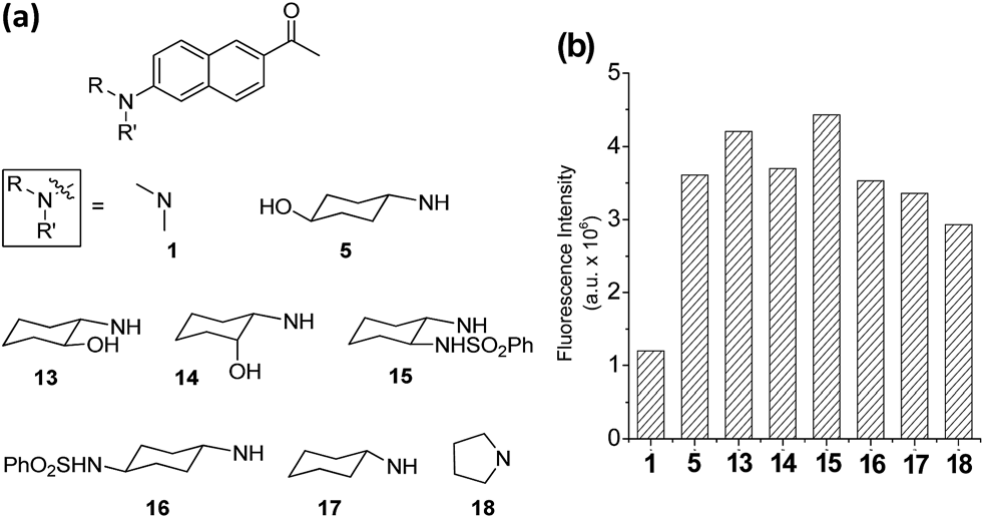
Acedan derivatives and their relative emission intensity in water. (a) Acedan derivatives **13–18** investigated further. (b) Comparison of one-photon fluorescence intensities of acedan (**1**) and its derivatives **5**, **13–18** (each at 1 μM) in aqueous medium; the fluorescence intensity was measured by excitation at the maximum absorbance wavelength (*λ*_abs_) of each compound. The uncertainty is less than ±10% for all the measurements.

On the basis of the structural effects on the emission behaviour of acedan derivatives in water, we have further synthesized several derivatives of dye **5** and compared their emission behaviour; these compounds have an additional substituent on the cyclohexyl ring, either at nearby the amino group (compounds **13–15**) or at the remote site (compound **16**) (Fig. 4a). Also, their parent cyclohexylamine analogue (compound **17**) was compared with a pyrrolidine analogue **18**. Dyes with a pyrrolidine as the donor are known to emit strongly.^19^ Compounds **13** and **15** have an additional substituent near the donor amine which causes a small but further increase in the emission intensity from that of compound **5** (Fig. 4b and S10, ESI†), indicative of positive substituent effects: a reduction in the rotational freedom or additional blocking of the water hydrogen bonding to the donor nitrogen, or both. Finally, it should be noted that the pyrrolidine analogue **18** shows slightly lower but comparable fluorescence to that of compound **17**, although they are poorly soluble in water which limited further evaluation. The enhanced fluorescence of compound **18** from that of acedan (**1**) can be rationalized by evoking the reduced allylic strain caused by the five-membered pyrrolidine ring, which, on the other hand, would enhance the ICT. Indeed, compound **18** shows the absorption and emission maxima red-shifted by 22 nm and 8 nm (Table S10, ESI†), respectively, from those of acedan. Although dyes with pyrrolidine^19^ and azetidine6*e* donors also show a comparable level of emission to those dyes with monoalkylamino donors, the latter type of donor has an advantageous feature over the former type: it can be further functionalized to develop fluorescent probes, in particular activatable probes through a carbamate linker.4c,4d

### Two-photon absorbing properties of acedan derivatives

**Fig. 5:**
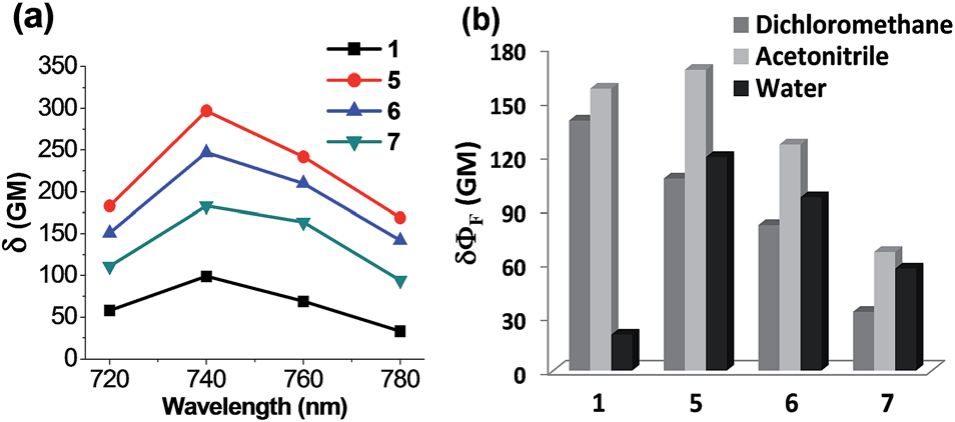
Two-photon photophysical properties of acedan derivatives. (a) Plots of two-photon absorption cross-sections (*δ*) of acedan (**1**) and its derivatives **5–7** in water. The values were measured with rhodamine B as a standard. The uncertainty is less than ±10%. (b) Comparison of two-photon action cross-section (*δΦ*_F_) values obtained for acedan (**1**) and its derivatives **5–7** in different solvents (dichloromethane, acetonitrile and water) under excitation at 740 nm (the maximum two-photon excitation wavelength). The uncertainty is less than ±10% for all the measurements.

Of particular note is that the substituent effects on the emission behaviour of acedans are more pronounced under the two-photon excitation conditions. As mentioned previously, acedan and its derivatives have been recognized as an important class of two-photon dyes owing to their structural simplicity and good two-photon absorption properties.^3^ We have compared the two-photon absorption cross-sections (TPACS, *δ*) for selected compounds in different solvents and by changing the excitation wavelength (Fig. 5 and S11, ESI†). The TPACS value of *N*-cyclohexyl derivative **5** (297 GM) is three times higher than that of acedan in water (99 GM) when obtained under excitation at 740 nm (Fig. 5a and Table S11, ESI†). In the case of analogue **6**, a slightly lower TPACS value than that of **5** was observed (247 GM). With the enhanced quantum yields, both compounds **5** and **6** thus show greatly enhanced two-photon action cross-section (*δΦ*_F_) values from those of acedan: 6- and 5-fold-enhanced values in water, respectively (Fig. 5b). As a result, the new acedan derivative **5** is expected to show bright fluorescence that is not very sensitive to the environment as in the case of acedan, an important but hitherto unexplored feature from such dipolar fluorophores. The conventional environment-sensitive D–A type fluorophores can yield undesired artifacts from the environmental changes that they would experience in different biological compartments. In this context, our approach provides a solution to this issue. Again, compound **7**, a (monoalkyl)amine derivative, shows two-photon properties better than those of acedan in water, but inferior to those of **5** and **6**.

### Extension to other dipolar fluorophores

**Fig. 6:**
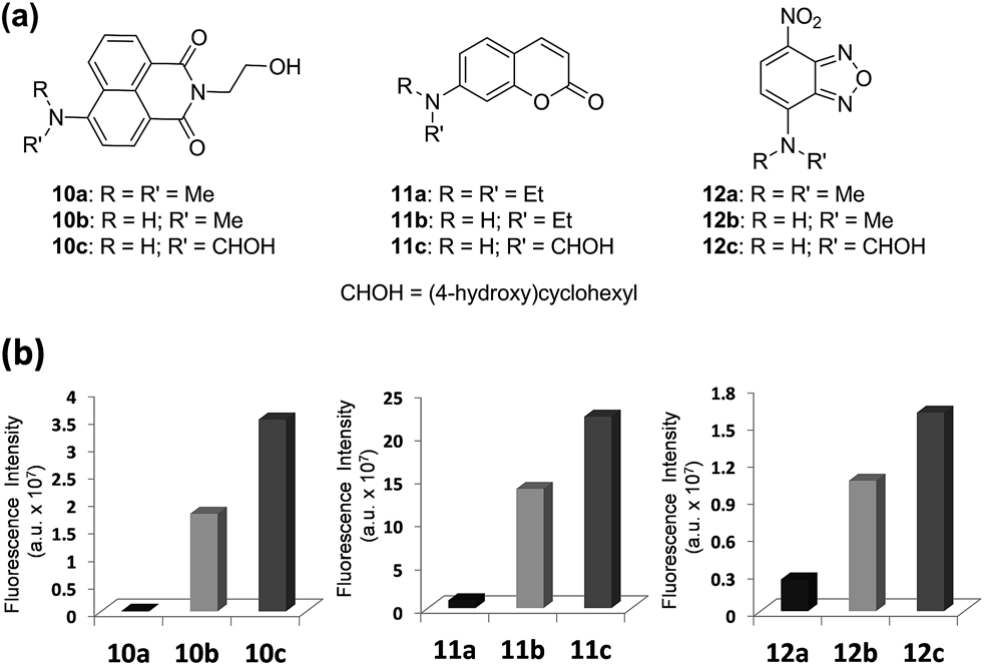
Comparison of emission intensities depending on the amino substituent of three common dipolar fluorophores. (a) Structures of naphthalimides **10a–10c**, coumarins **11a–11c** and NBDs **12a–12c**. (b) Comparison of fluorescence intensities of naphthalimides **10a–10c**, coumarins **11a–11c** and NBDs **12a–12c** (each at 10 μM) in water; the fluorescence intensity was measured by excitation at the maximum absorbance wavelength (*λ*_abs_) of each compound. The uncertainty is less than ±10% for all the measurements.

Next, we validate that the above approach can be generally extended to other dipolar fluorophores. For this purpose, three well-known classes of dyes are selected: 4-amino-1,8-naphthalimide, 7-aminocoumarin and NBD. The corresponding *N*,*N*-dialkylamino (**10a**, **11a**, and **12a**), *N*-monoalkylamino (**10b**, **11b**, and **12b**) and *N*-cyclohexylamino (**10c**, **11c**, and **12c**) derivatives were synthesized and their emission intensities were compared (Fig. 6 and S4–S6, ESI†). We have confirmed that naphthalimide **10a**, a model compound of widely used *N*,*N*-dialkylamino-naphthalimides, shows poor fluorescence in polar solvents such as acetonitrile and particularly in water. In contrast, *N*-cyclohexyl derivative **10c** shows greatly enhanced fluorescence in polar solvents as well as in water. Fluorescence quantum yields determined for naphthalimides in water confirmed a dramatic change (up to 750-fold increase) from **10a** (*Φ*_F_ = 0.0004) to **10c** (*Φ*_F_ = 0.30) (Table S6, ESI†). Again, with one-photon absorption maxima around 450 nm, the naphthalimide dyes can be excited under two-photon excitation at a longer wavelength (∼900 nm) than that of acedans. However, naphthalimide dyes have been scarcely used in two-photon probes so far,^20^ probably owing to negligible TPACS values in water observed for the widely used *N*,*N*-dialkylamino-substituted naphthalimides such as **10a** (Table S12, ESI†). In this respect, naphthalimide **10c** (*δ* = 33 GM) and its analogues are highly promising for two-photon imaging applications.

Again the same trend in the quantum yields was also observed in the case of 7-aminocoumarin derivatives (**11a–11c**) (Fig. 6 and Table S6, ESI†). *N*,*N*-Diethylamino-coumarin **11a** has a very low quantum yield in water (*Φ*_F_ = 0.05), but the corresponding *N*-cyclohexyl derivative (**11c**; *Φ*_F_ = 0.59) has ∼12-fold enhanced quantum yield and thus shows strong fluorescence even in water. In the case of another D–A type of dye, NBD derivatives (**12a–12c**), again we can confirm significantly enhanced fluorescence in water (6.6-fold in the case of **12c**) as well as in polar acetonitrile (21-fold in the case of **12c**) (Fig. 6 and Table S6, ESI†).

The poor emission behaviour of the common N,N-disubstituted derivatives of 4-amino-1,8-naphthalimide, 7-aminocoumarin, NBD, and other related D–A type fluorophores has obviously limited their use as two-photon absorption dyes. However, it is now evident that these dipolar dyes can be developed into one- and, in particular, two-photon fluorescent probes and molecular tags for biological analytes in an aqueous environment.

### Evaluations of the new dyes in cells and tissues imaging

**Fig. 7:**
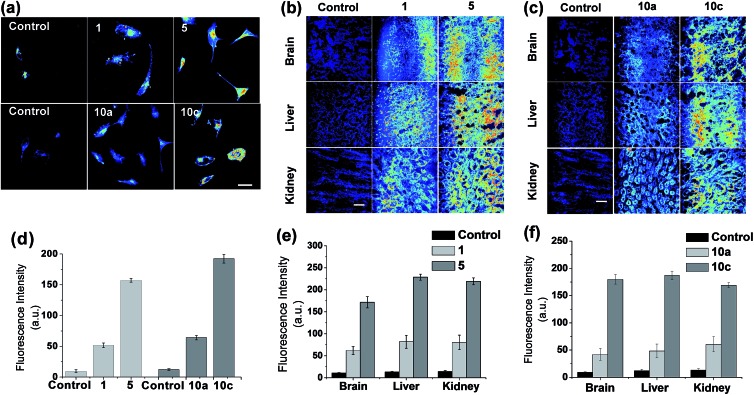
Evaluations of the new and old types of acedan and naphthalimide dyes in cell and tissue imaging by two-photon microscopy (TPM). (a) Upper row: TPM images of HeLa cells treated with acedan **1** (100 μM) and its *N*-cyclohexyl derivative **5** (100 μM) under excitation at 880 nm with 16.25 mW laser power. Lower row: TPM images of HeLa cells treated with *N*,*N*-dimethyl-naphthalimide **10a** (100 μM) and its *N*-cyclohexyl analogue **10c** (100 μM) under excitation at 900 nm with 15 mW laser power. The images were taken after incubation for 30 min. Cells untreated with any fluorophore were used as controls. (b) TPM images of mouse tissues (brain, liver and kidney) obtained after incubation with acedan **1** (100 μM) and its *N*-cyclohexyl derivative **5** (100 μM) for 10 min, under excitation at 880 nm. Tissues without treatment of any fluorophore are used as controls. Laser power: 75 mW. (c) TPM images of mouse tissues (brain, liver and kidney) obtained after incubation with naphthalimides **10a** (100 μM) and **10c** (100 μM) for 10 min, under excitation at 900 nm. Laser power: 70 mW. Scale bar: 50 μm. (d–f) Relative intensity plots of the corresponding TPM images shown in (a)–(c), which were obtained by collecting and averaging of all the data pixels. The error bars indicate ±SD.

To show an important applicability of these new dyes to fluorescence imaging of biological systems, we have further evaluated the new acedan and naphthalimide fluorophores, **5** and **10c** respectively, for imaging of cells and tissues by two-photon microscopy (TPM), in comparison with their *N*,*N*-dimethyl analogues.^21^ Through the experiments, we show that the naphthalimide dyes can be used as good or even better two-photon dyes in comparison with the acedan derivatives. Both the new acedan and naphthalimide derivatives make a marked difference from the *N*,*N*-dimethyl analogues in their cellular images when observed by TPM (Fig. 7a). From the relative fluorescence intensity data, compounds **5** and **10c** show significantly enhanced fluorescence compared to acedan and **10a**, respectively (Fig. 7d). An even more striking difference resulted when the new D–A type fluorophores **5** and **10c** were used for imaging of tissues by TPM. To lower the significant autofluorescence otherwise, we excited the acedan and its derivatives at 880 nm, rather than at 740 nm (Fig. S14 and S15, ESI†). The new acedan derivative **5** exhibits much brighter TPM images in brain, liver, and kidney tissues (Fig. 7b). As a result, 15–17-fold brighter fluorescent images with respect to that of the autofluorescence are realized with compound **5**, whereas only 5–6-fold brighter images are found with acedan (**1**) (Fig. 7e).

Naphthalimide **10c** again shows much brighter two-photon images in the brain, liver, and kidney tissues, when compared to the N,N-disubstituted derivative **10a** (Fig. 7c): 13–19-fold brighter images with respect to that of the autofluorescence are realized with **10c**, whereas only 4–5-fold brighter images are found with **10a** (Fig. 7f). The relative fluorescence intensity data highlight the dramatic substituent effect on the two-photon emission behaviour of such D–A type fluorophores.

### Applications to molecular probes for biological analytes

**Fig. 8:**
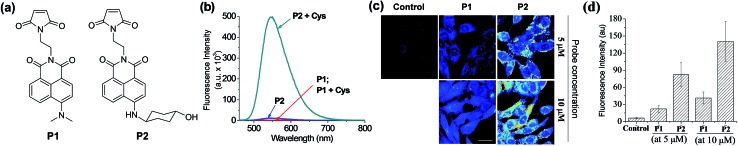
Emission properties of naphthalimide-based probes **P1** and **P2** towards thiols, and their fluorescence imaging in cells. (a) Structures of the probes, **P1** and **P2**, with different amine donors. (b) Emission spectra of **P1** and **P2** in the absence and presence of Cys (200 μM), obtained in HEPES buffer (pH = 7.4) containing 1% acetonitrile at 25 °C (excitation wavelengths for **P1** and **P2** were 440 nm and 453 nm, respectively). (c) TPM images of HeLa cells after 60 min of incubation with **P1** (middle column) or **P2** (right column) at 5 μM (upper row) and at 10 μM (lower row), obtained under two-photon excitation at 900 nm with 5.7 mW laser power. Cells without treatment of any fluorophore were used as controls. Scale bar: 30 μm. (d) Relative intensity plot of the respective TPM images shown in (c), which were obtained by collecting and averaging of all the data pixels. The error bars indicate ±SD.

To demonstrate the potential applicability of the new dyes in the development of molecular probes, finally we have synthesized two maleimides **P1** and **P2** containing *N*,*N*-dimethylamino- and *N*-(4-hydroxycyclohexyl)amino-naphthalimide dyes, respectively, for fluorescent sensing of biothiols (Fig. 8a). These thiol probes are initially in the quenched state due to the photo-induced electron-transfer (PET) process from the maleimide moiety to the naphthalimide dye, but are expected to show fluorescence upon treatment with biothiols such as cysteine (Cys) as the Michael addition of the thiol to the maleimide blocks the PET process.^22^**P2** indeed shows a large enhancement in the fluorescence upon treatment with Cys, whereas **P1** shows negligible enhancement (Fig. 8b). The contrasting results again highlight that the amine donor in dipolar dyes can play a crucial role in governing their fluorescence behaviour in aqueous media.

These probes were then applied for imaging of biothiols in HeLa cells by TPM. The HeLa cells treated with **P2** show much brighter fluorescence images than those treated with **P1** at the micromolar concentrations (Fig. 8c). The signal enhancement is caused by the Michael addition of biothiols (cysteine, glutathione, homocysteine and hydrogen sulfide) in cells to the probe. At the probe concentration of 5 μM, **P2** gives turn-on type signal enhancement (>13-fold) in sensing cellular biothiols whereas **P1** gave only threefold enhancement (Fig. 8d), again demonstrating the powerfulness of our approach to boost the two-photon emission intensity of dipolar dyes in water to a practically useful level.

## Conclusions

We have disclosed that the emission properties of dipolar fluorophores in aqueous media can be significantly altered by changing the donor amino substituent. Both theoretical computations and photophysical studies on acedan derivatives have identified the factors that affect their emission properties in water, affording highly fluorescent acedan derivatives, in particular, with greatly enhanced two-photon emission properties. We have validated that the rational approach can be generally extended to other donor–acceptor type dyes with amine donor to dramatically enhance their poor fluorescence in polar and aqueous media, as demonstrated for 1,8-naphthalimide, 7-aminocoumarin, and NBD derivatives. Furthermore, we have compared the new fluorophores with their commonly used *N*,*N*-dialkylamino forms in cell and tissue imaging and in fluorescence sensing of biothiols with the corresponding molecular probes, the results of which highlight the potential applicability of our findings to common dipolar dyes in the development of bright molecular probes, in particular two-photon probes, for biological analytes.

## Experimental section

The experimental procedures for the synthesis of all the dyes and the biothiol probes **P1** and **P2**, one-photon spectroscopic analysis, two-photon spectroscopic analysis, tissue and cell imaging experiments, and theoretical computations are described in the ESI.† The experimental procedures regarding mouse tissues herein were performed in accordance with protocols approved by the Kyung Hee University Committee on Animal Research and followed the guidelines for the use of experimental animals established by The Korean Academy of Medical Science. We made every effort to minimize animal suffering and reduce the number of animals used to prepare samples for imaging (for details of sample preparation, see ESI†).

## Supplementary Material

Supplementary information
