# Supplementary material for: A structural remedy toward bright dipolar fluorophores in aqueous media
Source: Chem Sci. 2015 May 18;6(7):4335–42. doi: 10.1039/c5sc01076d (PMC5707477; doi:10.1039/c5sc01076d)
Supplement: Supplementary file 1 [file SC-006-C5SC01076D-s001.pdf]

Electronic Supplementary Material (ESI) for Chemical Science.
This journal is © The Royal Society of Chemistry 2015

Electronic Supplementary Information

A Structural Remedy toward Bright Dipolar Fluorophores in Aqueous Media

Subhankar Singha,[a] Dokyoung Kim,[a] Basab Roy,[a] Sunderraman Sambasivan,[a] Hyunsoo Moon,[a] Alla Sreenivasa Rao,[a] Jin Yong Kim,[a] Taiha Joo,[a] Jae Woo Park,[a] Young Min Rhee,[a] Taejun Wang,[b] Ki Hean Kim,[b] Youn Ho Shin,[c] Junyang Jung,[c] and Kyo Han Ahn[a,]*

[a]Department of Chemistry, Pohang University of Science and Technology (POSTECH), 77 Cheongam-Ro, Nam-Gu, Pohang, Gyungbuk, Republic of Korea, 790-784.
[b]Division of Integrative Biosciences and Bio-technology, Pohang University of Science and Technology (POSTECH), 77 Cheongam-Ro, Nam-Gu, Pohang, Gyungbuk, Republic of Korea, 790-784.
[c]Department of Anatomy and Neurobiology, School of Medicine, Biomedical Science Institute, Kyung Hee University, 26 Kyungheedae-Ro, Dongdaemun-Gu, Seoul Republic of Korea, 130-701.
*E-mail: ahn@postech.ac.kr

Contents **Page No**

One-photon spectroscopic analysis……………………………………………………………..................................S2-S7
- Absorption spectra of acedan (**1**) and its derivatives **2**–**9**…………………………………………………………….S2-S3
- Emission spectra of acedan (**1**) and its derivatives **2**–**9**……………………………………………………………………S4
- Lippert-Mataga plot for acedan (**1**) and its derivatives **2**–**9**……………………………………………………………S5
- Absorption and emission spectra of naphthalimides **10a**–**10c**……………………………………………………………S6
- Absorption and emission spectra of coumarin derivatives **11a**–**11c**……………………………………………………S6
- Absorption and emission spectra of NBD derivatives **12a**–**12c**…………………………………………………………S7

Fluorescence quantum yield measurement …………………………………………………………………………S7-S8
- Quantum yields of acedan (**1**) and its derivatives **2**–**9** in different solvents……………………………………S8
- Quantum yields of naphthalimides (**10a**–**10c**), coumarins (**11a**–**11c**) and NBDs (**12a**–**12c**)………………S8

Fluorescence life-time measurement of acedan (**1**) and its derivatives **5−7**..………………………………......S8-S10
Emission spectra of additional acedan derivatives **13**–**18**………………………………………………………………S10
Two-photon spectroscopic analysis……………………………………………………………………………………S10-S11
- Two-photon absorption cross-sections (δ) of acedan (**1**) and its derivatives **5**–**7** in different solvents…..S10
- Two-photon absorption cross sections (δ) of naphthalimides **10a**–**10c** in different solvents…………………S11

Exclusion of a possibility of nanoparticle formation in this study……………………………………………………S12
Two-photon fluorescence microscopic imaging…………………………………………………………………….S12-S13
Preparation of cell lines for imaging…………………………………………………………………………………..S14
Preparation of mouse tissue samples for imaging……………………………………………………………………S14
Cell viability assay………………………………………………………………………………………………………...S14
Theoretical computations………………………………………………………………………………………...S15–S17
- Computational method, supporting results and discussions…………………………………………...S15-S17

Estimation of the relative contribution of the three factors to emission properties in aqueous media……………………………………………………………………………………………………...S18
Synthetic schemes and experimental procedures for compound **2-18**, **P1** and **P2**………………………S19–S30
References………...S30
^{1}H NMR and ^{13}C NMR spectra ……………………………………………………………………………S31-S52

One-photon spectroscopic analysis

UV/Vis absorption spectra were obtained using a HP 8453 UV/Vis spectrophotometer. Fluorescence spectra were recorded on a Photon Technology International fluorimeter with a 10 mm cuvette. The excitation and emission wavelength band paths were both set at 2 nm. Stock solutions of each dye were prepared by dissolving them separately in each solvent used (each 1.0 mM). In case of an aqueous solution, a stock solution was made in DMSO (1 mM) and it was added to the aqueous solvent by keeping the concentration of DMSO within 1% of total volume. Final titrant volume is the same for all measurement (3 mL).

Absorption spectra of acedan (1) and its derivatives 2–9 in different solvents

HEPES buffer (pH 7.4)

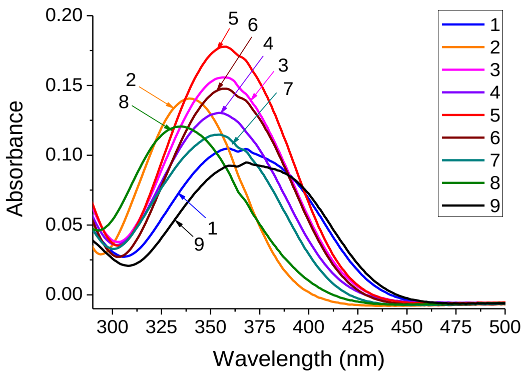

Water

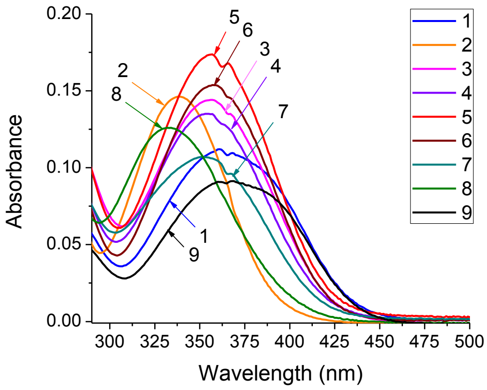

Ethanol

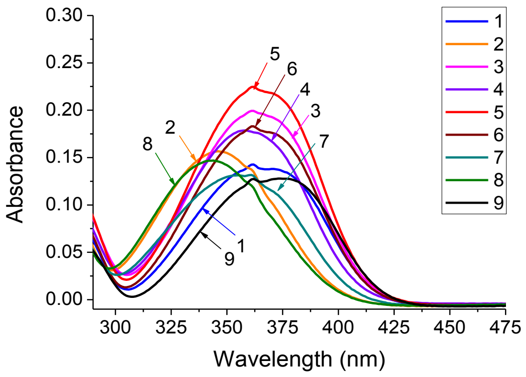

Acetonitrile

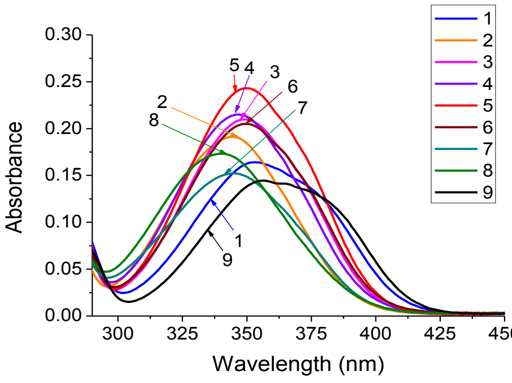

Dimethylformamide

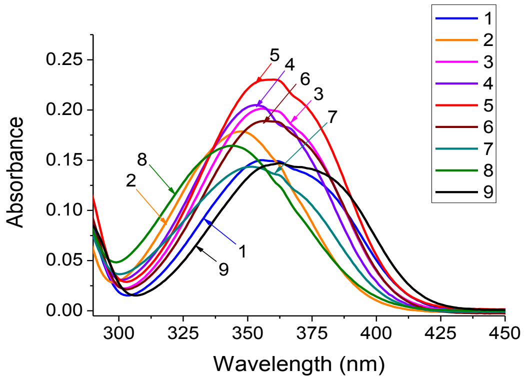

Dichloromethane

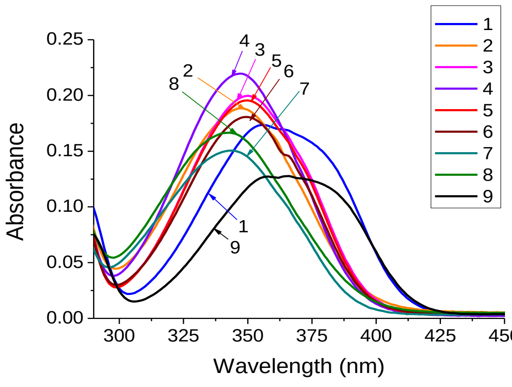

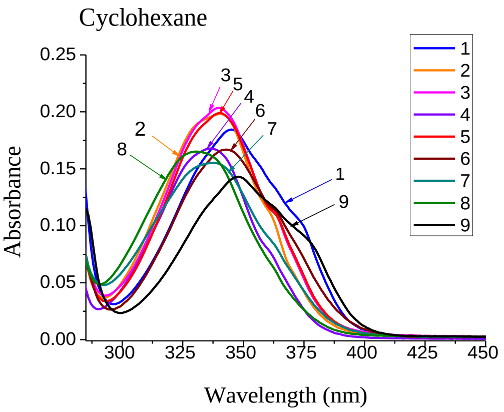

Fig. S1 Absorption spectra of acedan (**1**) and its derivatives **2**–**9** in different solvents (HEPES buffer, water, ethanol, acetonitrile, DMF, dichloromethane, and cyclohexane). Absorption spectra were obtained by dissolving each compound at a concentration of 10^{-5} M in the corresponding spectroscopic grade solvent.

Table S1 Maximum absorbance wavelength [λ_{abs} (nm)] of acedan (**1**) and its derivatives **2**–**9** in different solvents[†]

Compound / Solvent	**1**	**2**	**3**	**4**	**5**	**6**	**7**	**8**	**9**
HEPES buffer[‡]	360	339	357	355	358	358	355	335	368
Water	362	338	357	355	357	357	351	332	368
EtOH	362	346	362	358	362	362	355	344	374
Acetonitrile	353	345	350	347	350	350	345	340	357
DMF	355	347	355	353	360	357	352	344	362
CH_2Cl_2	356	348	350	347	350	350	344	343	358
Cyclohexane	345	341	340	337	340	343	338	330	348

[†]All data were obtained for a solution (10^{-5} M) of each compound dissolved in different solvent. [‡]HEPES buffer of pH 7.4 was used.

Table S2 Molar extinction coefficient [ε (L mol^{-1} cm^{-1})] of acedan (**1**) and its derivatives **2**–**9** in different solvents

Compd. / Solvent	**1**	**2**	**3**	**4**	**5**	**6**	**7**	**8**	**9**
HEPES Buffer[†]	10500	14100	15600	13000	17800	14800	11500	12100	9500
Water	11200	14600	14500	13500	17400	15400	11000	12600	9100
EtOH	14300	15700	19900	17900	22500	18300	13100	14700	12800
CH_3CN	16400	19100	21000	21500	24300	20500	15200	17300	14500
DMF	15000	17900	20100	20500	23000	18900	14300	16400	14700
CH_2Cl_2	17400	18800	20000	22000	19600	18100	15100	16700	12700
Cyclohexane	18400	19800	20300	16800	19900	16700	15500	16500	14300

[†]HEPES buffer of pH 7.4 was used.

Emission spectra of acedan (1) and its derivatives 2–9 in different solvents

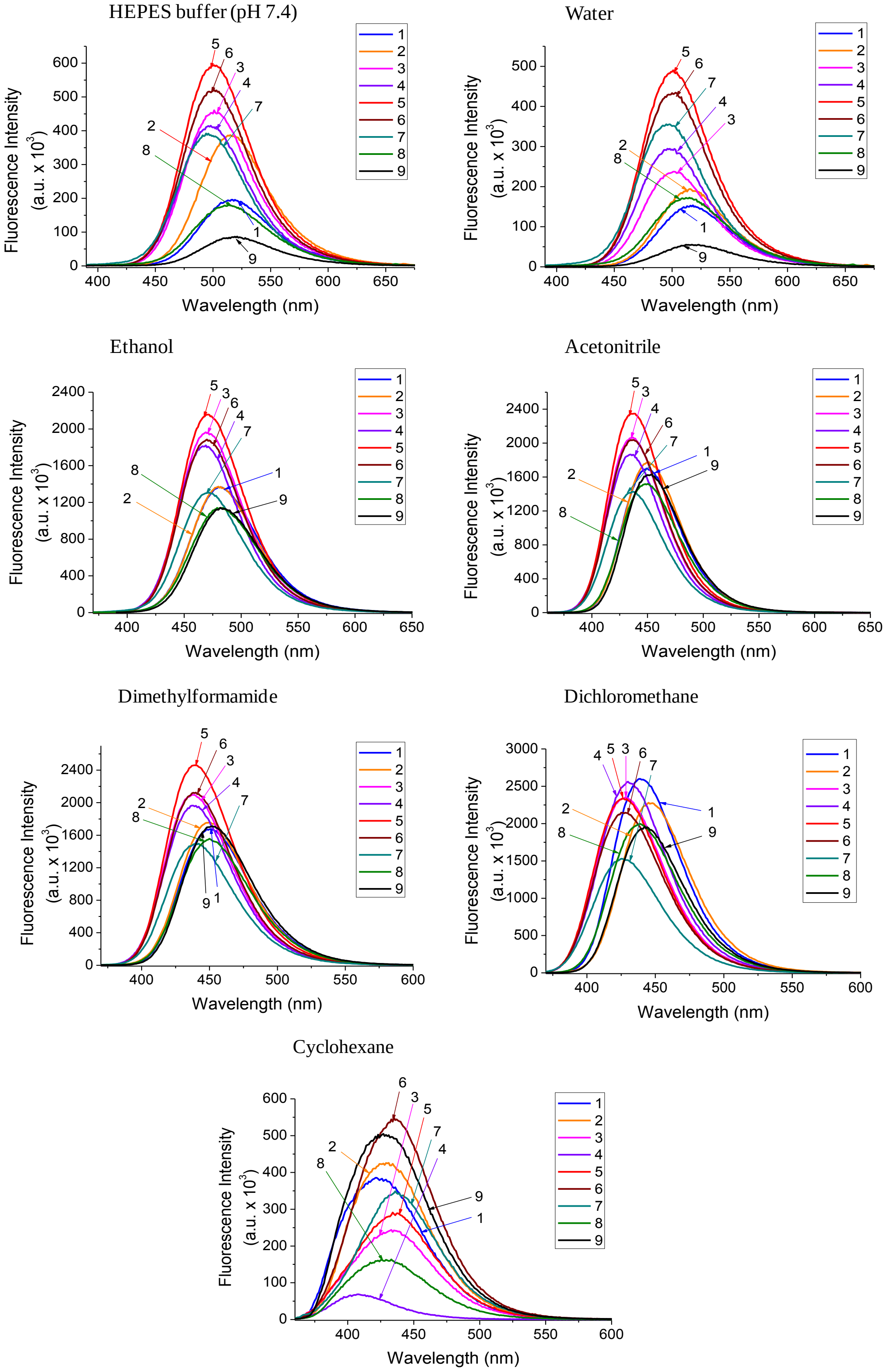

Fig. S2 Emission spectra of acedan (**1**) and its derivatives **2**–**9** in different solvents (HEPES buffer, water, ethanol, acetonitrile, DMF, dichloromethane, and cyclohexane). Emission spectra were obtained by dissolving each compound at a concentration of 10^{-5} M in the corresponding spectroscopic grade solvent by excitation at the maximum absorbance wavelength (λ_{abs}) of each compound.

Table S3 Maximum emission wavelength [λ_{em} (nm)] of acedan (**1**) and its derivatives **2**–**9** in different solvents[†]

Compound / Solvent	**1**	**2**	**3**	**4**	**5**	**6**	**7**	**8**	**9**
HEPES buffer[‡]	516	515	501	501	503	503	496	514	519
Water	516	516	501	501	503	503	495	515	517
EtOH	482	480	469	469	471	470	472	484	482
Acetonitrile	449	451	437	435	438	437	436	450	454
DMF	451	450	437	438	438	438	440	450	452
CH_2Cl_2	439	448	428	430	426	428	425	439	442
Cyclohexane	422	427	433	408	438	435	436	433	427

[†]Excited at the maximum absorbance wavelength (λ_{abs}) of each compound. [‡]HEPES buffer of pH 7.4 was used.

Lippert-Mataga plot for acedan (1) and its derivatives 2–9

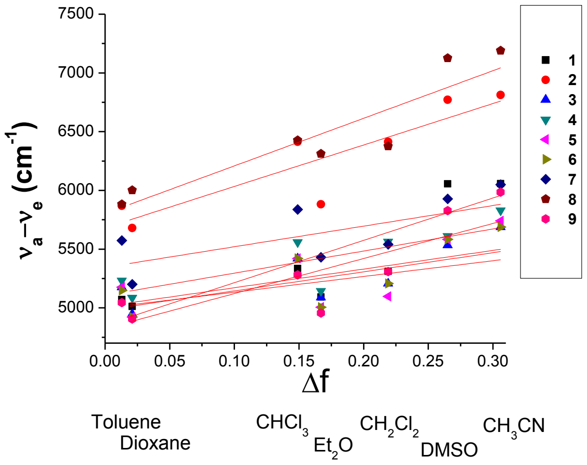

Fig. S3 Lippert-Mataga plot for acedan and its derivatives **1**–**9** in aprotic solvents (Toluene, dioxane, chloroform, diethyl ether, dichloromethane, dimethylsulfoxide and acetonitrile), where ν_a and ν_e mean the maximum absorbance wavenumber and the maximum emission wavenumber, respectively, and Δf means the solvent polarity factor.

The plots show significant deviation both in diethyl ether and in dichloromethane, but apparently give an increment of the slope as the medium polarity increase. Compounds with *N*,*N*-dialkyl-substituted-amine donors are differentiated from those with *N*-monoalkyl-substituted ones; the former derivatives (**1**, **2**, **8**, and **9**) have larger slopes than the latter compounds (**3**–**7**).

Absorption and emission spectra of naphthalimides 10a–10c

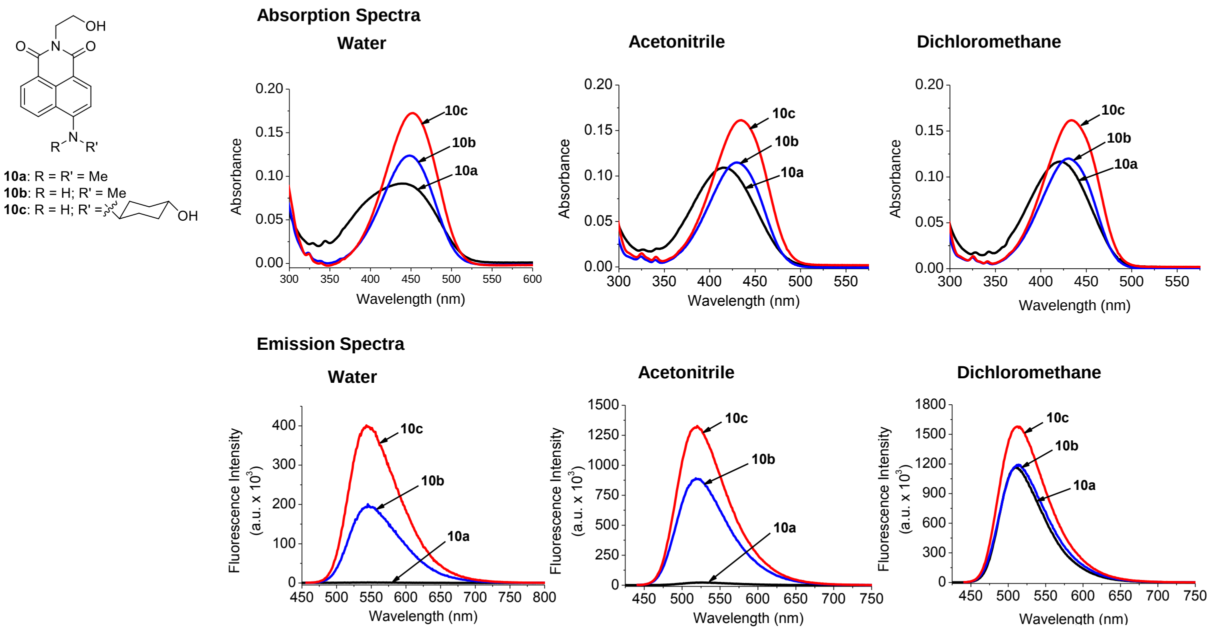

Fig. S4 Absorption and emission spectra of naphthalimide derivatives **10a**–**10c** in different solvents (water, acetonitrile, and dichloromethane). Spectra were obtained by dissolving each compound at a concentration of 10^{-5} M in the corresponding spectroscopic grade solvent. The emission spectra were obtained by exciting each molecule at the maximum absorption wavelength (λ_{abs}) in the corresponding solvent.

Absorption and emission spectra of coumarin derivatives 11a–11c

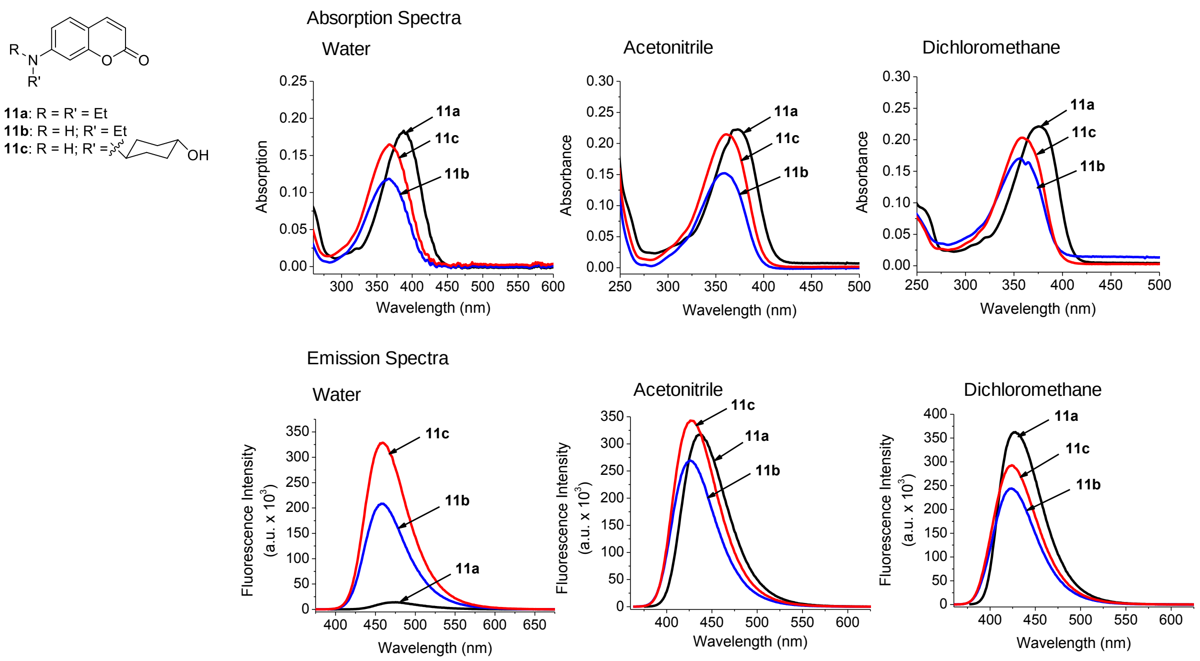

Fig. S5 Absorption and emission spectra of coumarin derivatives **11a**–**11c** in different solvents (water, acetonitrile, and dichloromethane). Spectra were obtained by dissolving each compound at a concentration of 10^{-5} M in the corresponding spectroscopic grade solvent. The emission spectra were obtained by exciting each molecule at the maximum absorption wavelength (λ_{abs}) in the corresponding solvent.

Absorption and emission spectra of NBD derivatives 12a–12c

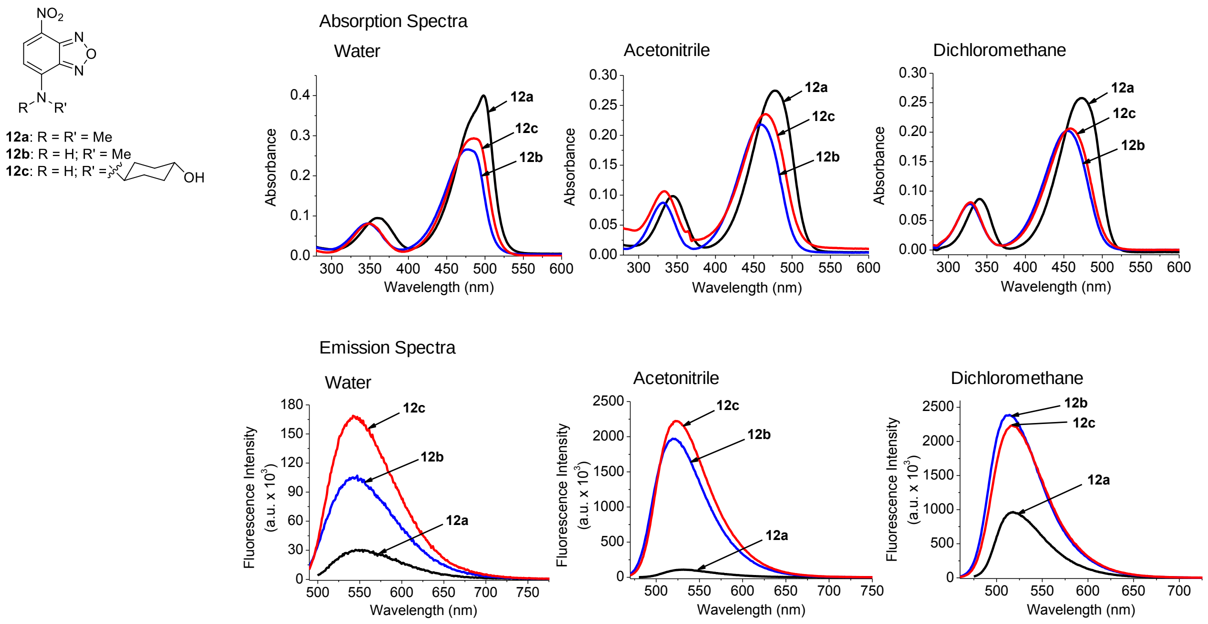

Fig. S6 Absorption and emission spectra of NBD derivatives **12a**–**12c** in different solvents (water, acetonitrile, and dichloromethane). Spectra were obtained by dissolving each compound at a concentration of 10^{-5} M in the corresponding spectroscopic grade solvent. The emission spectra were obtained by exciting each molecule at the maximum absorption wavelength (λ_{abs}) in the corresponding solvent.

Table S4 Molar extinction coefficient [ε (L mol^{-1} cm^{-1})], the maximum absorbance [λ_{abs} (nm)] and emission wavelength [λ_{em} (nm)] of compounds **10a–10c**, **11a–11c** and **12a–12c** in different solvents†

Solvent / Compd.	Water			Acetonitrile			Dichloromethane		
	ε	λ_{abs}	λ_{em}	ε	λ_{abs}	λ_{em}	ε	λ_{abs}	λ_{em}
**10a**	9160	440	555	10900	416	526	11700	421	509
**10b**	12400	448	545	11500	430	518	12000	431	512
**10c**	17300	453	543	16100	435	520	16200	434	513
**11a**	18300	388	475	22300	373	436	22100	375	426
**11b**	11850	368	458	15200	359	426	17050	356	424
**11c**	16500	368	460	21500	361	426	20300	358	424
**12a**	40000	498	556	27400	478	529	25800	473	518
**12b**	26600	478	542	21800	460	519	20250	455	514
**12c**	29300	486	542	23500	466	522	20600	459	517

†Excited at the maximum absorbance wavelength (λ_{abs}) of each compound.

Fluorescence quantum yield measurement

The fluorescence quantum yields were measured by using rhodamine B, rhodamine 6G, and coumarine 47 as references (1), because two-photon absorption cross sections have to be measured as well. The sample solutions were excited by a laser light with the wavelengths tuned to 370, 380, and 390 nm, and fluorescence was detected by a spectrograph equipped with a CCD detector. The full emission spectra were measured and integrated to give the quantum yields. Rhodamine B was employed as a primary reference because it is widely used as a reference for the two-photon absorption cross section measurement (2). Quantum yields of coumarin 47 (also called coumarin 460 or Coumarin 1 which have absorptions and emissions in the similar range that of acedan derivatives) and rhodamine 6G were cross-checked using rhodamine B as the reference to avoid any uncertainty that may arise due to the weak absorption of rhodamine B at those wavelengths. After confirming no uncertainty

in the calculated quantum yield of coumarin 47 and rhodamine 6G compared with literature values, we measured the quantum yields of all the acedan, naphthalimide, coumarin and NBD derivatives using rhodamine B as reference.

Table S5 Quantum yields of acedan (**1**) and its derivatives **2**–**9** in different solvents.

Compound	Φ_F†		
	CH_2Cl_2	CH_3CN	H_2O
**1**	0.48	0.52	0.20
**2**	0.44	0.47	0.36
**3**	0.41	0.43	0.31
**4**	0.35	0.47	0.32
**5**	0.40	0.51	0.40
**6**	0.37	0.52	0.39
**7**	0.31	0.44	0.31
**8**	0.53	0.54	0.23
**9**	0.48	0.53	0.09

†Fluorescence quantum yield determined using rhodamine B as standard. The uncertainty is less than ±10%.

Table S6 Quantum yields of naphthalimides (**10a**–**10c**), coumarins (**11a**–**11c**) and NBDs (**12a**–**12c**) in different solvents

Compound	Φ_F†		
	CH_2Cl_2	CH_3CN	H_2O
**10a**	0.74	0.015	0.0004
**10b**	0.79	0.57	0.21
**10c**	0.73	0.61	0.30
**11a**	0.43	0.34	0.05
**11b**	0.47	0.48	0.51
**11c**	0.40	0.48	0.59
**12a**	0.24	0.03	0.008
**12b**	0.59	0.54	0.04
**12c**	0.51	0.63	0.05

†Fluorescence quantum yield determined using rhodamine B as standard. The uncertainty is less than ±10%.

Fluorescence life-time measurement

The picosecond time-resolved photoluminescence was measured by employing the time-correlated single photon counting (TCSPC) technique. The excitation light source was a home-made cavity-dumped femtosecond Ti:Sapphire laser pumped by a Nd:YVO_4 laser (Verdi, Coherent). The center wavelength and spectral width of the laser output were 760 nm and 40 nm, respectively. Pump pulses at 380 nm were generated by the second harmonic generation in a 300 μm thick β-barium borate (BBO) crystal. The repetition rate and pump pulse energy were adjusted to 380 kHz and 1 nJ, respectively. The residual 760 nm output was used as trigger pulse. The TCSPC apparatus was the same as the one reported previously (3), except that a fast silicon avalanche photodiode (id100, ID Quantique SA) was used as a detector. The full width at half-maximum (FWHM) of the instrument response function was typically ～60 ps providing a time resolution of 15 ps after deconvolution. Samples were prepared with the concentration of about 10^{-4} M, and a 1 mm fused-silica cell was used for the optical measurements. The fluorescence life-time measurement was performed for selected compounds **1** and **5**–**7**.

Fluorescence life-time profiles of acedan (1) and its derivatives 5–7.

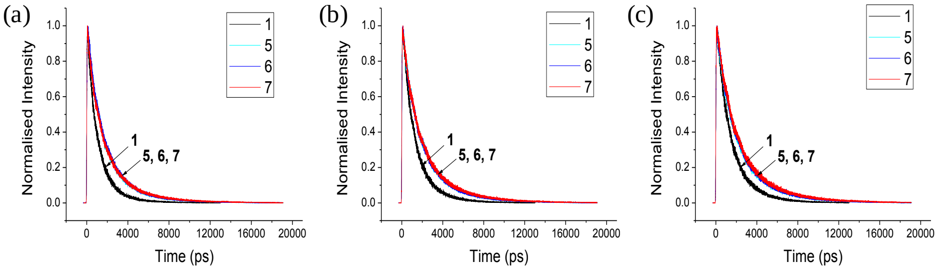

Fig. S7 Fluorescence life-time profile of acedan (**1**) and its derivatives **5−7** measured in water at different regions: (a) blue region (470 nm for acedan; 475 nm for compounds **5−7**), (b) center region (510 nm for acedan; 500 nm for compounds **5−7**), and (c) red region (560 nm for acedan; 530 nm for compounds **5−7**) of the emission spectra.

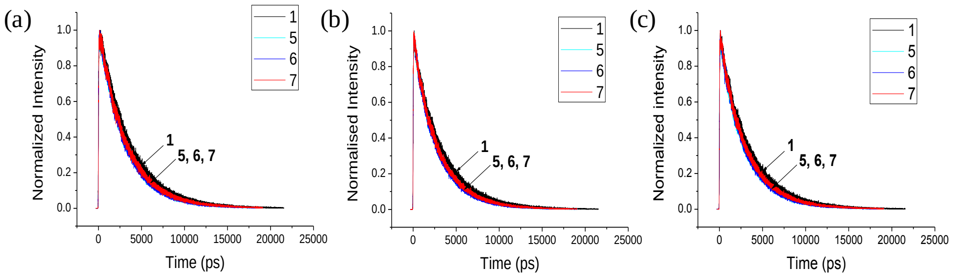

Fig. S8 Fluorescence life-time profile of acdean (**1**) and its derivatives **5−7** measured in acetonitrile at different regions: (a) blue region (425 nm for acedan; 420 nm for compounds **5−7**), (b) center region (450 nm for acedan; 440 nm for compounds **5−7**), and (c) red region (480 nm for acedan; 470 nm for compounds **5−7**) of the emission spectra.

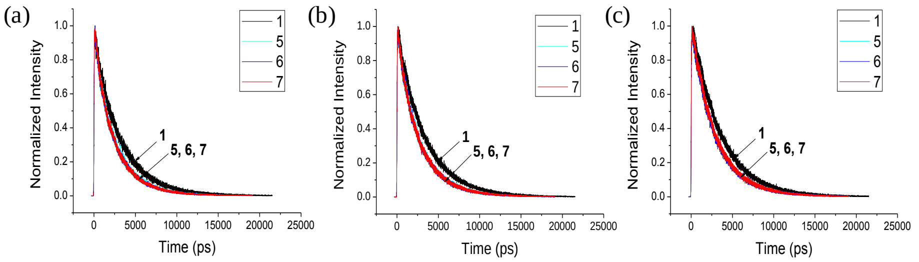

Fig. S9 Fluorescence life-time profile of acedan **(1)** and its derivatives **5−7** measured in dichloromethane at different regions: (a) blue region (420 nm for acedan; 410 nm for compounds **5−7**), (b) center region (445 nm for acedan; 425 nm for compounds **5−7**), and (c) red region (470 nm for acedan; 460 nm for compounds **5−7**) of the emission spectra.

Table S7 Average fluorescence life-time of acedan (**1**) and its derivatives **5−7** in water

Compound	Average Time (ns)†			Φ_F
	Blue	Center	Red	
**1**	1.05	1.29	1.49	0.20
**5**	2.75	3.15	3.43	0.40
**6**	2.85	3.05	3.40	0.39
**7**	2.86	3.40	3.69	0.31

†Average fluorescence life-time was measured in water at blue (470 nm for acedan; 475 nm for compounds **5**−**7**), center (510 nm for acedan; 500 nm for compounds **5**–**7**), and red (560 nm for acedan; 530 nm for compounds **5**–**7**) region of the emission spectra.

Table S8 Average fluorescence life-time of acedan (**1**) and its derivatives **5−7** in acetonitrile

Compound	Average Time (ns)†			Φ_F
	Blue	Center	Red	
**1**	2.99	3.19	3.16	0.52
**5**	2.58	2.79	3.09	0.51
**6**	2.51	2.69	3.04	0.52
**7**	2.66	2.92	2.97	0.44

†Average fluorescence life-time was measured in acetonitrile at blue (425 nm for acedan; 420 nm for compounds **5−7**), center (450 nm for acedan; 440 nm for compounds **5−7**) and red (480 nm for acedan; 470 nm for compounds **5−7**) region of the emission spectra.

Table S9 Average fluorescence life-time of acedan (**1**) and its derivatives **5−7** in dichloromethane

Compound	Average Time (ns)†			Φ_F
	Blue	Center	Red	
**1**	3.04	3.24	3.46	0.48
**5**	2.29	2.53	2.89	0.40
**6**	2.21	2.45	2.87	0.37
**7**	2.21	2.49	2.93	0.31

†Average fluorescence life-time measured in dichloromethane at blue (420 nm for acedan; 410 nm for compounds **5−7**), center (445 nm for acedan; 425 nm for compounds **5−7**) and red (470 nm for acedan; 460 nm for compounds **5−7**) region of the emission spectra.

Emission properties of additional derivatives of acedan and compound 5

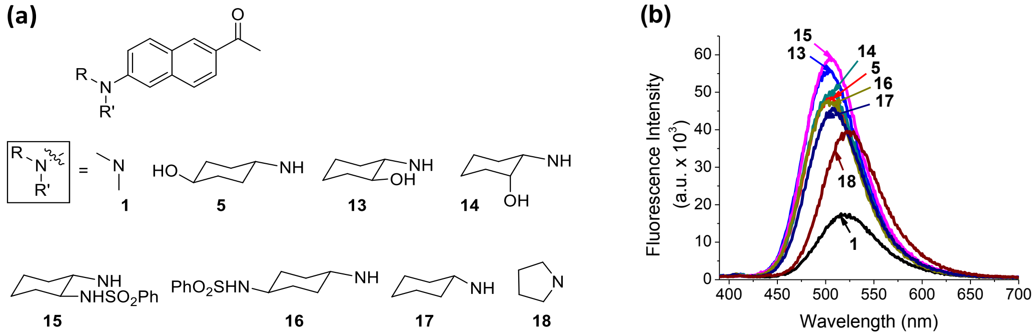

Fig. S10 (a) Acedan derivatives **13−18** investigated further. (b) Emission spectra of acedan (**1**) and its derivatives **5**, **13**–**18** (each at 1 μM) obtained in water under excitation at the maximum absorbance wavelength (λ_{abs}) of each compound.

Table S10 The maximum absorbance [λ_{abs} (nm)] and emission wavelengths [λ_{em} (nm)] of compounds **13**–**18** in water

Coumpound	**13**	**14**	**15**	**16**	**17**	**18**
λ_{abs} (nm)	360	362	375	357	357	384
λ_{em} (nm)	503	506	505	503	507	524

Two-photon spectroscopic analysis

The measurement of two-photon emission spectra was performed by using home-built cavity-dumped Ti:sapphire oscillator pumped by a 5.0 W output of a frequency-doubled Nd:YVO4 laser (Verdi, Coherent), which allows us to use different experimental excitation wavelengths. The energy of the output pulses was 40 nJ at the repetition rate of 380 kHz. A 15 cm focal length singlet lens was used to focus the excitation beam to the sample in a 1 mm quartz cell, and the fluorescence was collected in a backscattering geometry using a parabolic mirror. Two-photon cross section (GM) was checked by TPACS (Two-photon induced fluorescence) method

and 1 units (GM) value is 10^{-50} cm^4 s $photon^{-1}$ $molecule^{-1}$. Two-photon absorption cross section [δ (GM)] values were determined using rhodamine B as a standard (2).

The measurement of two-photon emission spectra was performed for selected compounds **1**, **5**–**7** and **10a**–**c**.

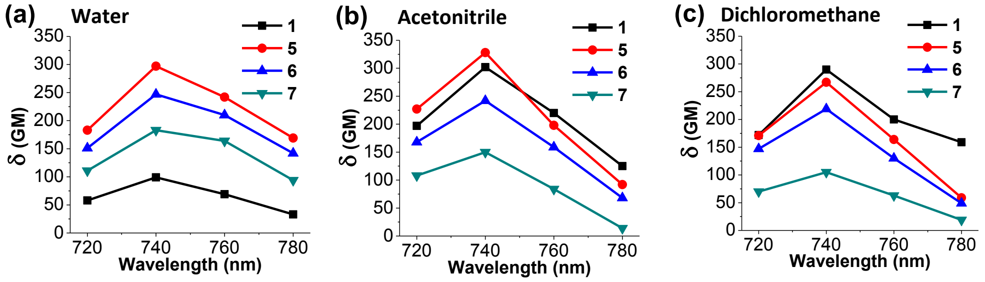

Fig. S11 Plots of two-photon absorption cross-sections (δ) of acedan (**1**) and its derivatives **5**–**7** in different solvents. The values were measured in (a) water, (b) acetonitrile, and (c) dichloromethane.

Table S11 Two-photon absorption cross-sections (δ) of acedan (**1**) and its derivatives **5**–**7** in different solvents

Compound	λ_{ex}† (nm)	TPACS (δ)‡ (GM)		
		CH_2Cl_2	CH_3CN	H_2O
**1**	740	290	302	99
	760	200	220	69
	780	159	125	33
**5**	740	267	328	297
	760	164	198	242
	780	59	92	169
**6**	740	219	242	247
	760	130	159	210
	780	49	68	142
**7**	740	105	150	183
	760	63	84	164
	780	19	14	94

†Excitation wavelength. ‡Determined using rhodamine B as a standard. The uncertainty is less than ±10%.

Table S12 Two-photon absorption cross sections (δ) of naphthalimides **10a**–**10c** in different solvents

Compound	λ_{ex}† nm	TPACS (δ)‡ (GM)		
		CH_2Cl_2	CH_3CN	H_2O
**10a**	840	63	76	ND§
	820	41	47	ND§
	800	66	74	ND§
**10b**	840	46	51	19
	820	28	34	13
	800	41	44	15
**10c**	840	43	38	33
	820	24	23	17
	800	34	33	22

†Excitation wavelength; 840 nm is the maximum wavelength available from our home-made laser. ‡Determined using rhodamine B as a standard. The uncertainty is less than ±10%. §ND = Not detectable.

Exclusion of a possibility of nanoparticle formation in this study

To check whether nanoparticles can be formed under our experimental conditions of preparing aqueous solutions of those acedan derivatives (a DMSO stock solution were mixed with water at 25 °C to make final compound concentration of 10 μM by keeping volume of DMSO at 1%), we have carried out DLS (dynamic light scattering) analyses for aqueous solutions prepared with acedan and the representitive acedan derivative **5** under the same experiemental conditions used in the above: In no cases, we were able to observe nanoparticles from the DLS analyses, confirming that we have dealt with molecular solutions, not nanoparticle solutions.

On the other hand, when we intentionally prepared a nanoparticle colloidal solution of compound **5** by following a literature procedure (a stock solution of compound **5** in water-immiscible dichloromethane at a high concentration of 200 μM was injected rapidly into water under vigorous sonication at 40 °C: Yao, Y. W.; Yan, Y.; Xue, L.; Zhang, C.; Li, G.; Zheng, Q.; Zhao, Y. S.; Jiang, H.; Yao, J. *Angew. Chem. Int. Ed.* **2013**, *52*, 8713), we were able to observe nanoparticles by DLS analysis, as shown in Fig. S12.

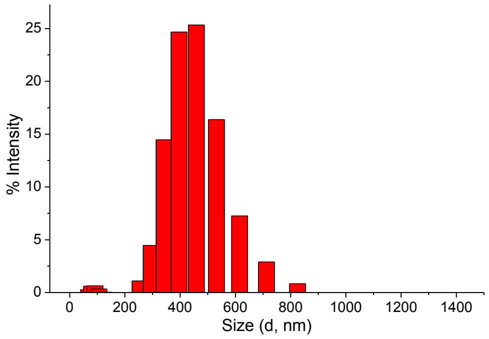

Fig. S12. DLS analysis data for a nanoparticle colloidal solution of compound **5**, prepared according to the literature procedure.

The absorption and emission spectra of the molecular solution of compound **5** were compared with those of its nanoparticle colloidal solution both at 10 μM concentration as shown below. It is clear that the nanoparticle solution gives significantly reduced fluorescence compared with the molecular solution (Fig. S13). This reduced emission is plausibly owing to the aggregation-induced fluorescence quenching effect. In other words, there is significant loss in the emission intensity by forming nanoparticle solutions of such dipolar dyes. Also, the nanoparticle solution shows a slight red-shift in the emission wavelength, as noted in the literature: λ_{em} (molecular solution) = 501 nm vs λ_{em} (nanoparticle solution) = 508 nm).

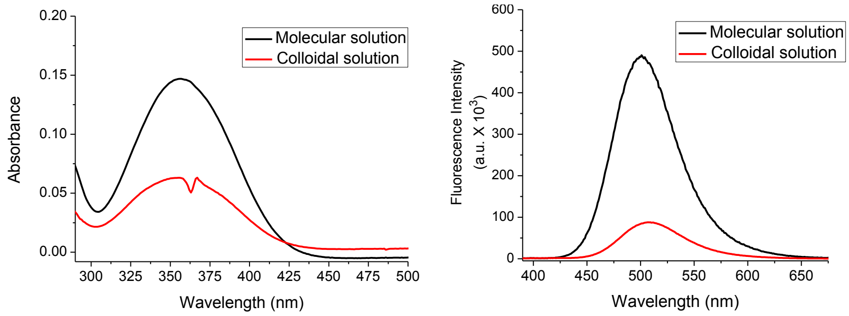

Fig. S13 Comparison of the absorption and emission spectra of the nanoparticle colloidal solution with those of an aqueous “molecular” solution of compound **5** (10 μM) used in our study.

Two-photon fluorescence microscopy imaging

Cell samples were prepared from HeLa human cervical carcinoma cells, and tissue samples were collected from C57BL6 type mouse (5 weeks, male, Samtako Co.). A two-photon microscopy (TPM) instrument used for imaging was equipped with an upright microscope (BX51, Olympus) that has 20× and 40× objective lens (XLUMPLFLN, NA 1.0, Olympus) with 1.0 numerical aperture (NA). A Ti-sapphire laser (Chameleon Ultra II, Coherent) with 140 fs pulse width and 80 MHz pulse repetition rate was used as the excitation light source. Output from the source first passed through a combination of a half wave plate and a polarizer for power control.

The objective lens focused the excitation beam into a cell and tissue samples. The excitation focus was scanned in the x-y plane of the sample by resonant (GSI Lumonics, 8 KHz resonant frequency) and galvanometric (6215H, Cambridge Technology) scanners, and in the z axis by an objective translator (P-725.4CL, PI) through the two dichroic mirrors (1025DCSP, 680DCSP, Hamamatsu). Emission light from the sample was collected back by the objective lens and was reflected on a dichroic mirror (680DCLP, Chroma) toward photomultiplier tubes (PMTs, H7421-40, Hamamatsu). Signals from the PMTs were collected by a frame grabber (Alta, Bitflow), and images were displayed in real time. Data were processed by Matlab and Amira tools. Two-photon excitation wavelength and laser powers were adjusted depending on the samples and specified when necessary.

Two-photon microscopic images of cells/tissues treated with acedan and naphthalimide derivatives

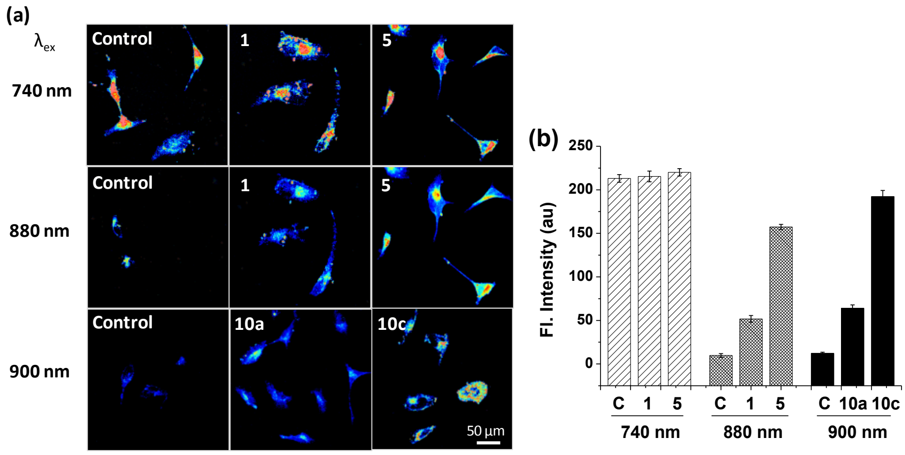

Fig. S14 (a) TPM images of HeLa cells treated with **1**, **5**, **10a** and **10c**; all at 100 µM concentration and after 30 min of incubation. For **1** and **5**, images was taken by exciting at 740 (upper row) or 880 nm (middle row) with a laser power of 17.5 mW or 16.25 mW respectively. Cells untreated with any fluorophore were used as controls. It should be noted that strong autofluorescence from the cells itself was observed when excited at 740 nm. For **10a** and **10c**, excitation wavelength was 900 nm (lower row). Laser power was 15 mW. Scale bar: 50 µm. (b) Relative intensity plot of the respective TPM images.

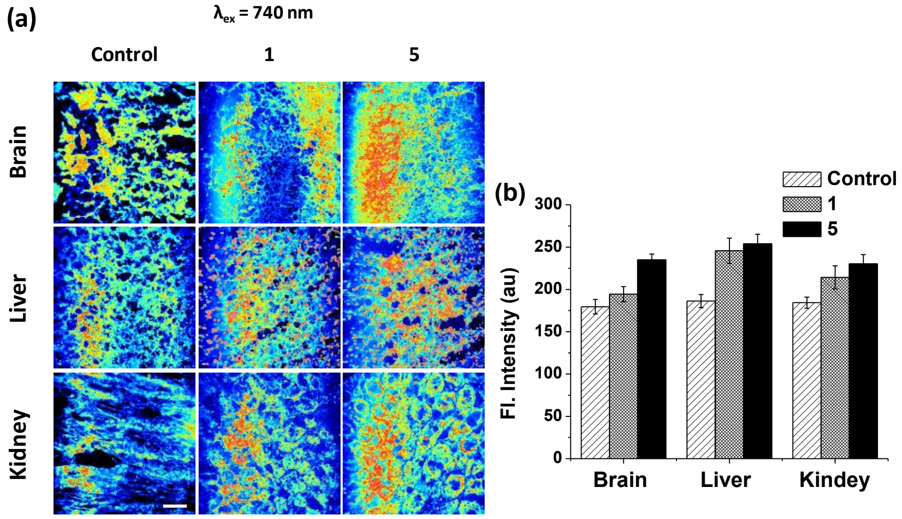

Fig. S15 (a) TPM images of brain, liver and kidney tissues treated with acedan (**1**) and its derivative **5**, both at 100 µM concentration and after 10 min of incubation. Excitation wavelength was 740 nm. Tissues without treatment of any fluorophore are used as controls. Strong autofluorescence from the tissues itself was observed. Laser power was 17.5 mW. Scale bar: 50 µm. (b) Relative intensity plot of the respective TPM images.

Preparation of cell lines for imaging

HeLa human cervical carcinoma cells were maintained in Dulbecco's Modified Eagles Medium (DMEM, Hyclone) containing 10% fetal bovine serum (Hyclone) and penicillin-streptomycin (Hyclone) at 37 ºC in a 5% (v/v) CO_2 humid incubator. Approximately, 20,000 cells/cm^2 were seed on a cover glass in each well of 24-well plate. To stain HeLa cells with fluorophores **1**, **5**, **10a** and **10c**, the cells were incubated with the corresponding fluorophores (100 μM) for 30 min. Following the incubation, the cells were washed three times with phosphate-buffer saline (PBS) and fixed with 4% paraformaldehyde (PFA) for 10 min.

For imaging of thiols in HeLa cells with probes **P1** and **P2**, the cells were incubated with the corresponding probe (5 μM and 10 μM) for 60 min. Following the incubation, the cells were washed with PBS buffer and fixed with 4% paraformaldehyde (PFA) for 10 min, and then subjected to fluorescence imaging by two-photon microscopy.

Preparation of mouse tissue samples for imaging

C57BL6 type mice (5 weeks old, male, SAMTAKO corp.) were used for this experiment. The mice were dissected to separate three organs: brain, liver and kidney. These organs were washed several times with PBS buffer. After being washed, each organ was freezed by dipping inside liquid nitrogen for 5 minutes. Then, the freezed organs were crushed into small pieces with a hammer. The small organ pieces were fixed by treatment with OCT (optimal cutting temperature medium), and then sliced to make the tissue samples using a section machine (Cryostat machine, Leica, CM3000 model) at a thickness of 16 μm. The tissue slice samples were placed on specimen blocks (Paul Marienfeld GMbH & Co.). These specimen blocks were immersed in 4% paraformaldehyde for 10 min, and then the specimen blocks were washed several times with PBS buffer. After being washed, the tissues were fixed properly on the specimen blocks using a mount solution (Gel Mount, BIOMEDA). The prepared tissue sample slides were incubated by dipping them into a solution of the acedan fluorophore (100 μM in PBS buffer) for 10 min, and then the samples were washed three times with PBS buffer and then fixed with 4% paraformaldehyde (PFA) for 10 min. When the mount solution became hard, the samples were subjected to one- and two-photon microscopic analysis to obtain the fluorescent images.

Cell viability assay

Cell viability was assessed by measuring their ability to metabolize 3-(4,5-dimethyldiazol-2-yl)-2,5-diphenyltetra-zolium bromide (MTT). Cells were seeded onto 96-well plates at a density of about 5 × 10^3 cells per well in the growth medium and incubated until about 70–80% confluency. Following the treatments as indicated, 25 μL of the MTT solution (5 mg/mL) was added to each well and cells were maintained for 2 h at 37 °C. 100 μL of solubilizing solution (4 mM HCl, 0.1% Nondet P-40 in isopropanol) was then added. After an overnight incubation at 37 °C, absorbance at 570 nm was measured.

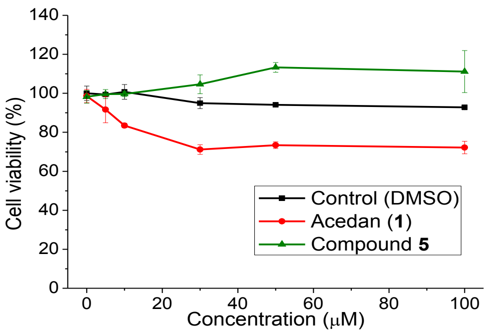

Fig. S16. Viability of cells in the presence of acedan (**1**) and its derivative **5** as measured by using MTT assay. Cells were cultured with DMSO solution as a control.

Theoretical computations

Computational methods. We have conducted molecular dynamics (MD) simulations to investigate the extent of allylic strain, hydrogen bond formation and restricted rotational freedom arround *N*-substituents for the compounds **1**, **4**, **5** and **7** with the solvating water molecules. The force field parameters for the simulations were generated in the following manner. The atomic partial charges are the crucial factors that govern the degree of hydrogen bonding as we have adopted the fixed point charge model without any polarizations. The partial charges were obtained in an identical manner for the compounds by first optimizing the geometries of **1**, **4**, **5** and **7** at the level of M06-2X with the 6-311+G** basis set and then by calculating natural atomic charges with the natural bond orbital (NBO) analysis (4, 5). Because the hydrogen bonds are very weakly affected by the choices of Lennard-Jones and internal bond parameters, we have taken the values from atoms in similar chemical environments defined in the AMBER99SB force field (6).

The simulations were initiated by solvating the geometry-optimized solute in a cubic box of 30 Å side lengths. After this, equilibration runs of 10 ps durations were conducted with the isothermal-isobaric (*NPT*) condition to reach the target temperature of 300 K and the target pressure of 1 bar. The pressure and the temperature were kept by Berendsen's weak barostat and thermostat algorithms (7). This was followed by additional 20 ps equilibration stages with the Nose-Hoover thermostat (8) and the Parrinello-Rahman barostat algorithms (9). Using these equilibrated geometries, we performed the production simulations of 10.0 ns duration for all four compounds **1**, **4**, **5** and **7**. From these trajectories, we selected 51 geometries separated by 200 ps to calculate the absorption-emission property. The oscillator strengths were calculated at the level of SOS-CIS(D_0) with the 6-31+G* basis set (10). In addition, to investigate the role of allylic strain on the transition dipole moment, we also have optimized the geometries of compounds **1**, **4**, and **5** on the first excited state potential energy surface, and calculated the transition dipole moments at the SOS-CIS(D_0)/6-31+G* level of theory. For all molecular dynamics simulations, nonbonded interactions were treated with 12.0 Å cutoffs with 10.0 Å tapers. Integration step size was 1.0 fs for all simulations. All bonds accommodating hydrogen atoms were constrained with the LINCS algorithm (11). All MD simulations were carried out with the GROMACS 4.0 program package (12). The quantum chemical calculations were performed using the developers' edition of Q-Chem 4.0 package (13).

To inspect the correlation between hydrogen bonding and the fluorescence characteristics from the MD trajectory snapshots, we need to judge whether a given solvent-solute conformation indeed has the hydrogen bonding character. For this purpose, we have adopted a simple approach of using geometrical parameters of the given conformation. Any water molecule was considered to be a candidate for hydrogen bonding when the distance between its hydrogen atom and the nitrogen in the inspected acedan derivative is below 2.27 Å and the O−H−N angle is larger than 140 deg (14, 15).

Supporting results and discussions

Allylic strain. Quantum chemically calculated transition dipoles of acedan (**1**) and its *N*-methyl analogue **4** are 1.45 D and 1.57 D, respectively. In addition, *N*-cyclohexyl acedan derivative **5** shows the transition dipole moment of 1.83 D. The torsional angles (ϕ) along with the $C_{naphthyl}$–N bond for the ground state optimized geometry are 10.47 deg (for **1**), 1.59 deg (for **4**), and 1.66 deg (for **5**). This result shows that the reduced strain with the singly substituted amine group in compounds **4** and **5** allows the molecule to have higher planarity.

This increased planarity further facilitates the charge transfer from the aromatic group and induces the increase in the transition dipole.

Hydrogen bonding and rotational freedom. Molecular dynamics (MD) simulations directly reproduced the solvation and the internal rotation effects suggested in the manuscript; namely, in the case of compound **5** that has a hydrophobic and bulky substituent, the H-bonding on nitrogen is rather suppressed and the internal rotation is largely hindered compared with the case of acedan. The degree of H-bonding is revealed by the radial distribution functions (RDFs) of hydrogen atoms in water molecules around the nitrogen atoms. The RDFs of hydrogen atoms in water molecules around the nitrogen atoms in the inspected compounds show that the solvent molecules are more highly structured around acedan and compound **4** in the first solvation shell (Fig. S17). On the other hand, the solvent molecules are less structured around the compounds **5** and **7**, plausibly due to the steric restrictions in **5** and intramolecular hydrogen bonding in **7**, respectively. One should expect that the higher solvation structure will induce better hydrogen bonding, which will subsequently shift the electron distribution to affect the fluorescence characteristics as well as provide a solvent-associated, nonradiative decay route in general.

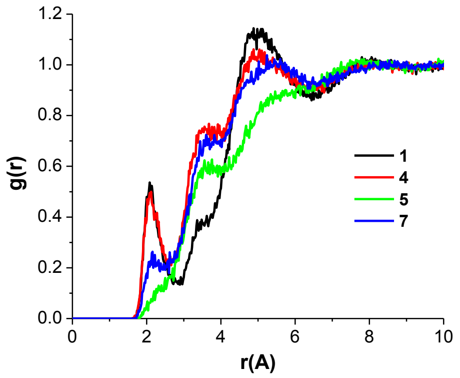

Fig. S17. Radial distribution function (RDF) of hydrogen atoms in water molecules around the nitrogen atoms of compounds acedan (**1**) and its derivatives **4**, **5** and **7** where g(r) represents RDF and r(A) means the radius in angstrom.

For more detailed analysis, we have counted the number of water molecules that may be forming hydrogen bond with the nitrogen atom from the total of 51 snapshots along the MD trajectories. Indeed, many snapshots from acedan (**1**) and compound **4** show one or two nearby water molecules, whereas majority of the snapshots from compounds **5** and **7** are accompanied by no potentially H-bonding water (Table S13). Thus, the H-bonding is less feasible with compound **5**, likely due to the high steric constraint and for compound **7** where the nitrogen atom is intramolecularly hydrogen-bonded**.**

Table S13 Number of snapshots during MD simulations with various appearances of candidate water molecules that can form hydrogen bonds with acedan (**1**) and its derivatives **4**, **5**, and **7**, together with the group-averaged oscillator strengths *f*.

	**1**		**4**		**5**		**7**	
Water	snapshots	*f*	snapshots	*f*	snapshots	*f*	snapshots	*f*
0	11*	0.15§	18*	0.18§	39*	0.11§	40*	0.1§
1	24†	0.05§	25†	0.05§	12†	0.04§	10†	0.03§
2	16‡	0.02§	8‡	0.05§	-	-	1‡	0.02§

*Number of snapshots with no water molecules satisfying the hydrogen bonding geometry criteria around the N atoms. †Number of snapshots with one water molecule satisfying the same hydrogen bonding geometry criteria. ‡Number of snapshots with two water molecules satisfying the same criteria. §Averages from the corresponding snapshots within the given group.

Computing the average oscillator strengths of acedan and compounds **4**, **5** and **7** with the surrounding water molecules indeed shows that there is a good correlation between the solvation and the fluorescence strength. Namely, when there are more surrounding water molecules, the oscillator strength decreases substantially. Therefore, compound **5**, with less H-bonding probability due to high steric constraint, will exhibit enhanced fluorescence in the excited state, and the data shown in the Table S13 supports that the H-bonding between the

nitrogen in acedan (and its derivatives) and water indeed suppresses the fluorescence. Suppressing the H-bonding around the amine nitrogen thus reduces the water-associated nonradiative decay including the initial solvent reorganization process required for attaining the ICT excited state, plausibly the TICT state.

Another aspect that strongly affects the fluorescence is the relative prevalence of the competing nonradiative decay paths. The torsional twisting around the nitrogen atom is likely to be related to the nonradiative decay. Comparing the torsional angle (ϕ) distributions obtained with MD simulations for the two acedan compounds (Fig. S18), we can see that the dihedral angle distribution become broader in acedan (**1**) compared with that in compound **5**. In addition, the distributions suggest that the rotational free energy barrier will be much lower with acedan, which, in turn, suggests that the internal rotation and the associated nonradiative decay are more feasible with acedan than with compound **5**. Even though this is a rough analysis as our MD simulations only involved ground state parameters and did not incorporate the actual nonadiabatic and nonradiative decay processes, we can at least infer that the fluorescence from acedan will be further suppressed due to the more favorable twisting around the nitrogen atom than that of compound **5**.

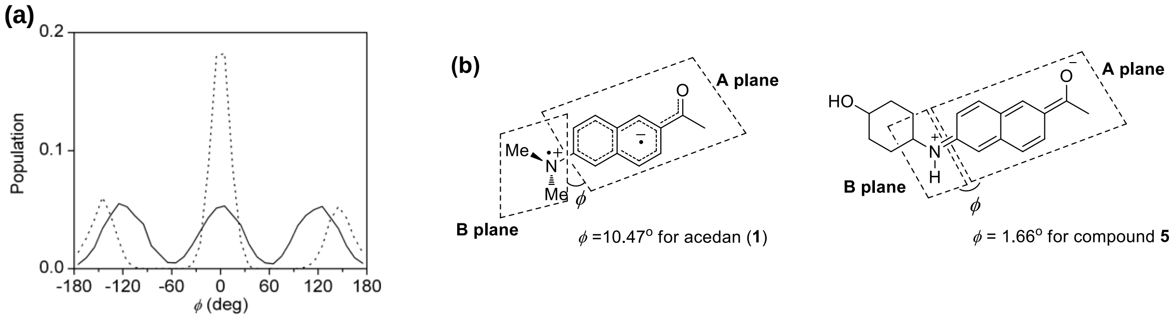

Fig. S18 (a) The distribution of torsional angle (ϕ) around the $C_{naphthyl}$–N bond in acedan (**1**) (solid) and its derivative **5** (dotted) from MD simulations. ϕ is denoted as the torsional angle around the $C_{naphthyl}$–N bond between the two plane, namely the aromatic plane of naphthyl ring (A plane) and the plane of R-N-R' (B plane). (b) Drawings of acedan (**1**) and compound **5** that show ϕ values for the optimized conformation at the ground state.

The two factors, selective suppression of the nitrogen H-bonding and reduction of the rotational freedom of the *N*-substituent, cooperatively enhance the fluorescence nearly by a factor of two, showing the largest transition moment of 1.83 D with **5** among acedan derivatives. We have indeed observed threefold-enhanced fluorescence in water in the case of **5** from that of acedan, owing to an additional increase in the absorbance when going from **1** to **5.** This absorbance increase is likely due to the reduced 1,3-allylic strain, as pointed out above, which promotes the electron delocalization from the nitrogen to the naphthalene moiety.

Estimation of the relative contribution of the three factors (allylic strain, hydrogen bonding and rotational freedom) to emission properties in aqueous media

It is difficult to separate the effect of one factor from others and also the emissive sate is still under debate. By a rough analysis below, it seems that the allylic strain plays the major role in water.

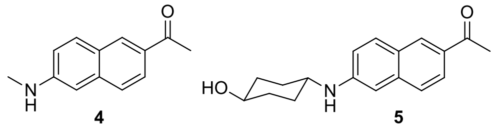

4 5

Comparison #1. Let's compare two acedan derivatives **4** and **5**, which belong to *N*-monoalkyl derivatives; the latter shows approximately 1.5-fold enhanced fluorescence in water compared with the former (Fig. 3b). In both cases, we may assume that there is a negligible difference in the allylic strain factor. Hence the remaining factors, the rotational freedom and "*N*-hydrogen bonding," are expected to play more favorably in the case of compound **5** that has a bulky and hydrophobic amino substituent. Here, we noted as *N*-hydrogen bonding to specifically indicate the water hydrogen bonding to the nitrogen lone pair, which would reduce the intramolecular charge-transfer (ICT). Thus, we may roughly say that two factors (rotational freedom + hydrogen bonding) cause 1.5-fold fluorescence enhancement.

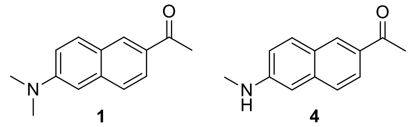

1 4

Comparison #2. Let's compare acedan (**1**) with the *N*-methylamino analogue **4**; the latter shows approximately twofold-enhanced fluorescence compared with acedan in water (Fig. 3b). Here, acedan **1** has the larger allylic strain than that of compound **4**. Next, if we compare the steric bulkiness of the two amino groups, compound **4** is expected to have the rotational freedom less than acedan, although the difference may not be large. Finally, we can assume that both the compounds may have the *N*-hydrogen bonding equally because the nitrogen lone pair in each case is not significantly buried in a hydrophobic environment. The hydrogen bonding to the NH hydrogen is possible in the case of compound **4**, which may exert any effect on the emission behaviour but not significant (the hydrogen bonding would enhance the ICT a little but also it provides a solvent-mediated relaxation channel; thus, the overall effect would be somewhat cancelled). Thus, we may roughly say that the two factors (allylic strain + rotational freedom) cause 2-fold fluorescence enhancement.

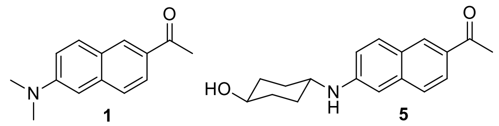

1 5

Comparison #3. Finally, let's compare compound **5** with acedan (**1**); the former shows 3-fold fluorescence enhancement compared with the latter in water (Fig. 3b). Here, compound **5** has more favorable features over acedan **1** with respect to the three factors; thus, we may say that the three factors combined (allylic strain + rotational freedom + hydrogen bonding) cause 3-fold fluorescence enhancement.

If we compare the three comparisons with respect to the enhancement factors depending on the combined structural factors [(rotational freedom + hydrogen bonding) => 1.5-fold; (allylic strain + rotational freedom) => 2-fold; (allylic strain + rotational freedom + hydrogen bonding) => 3-fold], roughly we can deduce the relative contribution of the three factors to the fluorescence enhancement in water decreases in the order: the allylic strain factor (~1.5-fold)> the hydrogen bonding factor (~1-fold) > the rotational freedom factor (~0.5-fold). *This order, however, is derived under the several approximations and thus it may not represent the actual contribution properly*. Still, we think that the allylic strain factor plays the major role in governing the emissive state. Finally, it should be noted that the structural effects are dependent on the emissive states, which, in turn, are dependent on the media.

Synthetic schemes and experimental procedures for compound 2–18, P1 and P2

General methods. Chemicals from Sigma-Aldrich were used without further purification. All reactions were performed under argon atmosphere unless otherwise stated. Analytical TLC was performed on Merck silica gel (60 F254) plates (0.25 mm) and visualized with ultraviolet light. ^{1}H and ^{13}C NMR spectra were measured with a Bruker DPX-300 spectrometer. Coupling constants (*J* value) are reported in Hertz. The chemical shifts (δ) are shown in ppm, multiplicities are indicated by s (singlet), d (doublet), t (triplet) and m (multiplet). Spectra are referenced to residual chloroform (7.26 ppm, ^{1}H, 77.16 ppm, ^{13}C), methanol (3.31 ppm, ^{1}H), acetonitrile (1.94 ppm, ^{1}H), dimethyl sulfoxide (2.50 ppm, ^{1}H; 39.52 ppm, ^{13}C). High-resolution mass spectra was recorded on a JEOL JMS-700 spectrometer at the Korea Basic Science Center, Kyungpook National University and the values are reported in units of mass to charge (*m*/*z*). Melting points were measured using an electro-thermal MELT-TEMP 3.0 instrument and uncorrected. FTIR spectra were recorded at a spectral resolution of 4 cm^{-1} with a BRUKER VERTEX 70 spectrometer.

Compounds **4**, **7**, **6a**, **10** and **11** were synthesized according to the previously reported procedures (17–20).

Scheme S1.

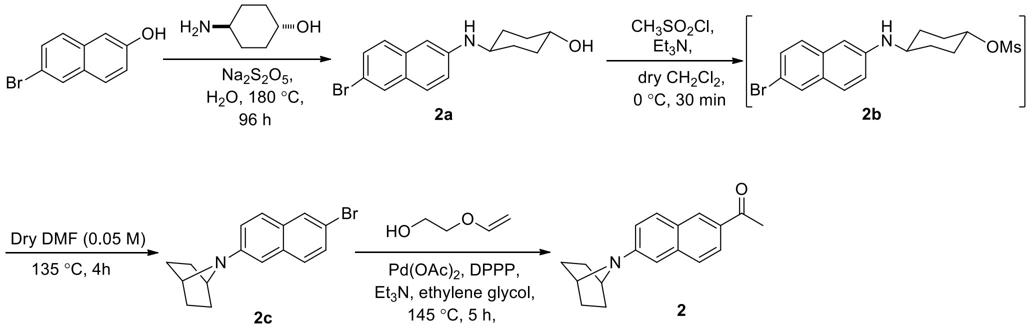

Preparation of compound 2a. 6-Bromo-2-naphthol (1.5 g, 6.72 mmol), *trans*-4-aminocyclohexanol (1.55 g, 13.45 mmol), sodium metabisulfite (2.56 g, 13.45 mmol) were taken in a sealed tube. Then 15 mL of distilled water was added to it and the mixture was heated to 180 °C for 96 h. After cooling to room temperature, the reaction mixture was diluted with ethyl acetate (300 mL), and washed sequentially with water (80 mL), 5% aqueous $NaHCO_3$ solution (50 mL), and brine. The organic layer was dried over anhydrous Na_2SO_4, filtered and concentrated in vacuo. The residue was purified by silica gel column chromatography (Hexane/EtOAc = 19/1) to give **2a** as brown solid (600 mg, 38%; 27% of 6-bromo-2-naphthol is recovered): mp 139–141 °C; ^{1}H NMR ($CDCl_3$, 300 MHz, 298 K) δ 7.79 (d, *J* = 1.5 Hz, 1H), 7.53–7.38 (m, 1H), 6.84 (dd, *J* = 8.7, 2.1 Hz, 1H), 6.74 (d, *J* = 2.1 Hz, 1H), 3.76–3.66 (m, 2H), 3.43–3.33 (m, 1H), 2.24–2.19 (m, 2H), 2.08–2.03 (m, 2H), 1.55–1.42 (m, 4H), 1.33–1.19 (m, 4H); ^{13}C NMR ($CDCl_3$, 75 MHz, 298 K) δ 145.4, 133.9, 129.7, 128.5, 128.3, 127.6, 119.2, 115.1, 104.7, 70.3, 51.3, 34.2, 31.1; IR (KBr, cm^{-1}): 2934, 1625, 1590; HRMS (FAB): *m/z* calcd for $C_{16}H_{18}BrNO$ [M^+] 319.0572, [M^++2] 321.0553; found 319.0570 [M^+], 321.0558 [M^++2]; calcd for $C_{16}H_{19}BrNO$ [MH^+] 320.0604, [MH^++2] 322.0585; found 320.0607 [MH^+], 322.0673 [MH^++2].

Preparation of compound 2b. To a solution of compound **2a** (474 mg, 1.48 mmol) in dry CH_2Cl_2 (10 mL) was added distilled Et_3N (268 μL, 1.93 mmol). To the mixture cooled to 0 °C with an ice bath was added dropwise a solution of methanesulfonyl chloride (137 μL, 1.78 mmol) in dry CH_2Cl_2 (1 mL). After completion of reaction (30 min, TLC), the reaction was quenched with cold water; the reaction mixture was extracted with CH_2Cl_2 (2 × 100 mL). Organic layer was washed with water and brine, dried over anhydrous Na_2SO_4, and filtered and

concentrated in vacuo. The residue was purified by silica gel column chromatography (CH_2Cl_2/Hexane = 19/1) to give **2b** (384 mg, 65%) as brown solid. ^{1}H NMR ($CDCl_3$, 300 MHz, 298 K) δ 7.8 (d, J = 1.8 Hz, 1H), 7.54–7.39 (m, 3H), 6.85 (dd, J = 8.7, 2.1 Hz, 1H), 4.77–4.68 (m, 1H), 3.48–3.39 (m, 1H), 3.04 (s, 3H), 2.3–2.21 (m, 4H), 1.86–1.72 (m, 2H), 1.48–1.3 (m, 2H).

Preparation of compound 2c. In an oven dried round bottom flask containing a stirrer bar was charged with compound **2b** (384 mg, 1.48 mmol) and anhydrous DMF (20 mL) under nitrogen at room temperature. The flask was placed in an oil bath, and the mixture was stirred and heated at 135 °C for 4 h until most of the starting material disappeared (TLC). After being cooled to room temperature, the reaction mixture was diluted with ethyl acetate (300 mL). The organic layer was washed with water (3 × 50 mL) and brine, dried over anhydrous Na_2SO_4, and concentrated in vacuo. The residue was purified by silica gel column chromatography (Hexane/EtOAc = 19/1) to give **2c** as yellow solid (228 mg, 89%): mp 181–183 °C; ^{1}H NMR ($CDCl_3$, 300 MHz, 298 K) δ 7.83 (d, J = 1.8 Hz, 1H), 7.58 (d, J = 9.0 Hz, 1H), 7.5 (d, J = 8.7 Hz, 1H), 7.42 (dd, J = 8.7, 1.8 Hz, 1H), 7.21 (dd, J = 9.0, 2.1 Hz, 1H), 7.07 (d, J = 2.1 Hz, 1H), 4.3–4.29 (m, 2H), 1.85–1.82 (m, 4H), 1.49–1.47 (m, 4H); ^{13}C NMR ($CDCl_3$, 75 MHz, 298 K) δ 146.5, 133.5, 129.6, 129.5, 129.4, 128.2, 120.2, 116.3, 110.7, 58.3, 29; IR (KBr, cm^{-1}): 2945, 1621; HRMS: *m/z* calcd for $C_{16}H_{16}BrN$ [M^+] 301.0466, [M^++2] 303.0447; found 301.0462 [M^+], 303.0433 [M^++2]; calcd for $C_{16}H_{17}BrN$ [MH^+] 302.0499, [MH^++2] 304.0479; found 302.0511 [MH^+], 304.0515 [MH^++2].

Preparation of compound 2. An oven-dried, two-neck round-bottom flask containing a stirrer bar was charged with compound **2c** (184 mg, 0.61 mmol), $Pd(OAc)_2$ (6.8 mg, 0.03 mmol), diphenyl phosphinopropane (DPPP) (25.2 mg, 0.06 mmol) and ethylene glycol (1.5 mL) under nitrogen at room temperature. Followed by three times degassing, ethylene glycol vinyl ether (279 μL, 1.53 mmol) and distilled Et_3N (255 μL, 1.83 mmol) were sequentially injected to the flask. The flask was placed in an oil bath, and it was stirred at 145 °C for 5 h. After being cooled to room temperature, the mixture was treated with 6 N HCl (4 mL) and heated at 60 °C for 4 h. The reaction mixture was diluted with ethyl acetate (100 mL), and washed sequentially with water (50 mL), 5% aqueous $NaHCO_3$ solution (50 mL), and brine. The organic layer was dried over anhydrous Na_2SO_4, filtered and concentrated in vacuo. The crude product was then purified by silica gel column chromatography (Hexane/EtOAc = 19/1) to give **2** (100 mg, 62%) as yellow solid, which was further purified by crystallization from (Hexane/CH_2Cl_2 = 30/1) (32 mg, 20%): mp 118–120 °C; ^{1}H NMR ($CDCl_3$, 300 MHz, 298 K) δ 8.32 (d, J = 1.5 Hz, 1H), 7.93 (dd, J = 8.7, 1.8 Hz, 1H), 7.78 (d, J = 9.0 Hz, 1H), 7.64 (d, J = 8.7 Hz, 1H), 7.24 (dd, J = 8.7, 2.1 Hz, 1H), 7.11 (d, J = 2.4 Hz, 1H), 4.37–4.34 (m, 2H), 2.67 (s, 3H), 1.87–1.84 (m, 4H), 1.54–1.5 (m, 4H); ^{13}C NMR ($CDCl_3$, 75 MHz, 298 K) δ 198.0, 148.6, 137.7, 131.9, 131.0, 130.4, 127.0, 126.7, 124.7, 119.8, 110.3, 58.3, 29.0, 26.7; IR (KBr, cm^{-1}): 1670; HRMS: *m/z* calcd for $C_{18}H_{19}NO$ [M^+] 265.1467, $C_{18}H_{20}NO$ [MH^+] 266.1545; found 265.1467 [M^+], 266.1547 [MH^+].

Scheme S2.

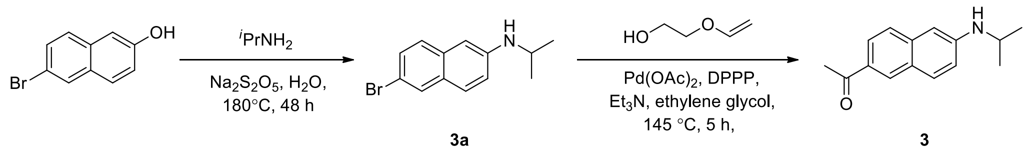

Preparation of compound 3a. 6-Bromo-2-naphthol (1.0 g, 4.50 mmol), sodium metabisulfite (1.3 g, 6.80 mmol) were taken in a sealed tube. Then 10 mL of distilled water followed by 4 mL of isopropyl amine were

added to it. The mixture was heated to 180 °C for 48 h. After being cooled to room temperature, the reaction mixture was diluted with ethyl acetate (300 mL); the organic layer was washed sequentially with water (80 mL), 5% aqueous $NaHCO_3$ solution (50 mL), and brine. The organic layer was dried over anhydrous Na_2SO_4, filtered and concentrated in vacuo, and the residue was purified by silica gel column chromatography (Hexane/EtOAc = 19/1) to give **3a** as yellow solid (1.07 g, 68%): mp 56–58 °C; ^1H NMR ($CDCl_3$, 300 MHz, 298 K) δ 7.79 (d, J = 1.8 Hz, 1H), 7.52 (d, J = 9.0 Hz, 1H), 7.48–7.45 (m, 1H), 7.42–7.38 (m, 1H), 6.86–6.82 (m, 1H), 6.74 (d, J = 2.1 Hz, 1H), 3.79–3.70 (m, 2H), 1.28 (d, J = 6.3 Hz, 6H). ^{13}C NMR ($CDCl_3$, 75 MHz, 298 K) δ 145.6, 134.0, 129.7, 129.6, 128.4, 128.2, 127.7, 119.3, 44.4, 23.0; IR (KBr, cm^{-1}): 2966, 1627, 1517.

Preparation of compound 3. An oven-dried, two-neck round-bottom flask containing a stirrer bar was charged with aryl halide **3a** (550 mg, 2.1 mmol), $Pd(OAc)_2$ (23 mg, 0.11 mmol), diphenyl phosphinopropane (DPPP) (86 mg, 0.22 mmol) and ethylene glycol (3 mL) under nitrogen at room temperature. Followed by three times degassing, ethylene glycol vinyl ether (1.14 mL, 6.2 mmol) and distilled Et_3N (723 μL, 5.2 mmol) were sequentially injected to it. The flask was placed in an oil bath, and the mixture was stirred and heated at 145 °C for 5 h. After being cooled to room temperature, the mixture was treated with 6 N HCl (5 mL) and then heated at 60 °C for 4 h. Then the reaction mixture was diluted with ethyl acetate (100 mL), and washed sequentially with water (50 mL), 5% aqueous $NaHCO_3$ solution (50 mL), and brine. The organic layer was dried over anhydrous Na_2SO_4, filtered and concentrated in vacuo. The crude product was then purified by silica gel column chromatography (Hexane/EtOAc = 19/1) to give **3** (322 mg, 68%) as yellow solid, which was further purified by crystallization (Hexane/CH_2Cl_2 = 24/1) (134 mg, 28%): mp 112–114 °C; ^1H NMR ($CDCl_3$, 300 MHz, 298 K) δ 8.26 (d, J = 9.3 Hz, 1H), 7.91 (dd, J = 8.7, 2.1 Hz, 1H), 7.71 (d, J = 9.0 Hz, 1H), 7.56 (d, J = 8.7 Hz, 1H), 6.86 (dd, J = 9.0, 2.4 Hz, 1H), 6.76 (d, J = 2.1 Hz, 1H), 3.94 (s, 1H), 3.83–3.75 (m, 1H), 2.66 (s, 3H), 1.3 (d, J = 6.3 Hz, 6H); ^{13}C NMR ($CDCl_3$, 75 MHz, 298 K) δ 197.9, 147.6, 138.3, 131.0, 130.8, 130.6, 126.0, 125.8, 124.9, 119.0, 104.0, 44.2, 26.6, 22.9; IR (KBr, cm^{-1}): 1665; HRMS: m/z calcd for $C_{15}H_{17}NO$ [M^+] 227.1310, $C_{15}H_{18}NO$ [MH^+] 228.1388; found 227.1312 [M^+], 228.1390 [$M+H^+$].

Scheme S3.

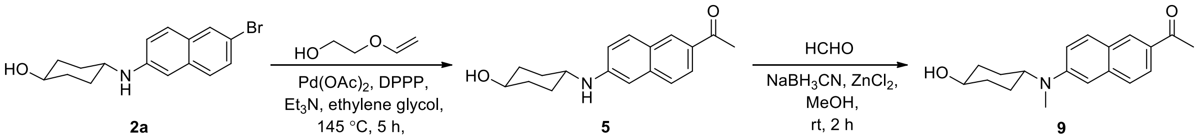

Preparation of compound 5. In an oven-dried, two-neck round-bottom flask containing a stirrer bar was charged with compound **2a** (320 mg, 1.0 mmol), $Pd(OAc)_2$ (11.2 mg, 0.055 mmol), diphenylphosphinopropane (DPPP) (45.39 mg, 0.11 mmol) and ethylene glycol (2 mL) under nitrogen at room temperature. Following degassing three times, ethylene glycol vinyl ether (456 μL, 2.5 mmol) and distilled Et_3N (417 μL, 3.0 mmol) were sequentially injected to it. The flask was placed in an oil bath, and the mixture was heated at 145 °C for 5 h. After being cooled to room temperature 6 N HCl (2 mL), the reaction mixture was heated at 60 °C for 4 h. Then the reaction mixture was diluted with ethyl acetate (150 mL), and washed sequentially with water (50 mL), 5% aqueous $NaHCO_3$ solution (50 mL), and brine. The organic layer was dried over anhydrous Na_2SO_4, filtered and concentrated in vacuo. The crude product was then purified by silica gel column chromatography (CH_2Cl_2/EtOAc = 1/1) to give **5** (203 mg, 72%) as bright yellow solid, which was further purified twice by crystallization from 7% dichloromethane in hexane (120 mg, 42%): mp 186–188 °C; ^1H NMR (CD_3CN, 300

MHz, 298 K) δ 8.33 (s, 1H), 7.84 (dd, J = 8.7, 1.8 Hz, 1H), 7.73 (d, J = 9.0 Hz, 1H), 7.59 (d, J = 8.7 Hz, 1H), 6.96 (dd, J = 9.0, 2.4 Hz, 1H), 6.83 (d, J = 1.8 Hz, 1H), 4.84 (d, J = 7.8 Hz, 1H), 3.62–3.5 (s, 1H), 3.46–3.34 (m, 1H), 2.71 (d, J = 4.5 Hz, 1H), 2.59 (s, 3H), 2.13–2.09 (m, 4H), 1.46–1.2 (m, 4H); ^{1}H NMR (DMSO-d_6, 500 MHz, 300 K) δ 8.33 (d, J = 1 Hz, 1H), 7.77–7.73 (m, 2H), 7.58 (d, J = 8.5 Hz, 1H), 7.02 (dd, J = 9.0, 2.0 Hz, 1H), 6.76 (d, J = 1.5 Hz, 1H), 6.21 (d, J = 7.5 Hz, 1H), 4.57 (d, J = 4.5 Hz, 1H), 3.49–3.43 (m, 1H), 3.35–3.3 (m, 1H), 2.58 (s, 3H), 2.03–2 (m, 2H), 1.89–1.86 (m, 2H), 1.38–1.31 (m, 2H), 1.27–1.2 (m, 2H); ^{13}C NMR (DMSO-d_6, 125 MHz, 300 K) δ 196.8, 148.3, 138.0, 130.4, 129.4, 125.2, 124.6, 124.0, 119.0, 102.0, 68.4, 50.1, 33.9, 30.1, 26.3; IR (KBr, cm^{-1}): 1669; HRMS: *m/z* calcd for $C_{18}H_{21}NO_2$ [M$^+$] 283.1572, $C_{18}H_{22}NO_2$ [MH$^+$] 284.1651; found 283.1575 [M$^+$], 284.1648 [MH$^+$].

Preparation of compound 9. To a stirred solution of compound **5** (50 mg, 0.176 mmol) in methanol (5 mL) containing 37% aqueous formaldehyde (43 μL, 0.53 mmol) at room temperature was added a solution of sodium cyanoborohydride (11.1 mg, 0.176 mmol) and zinc chloride (12 mg, 0.088 mmol) in methanol (2 mL). After being stirred at room temperature for 2 h, the reaction mixture was treated with 0.1 N NaOH solution (2 mL) and concentrated in vacuo to remove most of the methanol. The aqueous solution was extracted with ethyl acetate (3 × 10 mL) and the combined extracts were washed with water and then with brine, dried over $MgSO_4$, and concentrated in vacuo. The crude product was then purified by silica gel column chromatography (EtOAc/Hexane = 1/1) to give **9** (43 mg, 81%) as bright yellow solid, which was further purified twice by crystallization from 5% dichloromethane in hexane (27 mg, 45%): mp 192–194 °C; ^{1}H NMR ($CDCl_3$, 300 MHz, 298 K) δ 8.31 (s, 1H), 7.90 (dd, J = 8.7, 1.8 Hz, 1H), 7.78 (d, J = 9.3 Hz, 1H), 7.61 (d, J = 9.0 Hz, 1H), 6.19 (dd, J = 9.3, 2.4 Hz, 1H), 6.91 (d, J = 2.1 Hz, 1H), 3.87-3.77 (m, 1H), 3.71-3.66 (m, 1H), 2.91 (s, 3H), 2.67 (s, 3H), 2.17–2.02 (m, 2H), 1.88–1.80 (m, 2H), 1.73–1.37 (m, 4H); ^{13}C NMR ($CDCl_3$, 75 MHz, 300 K) δ 198.0, 150.2, 138.0, 131.1, 131.0, 130.5, 126.4, 124.9, 117.1, 106.3, 70.4, 57.4, 35.1, 31.6, 29.9, 27.9, 26.6; IR (KBr, cm^{-1}): 1672; HRMS: *m/z* calcd for $C_{19}H_{23}NO_2$ [M$^+$] 297.1729, $C_{19}H_{24}NO_2$ [MH$^+$] 298.1761; found 297.1727 [M$^+$], 297.1766 [MH$^+$].

Scheme S4.

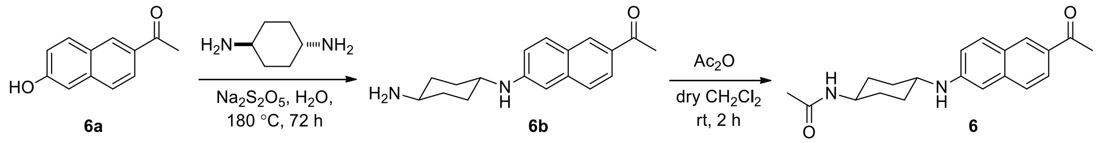

Preparation of compound 6b. Compound **6a** (418 mg, 1.68 mmol), *trans*-1,4-diaminocyclohexane (383 mg, 3.36 mmol), sodium metabisulfite (640 mg, 3.36 mmol) were taken in a sealed tube. Then 10 mL of distilled water was added to it and the mixture was heated to 180 °C for 72 h. After cooling to room temperature, the reaction mixture was filtered through cotton and solvent was removed from the filtrate by vaccum. The residue was purified by silica gel column chromatography (CH_2Cl_2/MeOH = 19/1) to give 600 mg of **6b** (355 mg, 56%) as brown solid, which was crystallized from 25% MeOH in CH_2Cl_2 (139 mg, 22%): mp 198–200 °C; ^{1}H NMR (CD_3OD, 300 MHz, 298 K) δ 8.34 (d, J = 1.5 Hz, 1H), 7.82 (dd, J = 8.7, 1.8 Hz, 1H), 7.71 (d, J = 9.0 Hz, 1H), 7.56 (d, J = 8.7 Hz, 1H), 6.97 (dd, J = 9.0, 2.4 Hz, 1H), 6.80 (d, J = 2.1 Hz, 1H), 3.46–3.41 (m, 1H), 3.08–3.02 (m, 1H), 2.64 (s, 3H), 2.25–2.21 (m, 2H), 2.11–2.07 (m, 2H), 1.53–1.43 (m, 2H), 1.42–1.30 (m, 2H); ^{13}C NMR (CD_3OD, 75 MHz, 298 K) δ 200.6, 149.9, 140.2, 132.2, 132.1, 131.4, 127.1, 126.9, 125.4, 120.3, 104.3, 51.6, 51.2, 32.1, 31.9, 26.5; IR (KBr, cm^{-1}): 3321, 1668, 1550.

Preparation of compound 6. To a solution of compound **6b** (283 mg, 1.0 mmol) in dry CH_2Cl_2 (50 mL) was added acetic anhydride (94 μL, 1.0 mmol) solution in CH_2Cl_2 (10 mL). After stirring at room temperature for 2 h, saturated NH_4Cl solution (10 mL) was added to the reaction mixture. The organic layer was separated and washed with water (10 mL) and dried over $MgSO_4$.The residue was purified by silica gel column chromatography (CH_2Cl_2/MeOH = 19/1) to give compound **6** (299 mg, 92%) as brown solid, which was purified by crystallization from 5% MeOH in CH_2Cl_2 (125 mg, 38%): mp > 250 °C; ^{1}H NMR ($CDCl_3$, 300 MHz, 298 K) δ 8.27 (s, 1H), 7.9 (dd, J = 8.7, 1.8 Hz, 1H), 7.69 (d, J = 8.7 Hz, 1H), 7.57 (d, J = 8.7 Hz, 1H), 6.84 (dd, J = 8.7, 2.4 Hz, 1H), 6.74 (d, J = 1.8 Hz, 1H), 5.36 (d, J = 8.1 Hz, 1H), 3.98 (br, 1H), 3.85–3.83 (m, 1H), 3.39 (br, 1H), 2.66 (s, 3H), 2.25–2.23 (m, 2H), 2.11–2.08 (m, 2H), 1.99 (s, 3H), 1.37–1.30 (m, 4H); ^{13}C NMR ($CDCl_3$, 75 MHz, 298 K) δ 198.0, 169.6, 147.3, 138.2, 131.2, 131.1, 130.6, 126.0, 125.0, 118.9, 104.1, 51.3, 48.2, 32.1, 32.0, 26.6, 23.8; IR (KBr, cm^{-1}): 1653, 1576; HRMS: *m/z* calcd for $C_{20}H_{24}N_2O_2$ [M$^+$] 324.1838, [M$^+$] 325.1869; found 324.1835 [M$^+$], 325.1871 [M$^+$].

Scheme S5.

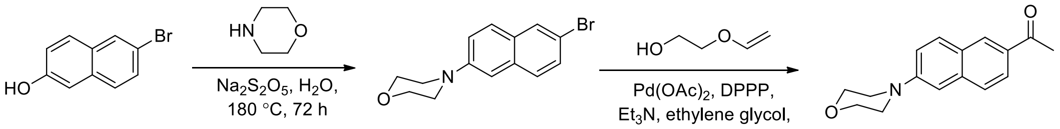

Preparation of compound 8a. 6-Bromo-2-naphthol (1.5 g, 6.72 mmol), morpholine (2.93 g, 33.60 mmol), sodium metabisulfite (2.56 g, 13.45 mmol) were taken in a sealed tube. Then 15 mL of distilled water was added to it and the mixture was heated to 180 °C for 72 h. After cooling to room temperature, the reaction mixture was diluted with ethyl acetate (300 mL), washed with water (80 mL), 5% aqueous $NaHCO_3$ solution (50 mL), and brine. The organic layer was dried over anhydrous Na_2SO_4, filtered and concentrated under vacuum. The residue was purified by silica gel column chromatography (CH_2Cl_2/MeOH = 99/1) to give compound **8a** as brown solid (1.21 g, 62%): mp 158–160 °C; ^{1}H NMR ($CDCl_3$, 300 MHz, 298 K) δ 7.87 (d, J = 1.8 Hz, 1H), 7.64 (d, J = 9.3 Hz, 1H) 7.55 (d, J = 9.0 Hz, 1H), 7.46 (dd, J = 8.7, 2.1 Hz, 1H), 7.24–7.28 (m, 1H), 7.06 (m, 1H), 3.89–3.95 (m, 4H), 3.24–3.30 (m, 4H); ^{13}C NMR ($CDCl_3$, 75 MHz, 298 K) δ 149.6, 133.2, 129.8, 129.7, 128.6, 128.2, 119.87, 117.1, 110.0, 67.1, 49.7; IR (KBr, cm^{-1}): 1617, 1570.

Preparation of compound 8. In an oven-dried two-neck round-bottom flask containing a stirrer bar was charged with compound **8a** (97 mg, 0.33 mmol), Pd $(OAc)_2$ (3.8 mg, 0.017 mmol), diphenyl phosphinopropane (DPPP) (13.8 mg, 0.034 mmol) and ethylene glycol (1 mL) under nitrogen at room temperature. Followed by three times degassing, ethylene glycol vinyl ether (183 μL, 1.0 mmol) and distilled Et_3N (116 μL, 0.84 mmol) were sequentially injected. The flask was placed in an oil bath, and the mixture was heated at 145 °C for 4 hours. After cooling to room temperature 6 N HCl (1.5 mL) was added and the mixture was again heated at 60 °C for 4 more hours. Then the reaction mixture was diluted with ethyl acetate (100 mL), was washed with water (3 × 50 mL) and brine. The organic layer was dried with anhydrous Na_2SO_4, filtered and concentrated in vacuum. The crude product was then purified by silica gel column chromatography (CH_2Cl_2/MeOH = 99/1) to give **8** (54 mg, 64%) as brown solid, which was further purified by crystallization from 1% MeOH in dichloromethane (36 mg, 43%): mp 149–151 °C; ^{1}H NMR ($CDCl_3$, 300 MHz, 298 K) δ 8.34 (s, 1H), 7.97 (d, J = 8.7 Hz, 1H), 7.84 (d, J = 9.3 Hz, 1H), 7.69 (d, J = 8.7 Hz, 1H), 7.26-7.31(m, 1H), 7.1 (s, 1H), 3.91 (t, J = 4.8 Hz, 1H), 3.32 (t, J = 4.6 Hz, 1H), 2.68 (s, 3H); ^{13}C NMR ($CDCl_3$, 75 MHz, 298 K) δ 198.0, 151.2, 137.4, 132.4, 130.9, 130.2, 127.3, 127.1,

124.9, 119.0, 109.2, 67.0, 49.0, 26.7; IR (KBr, cm^{-1}): 1666; HRMS: *m/z* calcd for $C_{16}H_{17}NO_2$ [M^+] 255.1259, $C_{16}H_{18}NO_2$ [MH^+] 256.1292; found 255.1256 [M^+], 256.1279 [MH^+].

Scheme S6.

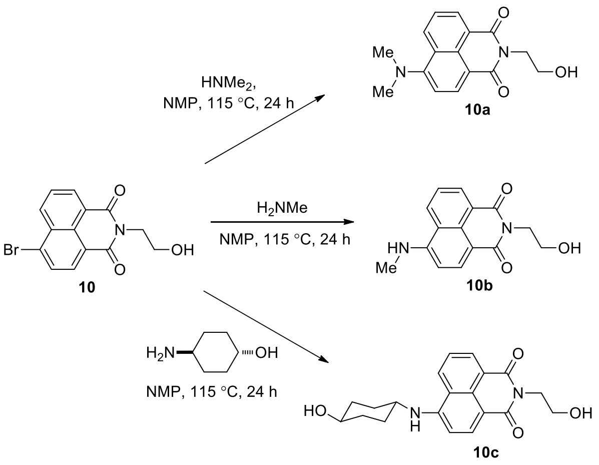

Preparation of compound 10a. Compound **10** (320 mg, 1.0 mmol) and dimethylamine solution (40 wt. % in H_2O, 226 μL, 2.0 mmol), were taken in a sealed tube. Then 2 mL of *N*-methyl pyrolidone (NMP) was added to it and the mixture was heated to 115 °C for 24 h. After cooling to room temperature, the reaction mixture was diluted with ethyl acetate (200 mL), washed with water (50 mL), and brine. The organic layer was dried over anhydrous Na_2SO_4, filtered and concentrated under vacuum. The residue was dissolved in 2mL of $CHCl_3$ and hexane (30 mL) was added gradually to give yellow precipitation, which was filtered and washed with hexane. The solid residue was purified by silica gel column chromatography (EtOAc/Hexane = 1/4) to give compound **10a** as orange solid (200 mg, 70%): mp 129–131 °C; ^{1}H NMR ($CDCl_3$, 300 MHz, 298 K) δ 8.54 (d, *J* = 7.5 Hz, 1H), 8.41 (t, *J* = 8.4 Hz, 2H), 7.61 (t, *J* = 7.5 Hz, 1H), 7.07 (d, *J* = 8.4 Hz, 1H), 4.42 (t, *J* = 5.4 Hz, 2H), 3.93–3.98 (m, 2H), 3.11 (s, 1H), 2.75 (br, 1H); ^{13}C NMR ($CDCl_3$, 75 MHz, 298 K) δ 165.7, 165.2, 157.5, 133.2, 131.8, 131.5, 130.6, 125.3, 125.0, 122.9, 114.6, 113.4, 62.4, 45.0, 42.9.

Preparation of compound 10b. Compound **10** (320 mg, 1.0 mmol, 320.13) and methylamine solution (40 wt. % in H_2O, 156 μL, 2.0 mmol), were taken in a sealed tube. Then 2 mL of *N*-methyl pyrolidone (NMP) was added to it and the mixture was heated to 115 °C for 24 h. After cooling to room temperature, the reaction mixture was diluted with ethyl acetate (200 mL), washed with water (50 mL), and brine. The organic layer was dried over anhydrous Na_2SO_4, filtered and concentrated under vacuum. The residue was dissolved in 2mL of $CHCl_3$ and hexane (20 mL) was added gradually to give yellow precipitation, which was filtered and washed with hexane. The solid residue was purified by silica gel column chromatography (EtOAc/Hexane = 1/1) to give compound **10b** as orange solid (141 mg, 52%): mp > 250 °C; ^{1}H NMR (DMSO-d_6, 300 MHz, 298 K) δ 8.60 (d, *J* = 7.2 Hz, 1H), 8.42 (d, *J* = 7.5 Hz, 1H), 8.28 (d, *J* = 8.4 Hz, 1H), 7.90 (br, s, 1H), 7.68 (t, *J* = 7.5 Hz, 1H), 6.70 (d, *J* = 7.8 Hz, 1H), 4.79 (br, s, 1H), 4.12 (m, 2H), 3.57 (m, 2H), 2.99(s, 3H); ^{13}C NMR (DMSO-d_6, 75 MHz, 298 K) δ 163.9, 163.0, 151.3, 134.2, 130.5, 129.2, 128.2, 124.2, 121.9, 120.0, 107.7, 103.4, 58.0, 41.4, 29.7.

Preparation of compound 10c. Compound **10** (320 mg, 1.0 mmol, 320.13) and *trans*-4-aminocyclohexanol (230 mg, 2.0 mmol), were taken in a sealed tube. Then 2 mL of *N*-methyl pyrolidone (NMP) was added to it and the mixture was heated to 115 °C for 24 h. After cooling to room temperature, the reaction mixture was diluted with ethyl acetate (200 mL), washed with water (50 mL), and brine. The organic layer was dried over anhydrous Na_2SO_4, filtered and concentrated under vacuum. The residue was dissolved in 2mL of $CHCl_3$ and hexane (20 mL) was added gradually to give yellow precipitation, which was filtered and washed with hexane. The solid residue was purified by silica gel column chromatography (EtOAc) to give compound **10c** as orange solid (205 mg, 64%): mp > 250 °C; ^{1}H NMR (DMSO-d_6, 300 MHz, 298 K) δ 8.75 (d, *J* = 8.1 Hz, 1H), 8.41 (d, *J* = 6.9 Hz, 1H), 8.23 (d, *J* = 8.4 Hz, 1H), 7.63 (t, *J* = 7.5 Hz, 1H), 7.29 (d, *J* = 7.5 Hz, 1H), 6.84 (d, *J* = 8.7 Hz, 1H), 4.76 (t,

J = 6.0 Hz, 1H), 4.63 (d, *J* = 4.2 Hz, 1H), 4.09 (t, *J* = 6.9 Hz, 2H), 3.63– 3.51 (m, 3H), 3.48– 3.42 (m, 1H), 1.99 (d, *J* = 12.3 Hz, 2H), 1.89 (d, *J* = 11.1 Hz, 2H), 1.55–1.31 (m, 4H); ^{13}C NMR (DMSO-d_6, 125 MHz, 298 K) δ 164.4, 163.5, 150.3, 034.7, 131.1, 130.1, 129.3, 124.5, 122.4, 120.6, 108.0, 104.7, 68.9, 58.5, 51.5, 41.8, 34.5, 30.2; HRMS: *m/z* calcd for $C_{20}H_{23}N_2O_4$ [MH$^+$] 355.1658; found 355.1659 [MH$^+$].

Scheme S7.

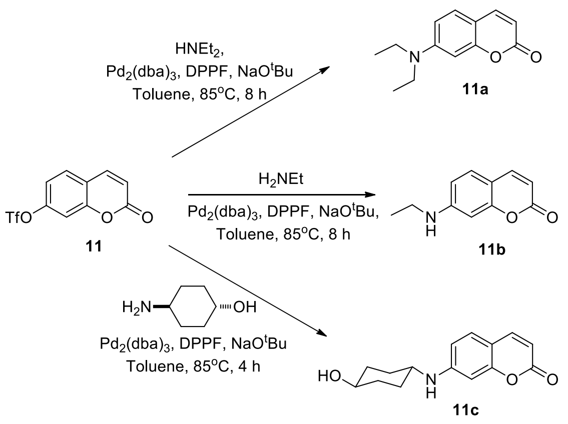

Preparation of compound 11a. In a flame dried tube, $Pd_2(dba)_3$ (31 mg, 0.034 mmol), 1,1′-bis(diphenyl-phosphino)ferrocene (DPPF) (38 mg, 0.068 mmol) and sodium *tert*-butoxide (98 mg, 1.02 mmol) were mixed in 2 mL of toluene under argon atmosphere. The mixture was heated to 85 °C. Then a solution of compound **11** (200 mg, 0.68 mmol) in toluene (2 mL) was added slowly to that mixture. After heating for 30 min, diethylamine (155 µL, 1.02 mmol) was added to the reaction mixture at once. The tube was sealed properly and heated for additional 8 h at 85 °C. Then, the reaction mixture was cooled to room temperature and solvent was evaporated. The residue was purified by column chromatography (EtOAc/Hexane = 1/9) to afford compound **11a** (90 mg, 61%) as pale yellow solid: mp 86–88 °C; ^{1}H NMR ($CDCl_3$, 300 MHz, 298 K) δ 7.75 (d, *J* = 9.3 Hz, 1H), 7.29 (d, *J* = 3.3 Hz, 1H), 6.58 (dd, *J* = 8.7 Hz, 2.4 Hz, 1H), 6.52 (d, *J* = 2.4 Hz, 1H), 6.05 (d, *J* = 9.3 Hz, 1H), 3.41(q, 7.2 Hz, 4H), 1.22 (t, 7.2 Hz, 6H); ^{13}C NMR ($CDCl_3$, 75 MHz, 298 K) δ 162.5, 156.9, 150.9, 143.9, 128.9, 109.4, 108.8, 108.5, 97.7, 45.0, 12.6.

Preparation of compound 11b. In a flame dried tube, $Pd_2(dba)_3$ (31 mg, 0.034 mmol), 1,1′-bis(diphenylphosphino)ferrocene (DPPF) (38 mg, 0.068 mmol) and sodium *tert*-butoxide (98 mg, 1.02 mmol) were mixed in 2 mL of toluene under argon atmosphere. The mixture was heated to 85 °C. Then a solution of compound **11** (200 mg, 0.68 mmol) in toluene (2 mL) was added slowly to that mixture. After heating for 30 min, ethylamine solution (2.0 M in THF, 680 µL, 1.36 mmol) was added to the reaction mixture at once. The tube was sealed properly and heated for additional 8 h at 85 °C. Then, the reaction mixture was cooled to room temperature and solvent was evaporated. The residue was purified by column chromatography (EtOAc/Hexane = 1:4) to afford compound **11b** (54 mg, 42%) as pale yellow solid: mp 143–145 °C; ^{1}H NMR ($CDCl_3$, 300 MHz, 298 K) δ 7.52 (d, *J* = 9.6 Hz, 1H), 7.19 (d, *J* = 8.4 Hz, 1H), 6.49–6.43 (m, 2H), 6.06 (d, *J* = 9.3 Hz, 1H), 4.17 (br, 1H), 3.26– 3.17 (m, 2H), 1.25 (t, *J* = 5.7 Hz, 4H); ^{13}C NMR ($CDCl_3$, 75 MHz, 298 K) δ 162.3, 156.9, 151.9, 144.0, 128.9, 110.7, 110.1, 109.8, 98.0, 38.3, 14.7.

Preparation of compound 11c. In a flame dried tube, $Pd_2(dba)_3$ (31 mg, 0.034 mmol), 1,1′-bis(diphenylphosphino)ferrocene (DPPF) (38 mg, 0.068 mmol) and sodium *tert*-butoxide (98 mg, 1.02 mmol) were mixed in 2 mL of toluene under argon atmosphere. The mixture was heated to 85 °C. Then a solution of compound **11** (200 mg, 0.68 mmol) in toluene (2 mL) was added slowly to that mixture. After heating for 30 min, *trans*-4-aminocyclohexanol (117 mg, 1.02 mmol) was added to the reaction mixture at once. The tube was sealed properly and heated for additional 4 h at 85 °C. Then, the reaction mixture was cooled to room temperature and solvent was evaporated. The residue was purified by column chromatography (EtOAc/Hexane = 1:1) to afford compound **11c** (85 mg, 48%) as pale yellow solid: mp 207–209 °C; ^{1}H NMR (CD_3OD, 300

MHz, 298 K) δ 7.70 (d, J = 9 Hz, 1H), 7.26 (d, J = 8.7 Hz, 1H), 6.56 (dd, J = 8.4 Hz, 2.1 Hz, 1H), 6.41 (d, J = 2.1 Hz, 1H), 5.95 (d, J = 9.3 Hz, 1H), 3.64– 3.54(m, 1H), 3.36– 3.27(m, 1H), 2.10–1.97 (m, 4H), 1.50–1.23 (m, 4H); ^{13}C NMR (CD_3OD, 75 MHz, 298 K) δ 164.8, 158.2, 153.7, 146.5, 130.3, 112.3, 110.1, 108.6, 98.0, 70.7, 51.9, 34.7, 31.5.

Scheme S8.

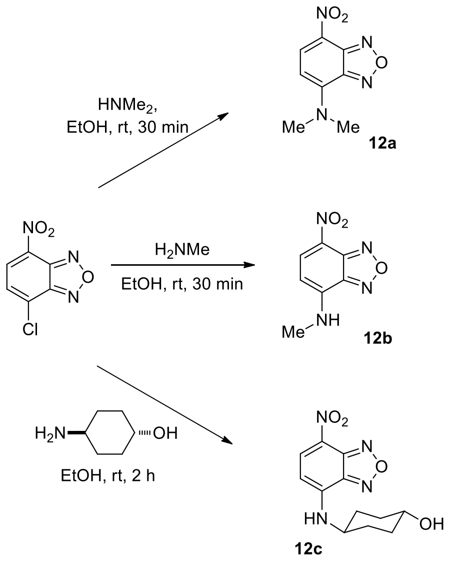

Preparation of compound 12a. NBD chloride (200 mg, 1.0 mmol), dimethylamine solution (40 wt. % in H_2O, 113 µL, 1.0 mmol) were taken in a reaction flusk. Then 5 mL of ethanol was added to it and the mixture was stirred at room temparature for 30 min. After the completion of reaction, the reaction mixture was diluted with ethyl acetate (200 mL), washed with water (50 mL), and brine. The organic layer was dried over anhydrous Na_2SO_4, filtered and concentrated under vacuum. The residue was purified by silica gel column chromatography (Hexane/EtOAc = 1/9) to give compound **12a** as orange solid (150 mg, 72%): mp 212–214 °C; ^{1}H NMR ($CDCl_3$, 300 MHz, 298 K) δ 8.42 (d, J = 9.0 Hz, 1H), 6.07 (d, J = 9.0 Hz, 1H), 3.63 (s, 6H); ^{13}C NMR ($CDCl_3$, 75 MHz, 298 K) δ 146.3, 145.1, 135.5, 101.2, 43.7.

Preparation of compound 12b. NBD chloride (200 mg, 1.0 mmol), methylamine solution (40 wt. % in H_2O, 78 µL, 1.0 mmol) were taken in a reaction flusk. Then 5 mL of ethanol was added to it and the mixture was stirred at room temparature for 30 min. After the completion of reaction, the reaction mixture was diluted with ethyl acetate (200 mL), washed with water (50 mL), and brine. The organic layer was dried over anhydrous Na_2SO_4, filtered and concentrated under vacuum. The residue was purified by silica gel column chromatography (Hexane/EtOAc = 1/4) to give compound **12b** as dark red solid (126 mg, 65%): mp 228–230 °C (decomposed); ^{1}H NMR (DMSO-d_6, 300 MHz, 298 K) δ 9.49 (br, 1H), 8.51 (d, J = 9.0 Hz, 1H), 6.29 (d, J = 9.0 Hz, 1H), 3.07 (s, 3H); ^{13}C NMR (DMSO-d_6, 75 MHz, 298 K) δ 145.8, 144.3, 144.1, 137.9, 120.8, 99.8, 99.0, 30.2.

Preparation of compound 12c. NBD chloride (200 mg, 1.0 mmol), *trans*-4-aminocyclohexanol (116 mg, 1.0 mmol) were taken in a reaction flusk. Then 5 mL of ethanol was added to it and the mixture was stirred at room temparature for 2 h. After the completion of reaction, the reaction mixture was diluted with ethyl acetate (200 mL), washed with water (50 mL), and brine. The organic layer was dried over anhydrous Na_2SO_4, filtered and concentrated under vacuum. The residue was purified by silica gel column chromatography (Hexane/EtOAc = 1/1) to give compound **12c** as red solid (210 mg, 75%): mp: 192–194 °C; ^{1}H NMR (DMSO-d_6, 300 MHz, 298 K) δ 9.27 (br, 1H), 8.46 (d, J = 9.0 Hz, 1H), 6.47 (d, J = 9.0 Hz, 1H), 4.65 (d, J = 4.5 Hz, 1H), 3.73 (br, 1H), 3.4–3.43 (m, 1H), 1.87–1.95 (m, 4H), 1.52–1.68 (m, 2H), 1.23–1.39 (m, 2H); ^{13}C NMR (DMSO-d_6, 125 MHz, 298 K) δ 144.9, 138.4, 120.9, 99.9, 68.5, 52.6, 34.2, 29.7; HRMS: m/z calcd for $C_{12}H_{15}N_4O_4$ [MH^+] 279.1093; found 279.1095 [MH^+].

Scheme S9.

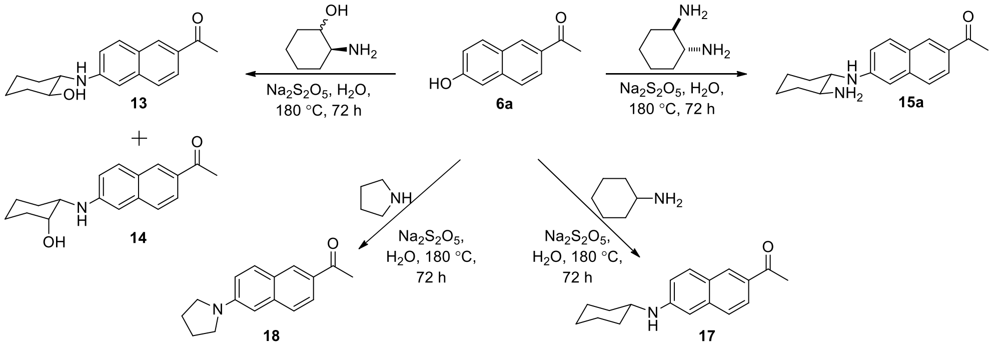

Preparation of compound 13, 14, 15a, 17, 18. All of these compounds were synthesised from compound **6a** (1 equiv.) following the same procedure (Bucherer reaction) as mentioned above for the synthesis of compound **6b,** by using the corresponding amine. 2-Aminocyclohexanol (a mixture of *cis* and *trans* isomers, 5 equiv.) was used for the synthesis of compound **13** and **14** which were later separated by column chromatography. *trans*-1,2-Diaminocyclohexane (2 equiv.), cyclohexylamine (5 equiv.) or pyrrolidine (5 equiv.) were used respectively for the synthesis of compound **15a**, **17** and **18**, respectively.

Compound **13** (R_f = 0.36, EtOAc/Hexane = 1/3): ^{1}H NMR ($CDCl_3$, 300 MHz, 298 K) δ 8.18 (s, 1H), 7.83 (dd, *J* = 8.7, 1.8 Hz, 1H), 7.58 (d, *J* = 9.0 Hz, 1H), 7.48 (d, *J* = 8.7 Hz, 1H), 6.84 (dd, *J* = 8.7, 2.1 Hz, 1H), 6.72 (d, *J* = 1.8 Hz, 1H), 4.61 (br, s, 1H), 4.13 (br, s, 1H), 3.53–3.50 (m, 1H), 2.92 (d, *J* = 3.3 Hz, 1H), 2.59 (s, 3H), 1.90–1.86 (m, 1H), 1.78–1.58 (m, 5H), 1.47–1.37 (m, 2H); ^{13}C NMR ($CDCl_3$, 75 MHz, 298 K) δ 198.3, 147.5, 138.1, 130.9, 130.6, 130.5, 125.8, 125.7, 124.6, 119.0, 104.1, 67.7, 54.3, 31.8, 26.7, 26.4, 24.1, 20.0.

Compound **14** (R_f = 0.28, EtOAc/Hexane = 1/3): ^{1}H NMR ($CDCl_3$, 300 MHz, 298 K) δ 8.26 (s, 1H), 7.89 (dd, *J* = 8.7, 1.8 Hz, 1H), 7.69 (d, *J* = 8.7 Hz, 1H), 7.57 (d, *J* = 8.7 Hz, 1H), 6.97-6.92 (m, 2H), 3.99 (br, 1H), 3.50–3.42 (m, 1H), 3.38–3.30 (m, 1H), 2.65 (s, 3H), 2.21–2.13 (m, 2H), 1.83–1.74 (m, 2H), 1.53–1.31 (m, 4H), 1.28–1.13 (m, 1H); ^{13}C NMR ($CDCl_3$, 75 MHz, 298 K) δ 198.1, 148.2, 138.0, 131.3, 131.1, 130.5, 126.4, 126.3, 125.0, 119.2, 105.4, 59.7, 33.7, 31.7, 26.6, 25.0, 24.4.

Compound **15a**: ^{1}H NMR ($CDCl_3$, 300 MHz, 298 K) δ 8.19 (s, 1H), 7.83 (d, *J* = 8.7 Hz, 1H), 7.59 (d, *J* = 8.7 Hz, 1H), 7.50 (d, *J* = 8.7 Hz, 1H), 6.92 (d, *J* = 8.1 Hz, 1H), 6.80 (s, 1H), 4.38 (br, s, 1H), 3.87–3.43 (br, 3H), 3.23–3.08 (m, 1H), 2.59 (s, 3H), 2.15–2.11 (m, 1H), 2.01–1.94 (m, 1H), 1.72–1.68 (m, 2H), 1.39–1.10 (m, 3H), 1.09–0.89 (m, 1H).

Compound **17**: ^{1}H NMR ($CDCl_3$, 300 MHz, 298 K) δ 8.27 (d, *J* = 1.5 Hz, 1H), 7.89 (dd, *J* = 8.7, 1.8 Hz, 1H), 7.69 (d, *J* = 8.7 Hz, 1H), 7.57 (d, *J* = 8.7 Hz, 1H), 6.85 (dd, *J* = 9.0, 2.4 Hz, 1H), 6.76 (d, *J* = 2.1 Hz, 1H), 4.00 (br, s, 1H), 3.46–3.39 (m, 1H), 2.66 (s, 3H), 2.15–2.11 (m, 2H), 1.84–1.78 (m, 2H), 1.84–1.78 (m, 2H), 1.69-1.61 (m, 1H), 1.48–1.38 (m, 2H), 1.32–1.21 (m, 3H); ^{13}C NMR ($CDCl_3$, 75 MHz, 298 K) δ 197.9, 147.5, 138.3, 131.1, 130.8, 130.6, 126.0, 125.8, 124.9, 118.9, 103.9, 51.6, 33.3, 26.6, 26.0, 25.1.

Compound **18**: ^{1}H NMR ($CDCl_3$, 300 MHz, 298 K) δ 8.29 (d, *J* = 0.9 Hz, 1H), 7.89 (dd, *J* = 8.7, 1.8 Hz, 1H), 7.74 (d, *J* = 9.0 Hz, 1H), 7.57 (d, *J* = 8.7 Hz, 1H), 6.96 (dd, *J* = 9.0, 2.4 Hz, 1H), 6.69 (d, *J* = 2.1 Hz, 1H), 3.38 (t, *J* = 6.6 Hz, 4H), 2.65 (s, 3H), 2.09–2.00 (m, 4H); ^{13}C NMR ($CDCl_3$, 75 MHz, 298 K) δ 197.8, 147.8, 138.1, 131.0, 130.8, 130.2, 125.9, 124.8, 124.7, 116.4, 104.4, 47.8, 26.5, 25.6.

Scheme S10.

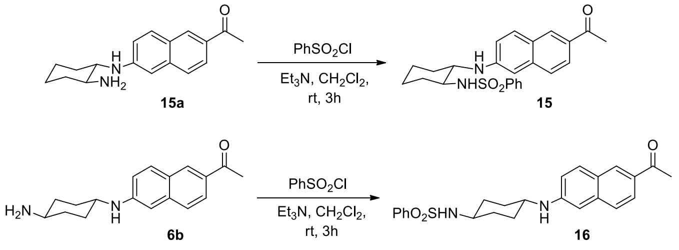

Preparation of compounds 15 and 16. Compound **15** and **16** were prepared from the corresponding amine, **15a** or **6b**, after reaction with benzenesulfonyl chloride (1 equiv.) in the presence of Et_3N (1 equiv.) in dichloromethane at room temparature for 3 h.

Compound **15**: ^{1}H NMR ($CDCl_3$, 300 MHz, 298 K) δ 8.27 (d, *J* = 1.2 Hz, 1H), 7.93–7.85 (m, 3H), 7.66 (d, *J* = 8.7 Hz, 1H), 7.60–7.54 (m, 2H), 7.50–7.45 (m, 2H), 6.73 (dd, *J* = 8.7, 2.1 Hz, 1H), 6.67 (d, *J* = 2.1 Hz, 1H), 4.87 (d, *J* = 6.6 Hz, 1H), 4.23 (d, *J* = 7.2 Hz, 1H), 3.25–3.18 (m, 1H), 3.15–3.08 (m, 1H), 2.66 (s, 3H), 2.33–2.29 (m, 1H), 1.93–1.89 (m, 1H), 1.75–1.65 (m, 2H), 1.39–1.23 (m, 3H), 1.19–1.12 (m, 1H); ^{13}C NMR ($CDCl_3$, 75 MHz, 298 K) δ 198.1, 147.4, 140.9, 138.1, 132.9, 131.1, 131.1, 130.6, 129.4, 127.1, 126.2, 126.1, 124.9, 119.1, 104.0, 57.1, 56.7, 33.4, 32.1, 26.6, 24.8, 24.2.

Compound **16**: ^{1}H NMR ($CDCl_3$, 300 MHz, 298 K) δ 8.26 (d, *J* = 1.2 Hz, 1H), 7.94-7.89 (m, 3H), 7.67 (d, *J* = 8.7 Hz, 1H), 7.60–7.50 (m, 4H), 6.80 (dd, *J* = 9.0, 2.4 Hz, 1H), 6.69 (d, *J* = 2.1 Hz, 1H), 4.79–4.77 (m, 1H), 3.89 (br, s, 1H), 3.33–3.18 (m, 2H), 2.65 (s, 3H), 2.17–2.13 (m, 2H), 1.97–1.93 (m, 2H), 1.44–1.31 (m, 2H), 1.27–1.14 (m, 2H); ^{13}C NMR ($CDCl_3$, 75 MHz, 298 K) δ 198.0, 147.1, 141.4, 138.1, 131.2, 131.1, 129.4, 127.1, 126.0, 125.0, 118.8, 104.1, 52.6, 50.7, 32.8, 31.7, 26.6.

Scheme S11.

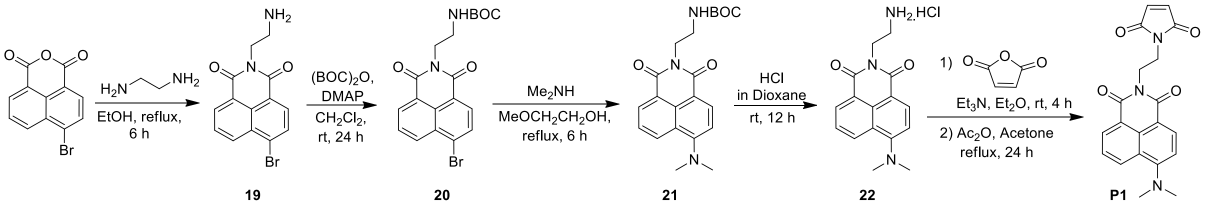

Preparation of compound 20. Ethylenediamine (266 µL, 3.98 mmol) was added to a suspension of 4-bromo-1,8-naphthalic anhydride (1 g, 3.62 mmol) in ethanol (10 mL). The mixture was then refluxed for 6 hours, and then the solvent was evaporated under vacuum to give crude compound **19** which was directly used for the next step. The crude compound **19** and 4-(dimethylamino)pyridine (885 mg, 7.24 mmol) were dissolved in dichloromethane (30 mL), which was then treated slowly with di-*tert*-butyl dicarbonate (1.58 g, 7.24 mmol) at 0 °C. After being stirred at room temparature for 24 h, water (30 mL) was added to the reaction mixture and the organic layer was separated. The aqueous layer was extracted with dichloromethane (2 × 30 mL). The combined organic phase was washed with brine (20 mL) and dried over anhydrous Na_2SO_4, filtered and concentrated under vacuum to give a brown residue. Silica gel column chromatography (EtOAc/Hexane = 1/4) of the brown residue gave pure compound **20** (1.1 g, 72%) as a white solid. ^{1}H NMR ($CDCl_3$, 300 MHz, 298 K) δ 8.62 (dd, *J* = 7.2, 0.9 Hz, 1H), 8.52 (dd, *J* = 8.7, 1.2 Hz, 1H), 8.38 (d, *J* = 7.8 Hz, 1H), 8.00 (d, *J* = 8.1 Hz, 1H), 7.80 (t, *J* = 7.5 Hz, 1H), 5.00 (br, m, 1H), 4.32 (t, *J* = 5.7 Hz, 2H), 3.52–3.53 (m, 2H), 1.27 (s, 9H).

Preparation of compound 21. Compound **20** (200 mg, 0.48 mmol) and a dimethylamine solution (40 wt. % in H_2O, 108 µL, 0.96 mmol) were taken in a sealed tube. Then, 2 mL of 2-methoxyethanol was added to it and the mixture was heated to reflux at 120 °C for 6 h. After being cooled to room temperature, the reaction mixture was diluted with ethyl acetate (50 mL), washed with water (50 mL) and then brine. The organic layer was dried

over anhydrous Na_2SO_4, filtered and concentrated under vacuum. The solid residue was purified by silica gel column chromatography (EtOAc/Hexane = 1/4) to give compound **21** (124 mg, 68%) as an orange solid. ^{1}H NMR ($CDCl_3$, 300 MHz, 298 K) δ 8.34 (d, *J* = 7.2 Hz, 1H), 8.22–8.27 (m, 2H), 7.44 (t, *J* = 7.2 Hz, 1H), 6.90 (d, *J* = 8.4 Hz, 1H), 5.28 (br, m, 1H), 4.19 (t, *J* = 5.4 Hz, 2H), 3.42–3.44 (m, 2H), 2.99 (s, 6H), 1.26 (s, 9H).

Preparation of P1. Compound **21** (100 mg, 0.26 mmol) was dissolved in dichloromethane (5 mL) and cooled to 0 °C, and then treated slowly with 4N HCl in dioxane (1 mL) at 0 °C. After being stirred at room temparature for 12 h, the reaction mixture was concentrated to give compound **22** as pale yellow solid. The solid was dried completely under high-vacum and directly used for the introduction of the maleimide moiety. Compound **22** (83 mg) was dissolved in in Et_2O (10 mL) and then treated with Et_3N (108 µL, 0.78 mmol) at 0 °C, followed by a solution of maleic anhydride (26 mg, 0.26 mmol) slowly in Et_2O (10 mL). The reaction mixture was stirred for 4 h while being allowed to reach room temperature, then it was concentrated to give a residue. The residue was redissolved in acetone (5 mL) and treated with Et_3N (108 µL, 0.78 mmol), and the resulting mixture was heated to reflux for 1 h. Then, acetic anhydride (37 µL, 0.39 mmol) was added to the solution, and it was heated to reflux for additional 24 h. After the solvent was removed under reduced pressure, chromatography of the dark-brown oily residue over silica gel (EtOAc/hexane = 1/1) gave pure probe **P1** (55 g, 58%) as an orange solid. ^{1}H NMR ($CDCl_3$, 300 MHz, 298 K) δ 8.48 (dd, *J* = 7.2, 1.2 Hz, 1H), 8.39–8.42 (m, 2H), 7.59 (t, *J* = 7.2 Hz, 1H), 7.05 (d, *J* = 8.1 Hz, 1H), 6.60 (s, 2H), 4.37–4.41 (m, 2H), 3.95–3.98 (m, 2H), 3.08 (s, 6H); ^{13}C NMR ($CDCl_3$, 75 MHz, 298 K) δ 171.0, 165.0, 164.4, 157.3, 134.3, 132.9, 131.6, 131.3, 130.6, 125.5, 125.0, 122.8, 114.6, 113.4, 44.9, 38.865, 36.5.

Scheme S12.

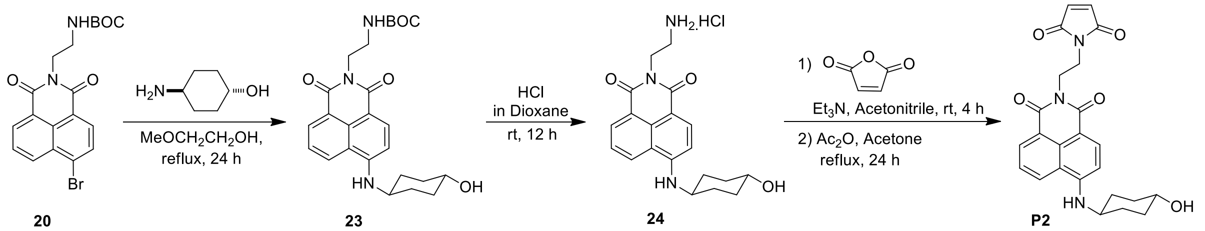

Preparation of compound 23. Compound **20** (200 mg, 0.48 mmol) and *trans*-4-aminocyclohexanol (111 mg, 0.96 mmol) were taken in a sealed tube. Then, 2 mL of 2-methoxyethanol was added to it and the mixture was heated to reflux at 120 °C for 24 h. After being cooled to room temperature, the reaction mixture was diluted with ethyl acetate (100 mL), washed with water (50 mL), and brine. The organic layer was dried over anhydrous Na_2SO_4, filtered and concentrated under vacuum. The solid residue was purified by silica gel column chromatography (EtOAc) to give compound **23** (112 mg, 52%) as an orange solid. ^{1}H NMR ($CDCl_3$, 300 MHz, 298 K) δ 8.42 (d, *J* = 7.2 Hz, 1H), 8.35 (d, *J* = 8.4 Hz, 1H), 8.01 (d, *J* = 8.4 Hz, 1H), 7.44 (t, *J* = 7.8 Hz, 1H), 6.66 (d, *J* = 8.7 Hz, 1H), 5.34 (d, *J* = 7.2, 1H), 5.23 (br, m, 1H), 4.28 (t, *J* = 5.4 Hz, 2H), 3.72–3.76 (m, 1H), 3.50–3.57 (m, 3H), 2.20–2.28 (m, 2H), 2.10–2.13 (m, 2H), 1.42–1.63 (m, 4H), 1.32 (s, 9H); ^{13}C NMR ($CDCl_3$, 75 MHz, 298 K) δ 165.2, 164.6, 156.3, 148.9, 134.8, 131.5, 130.2, 126.3, 124.7, 122.9, 120.3, 109.7, 104.8, 79.3, 69.9, 51.4, 40.4, 39.6, 34.0, 30.6, 28.5.

Preparation of P2. Compound **23** (100 mg, 0.22 mmol) was dissolved in dichloromethane (5 mL) and cooled to 0 °C, and then treated with a solution of 4N HCl in dioxane (1 mL) slowly. After being stirred at room temparature for 12 h, the mixture was concentrated to give compound **24** as a yellow solid. The solid was dried

completely under high-vacum and directly used for the introduction of the maleimide moiety. A solutino of compound **24** (86 mg) in acetonitrile (10 mL) was treated with Et_3N (91 µL, 0.66 mmol) at 0 °C, followed by a solution of maleic anhydride (22 mg, 0.22 mmol) in Et_2O (10 mL) slowly, and the resulting mixture was stirred for 4 h while being allowed to reach room temperature. The reaction mixture was concentrated to give a reside, which was dissolved in acetone (10 mL) and treated with Et_3N (91 µL, 0.66 mmol). The resulting mixture was heated to reflux for 4 h. Then, acetic anhydride (31 µL, 0.33 mmol) was added to the mixture, and the resulting solution was further heated to reflux for 24 h. The reaction mixture was concentrated under reduced pressure, and the dark-brown oily residue was purified by column chromatography over silica gel (EtOAc) to afford pure probe **P2** (31 mg, 32%) as an orange solid. ^{1}H NMR ($CDCl_3$, 300 MHz, 298 K) δ 8.50 (d, J = 7.2 Hz, 1H), 8.38 (d, J = 8.4 Hz, 1H), 8.01 (d, J = 8.1 Hz, 1H), 7.53 (t, J = 7.5 Hz, 1H), 6.70 (d, J = 8.4 Hz, 1H), 6.61 (s, 2H), 5.13 (d, J = 6.3, 1H), 4.38 (t, J = 5.1 Hz, 2H), 3.96 (t, J = 5.1 Hz, 2H), 3.73–3.80 (m, 1H), 3.61 (m, 1H), 2.26–2.30 (m, 2H), 2.10–2.14 (m, 2H), 1.37–1.56 (m, 4H); ^{13}C NMR ($CDCl_3$, 75 MHz, 298 K) δ 171.0, 165.1, 164.4, 148.7, 134.7, 134.3, 131.5, 130.4, 126.1, 124.8, 123.0, 120.5, 110.0, 104.9, 69.9, 51.3, 38.8, 36.6, 34.0, 30.7.

References

1. Fischer, A.; Cremer, C.; Stelzer, E. H. *Applied Optics,* 1995, **34**, 1989–2003.
2. Rumi, M.; Ehrlich, J. E.; Heikal, A. A.; Perry, J. W.; Barlow, S.; Hu, Z.; McCord-Maughon, D.; Parker, T. C.; Ro1ckel, H.; Thayumanavan, S.; Marder, S. R.; Beljonne, D.; Brédas, J.-L. *J. Am. Chem. Soc.*, 2000, **122**, 9500–9510.
3. Manoj, P.; Min, C.-K.; Aravindakumar, C. T.; Joo, T. *Chem. Phys.*, 2008, **352**, 333–338.
4. Zhao, Y.; Truhlar, D. G. *Acc. Chem. Res.*, 2008, **41**, 157–167.
5. Reed, A. E.; Weinstock, R. B.; Weinhold, F. *J. Chem. Phys.*, 1985, **83**, 735–746.
6. Hornak, V.; Abel, R.; Okur, A.; Strockbine, B.; Roitberg, A.; Simmerling, C. *Proteins*, 2006, **65**, 712–725.
7. Berendsen, H. J. C.; Postma, J. P. M.; van Gunsteren, W. F.; DiNola, A.; Haak, J. R. *J. Chem. Phys.*, 1984, **81**, 3684–3690.
8. Nosé, S. *J. Chem. Phys.*, 1984, **81**, 511–519.
9. Parrinello, M.; Rahman, A. *J. Appl. Phys.*, 1981, **52**, 7182–7190.
10. Rhee, Y. M.; Head-Gordon, M. *J. Phys. Chem. A*, 2007, **111**, 5314–5326.
11. Rhee, Y. M.; Casanova, D.; Head-Gordon, M. *J. Phys. Chem. A*, 2009, **113**, 10564–10576.
12. Hess, B.; Bekker, H.; Berendsen, H. J. C.; Fraajie, J. G. E. M. *J. Comput. Chem.*, 1997, **18**, 1463–1472.
13. Shao, Y. *et al. Phys. Chem. Chem. Phys.*, 2006, **8**, 3172–3191.
14. Kuo, I.-F. W.; Mundy, C. J. *Science*, 2004, **303**, 658–660.
15. Lee, H.-S.; Tucker, M. E. *J. Chem. Phys.*, 2006, **125**, 154507–14.
16. Rao, A. S.; Singha, S.; Choi, W.; Ahn, K. H. *Org. Biomol. Chem.*, 2012, **10**, 8410–8417.
17. Yin, L.; He, C.; Huang, C.; Zhu, W.; Wang, X.; Xu, Y.; Qian, X. *Chem. Commun.*, 2012, **48**, 4486–4488.
18. Yu, C.; Li, X.; Zeng, F.; Zheng, F.; Wu, S. *Chem. Commun.*, 2013, **49**, 403–405.
19. Starčević, Š.; Brožič, P.; Turk, S.; Cesar, J.; Rižner, T. L.; Gobec, S. *J. Med. Chem.*, 2011, **54**, 248–261.

^{1}H NMR and ^{13}C NMR spectra

^{1}H NMR ($CDCl_3$, 300 MHz, 298 K) of Compound **2a**

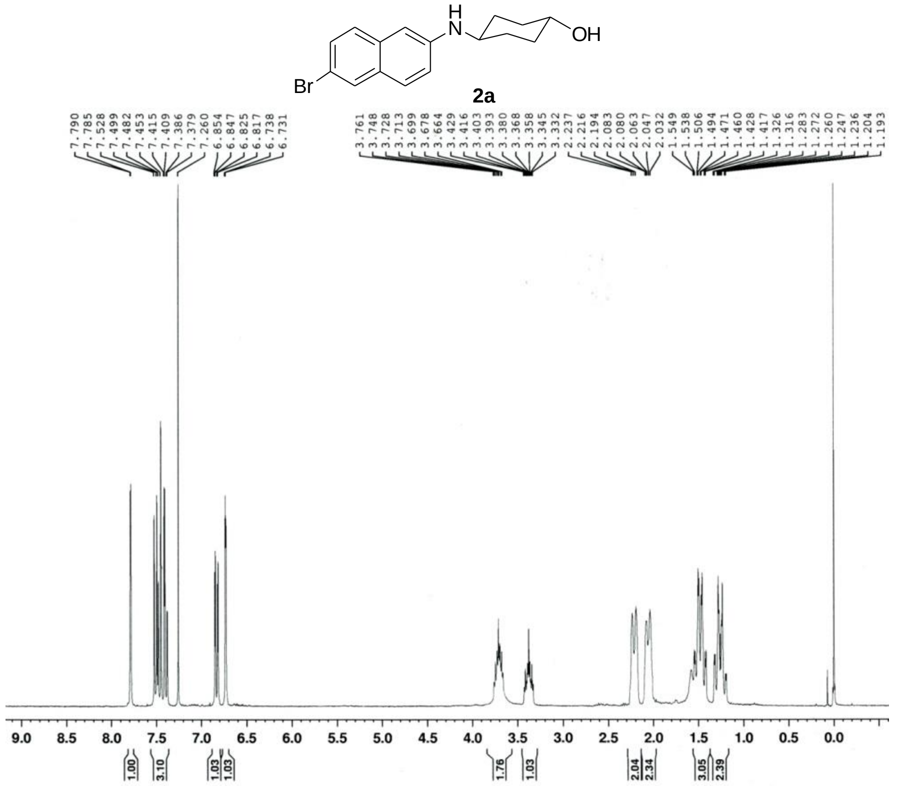

^{13}C NMR ($CDCl_3$, 75 MHz, 298 K) of Compound **2a**

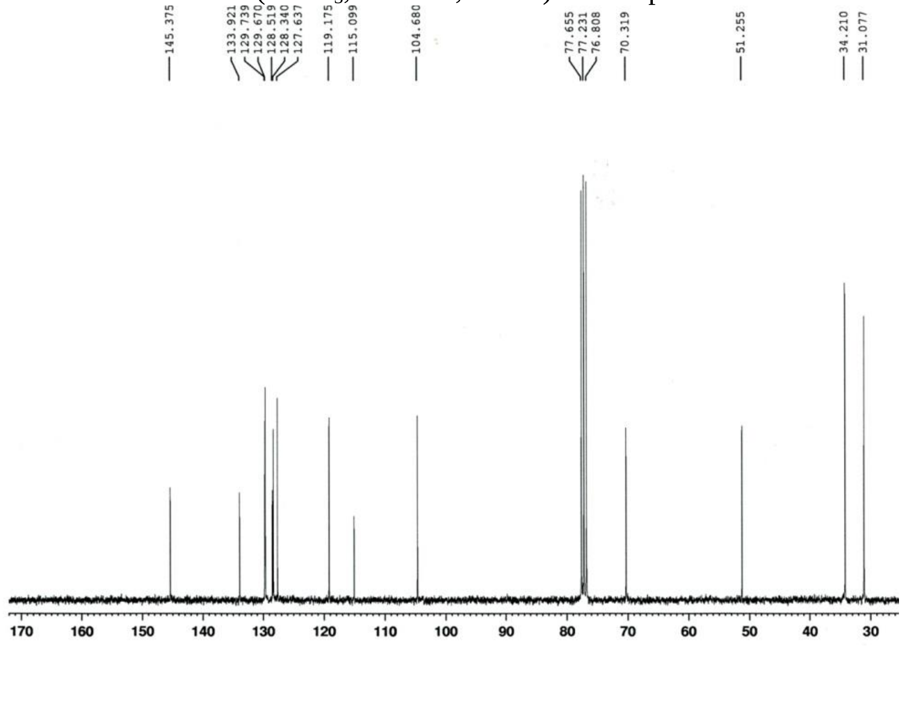

^{1}H NMR ($CDCl_3$, 300 MHz, 298 K) of Compound **2b**

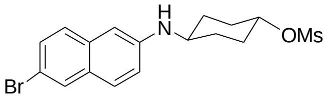

2b

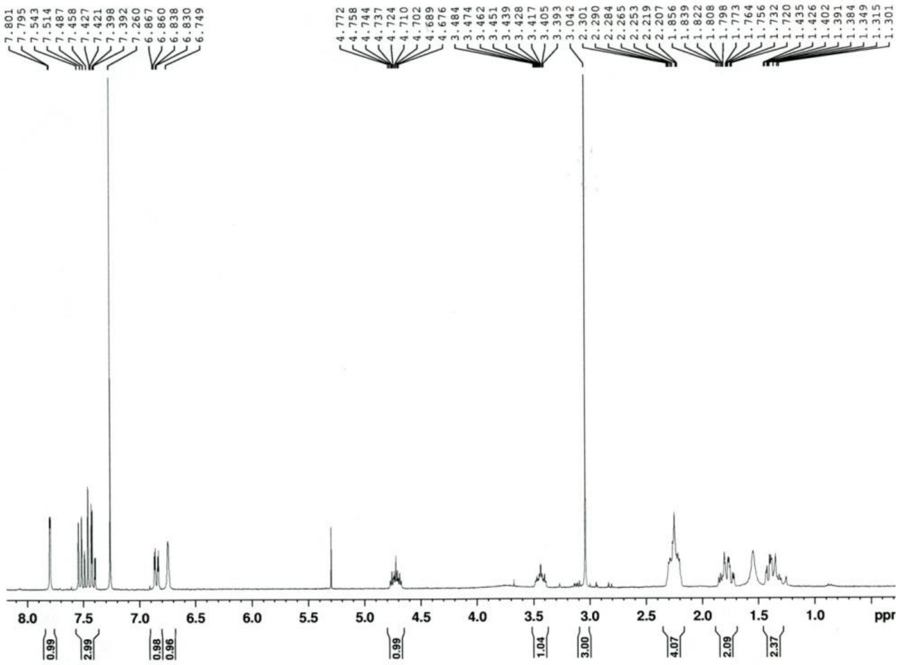

^{1}H NMR ($CDCl_3$, 300 MHz, 298 K) of Compound **2c**

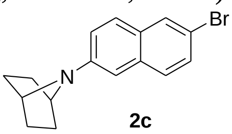

2c

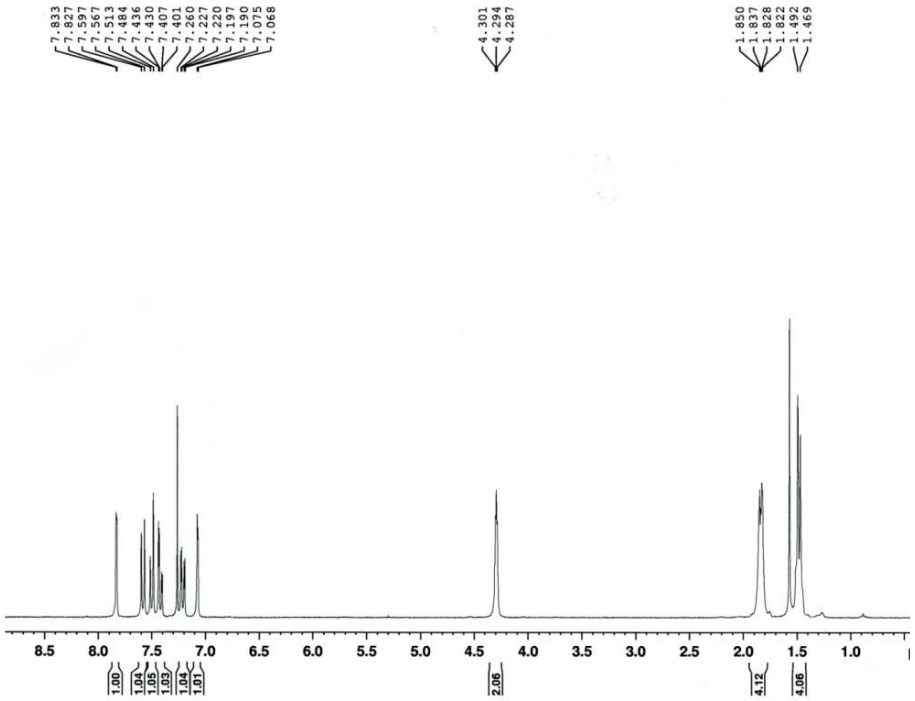

^{13}C NMR ($CDCl_3$, 75 MHz, 298 K) of Compound **2c**

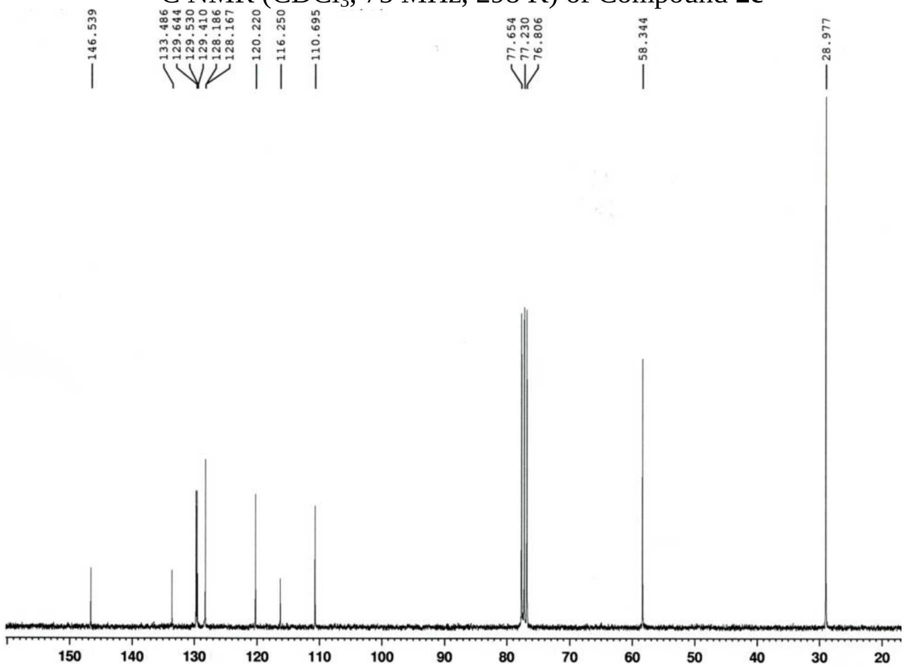

^{1}H NMR ($CDCl_3$, 300 MHz, 298 K) of Compound **2**

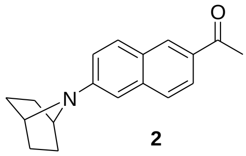

2

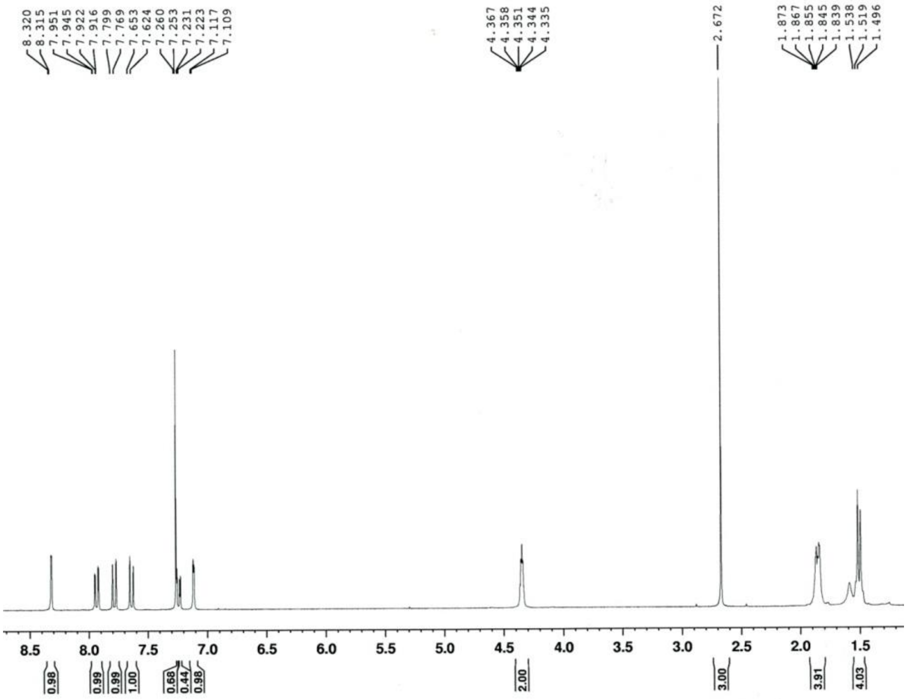

^{13}C NMR ($CDCl_3$, 75 MHz, 298 K) of Compound **2**

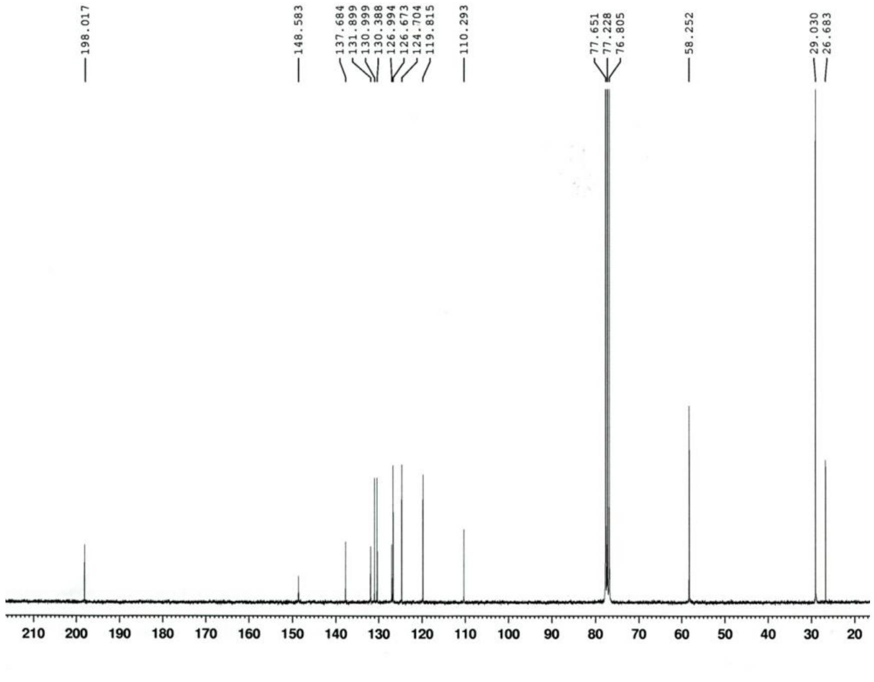

^{1}H NMR (DMSO-d_6, 500 MHz, 300 K) of Compound **5**

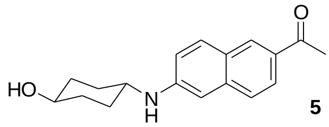

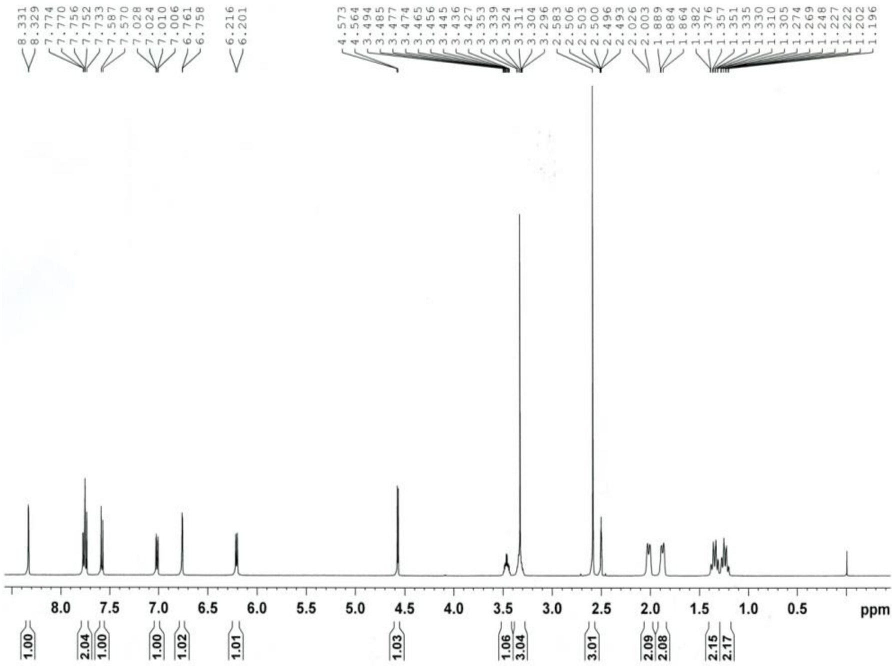

^{13}C NMR (DMSO-d_6, 125 MHz, 300 K) of Compound **5**

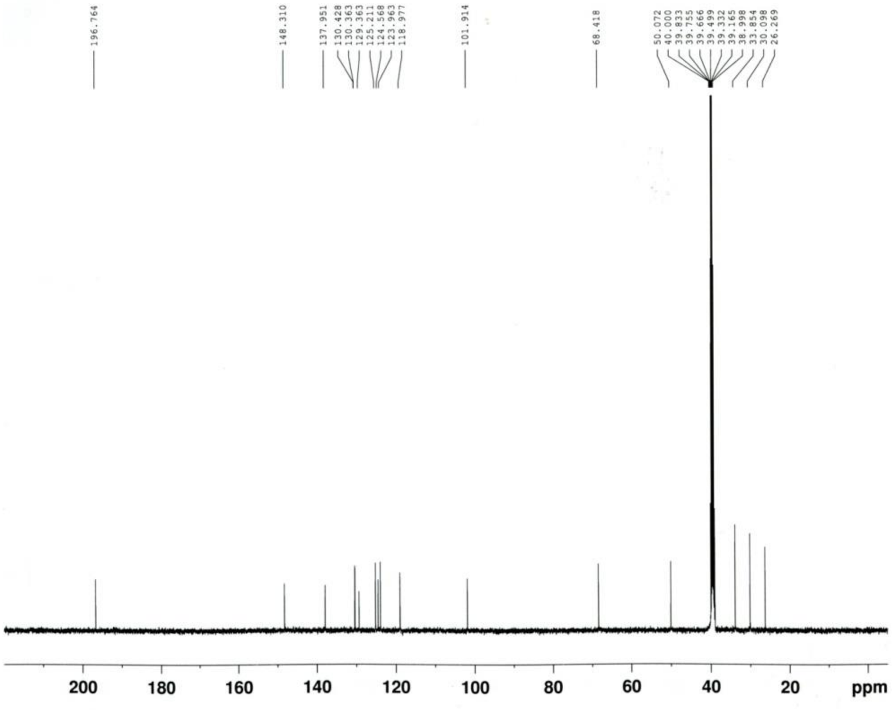

^{1}H NMR (CD_3OD, 300 MHz, 298 K) of Compound **6b**

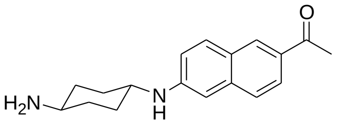

6b

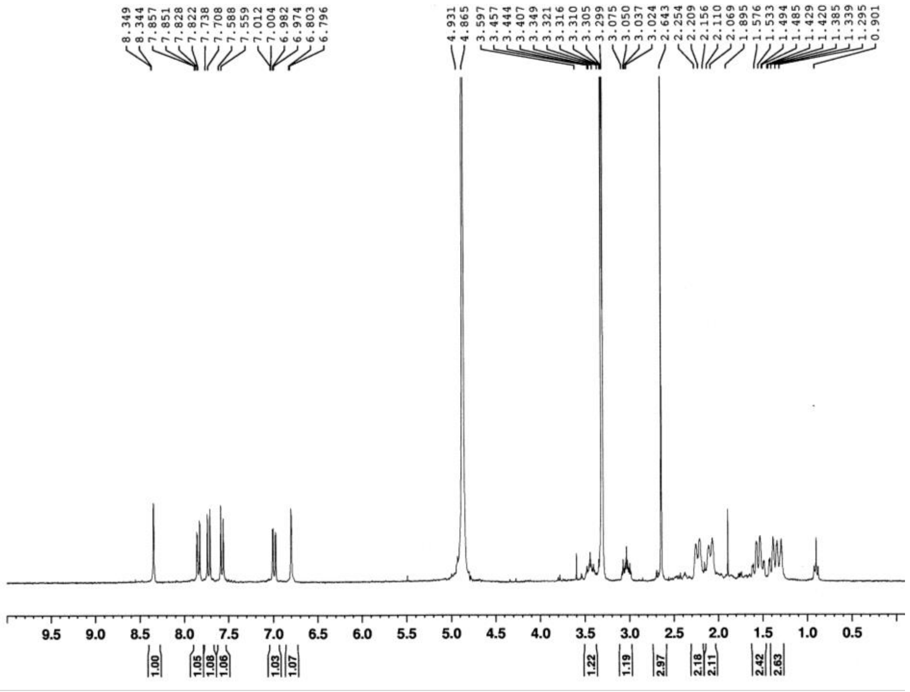

^{13}C NMR (CD_3OD, 75 MHz, 298 K) of Compound **6b**

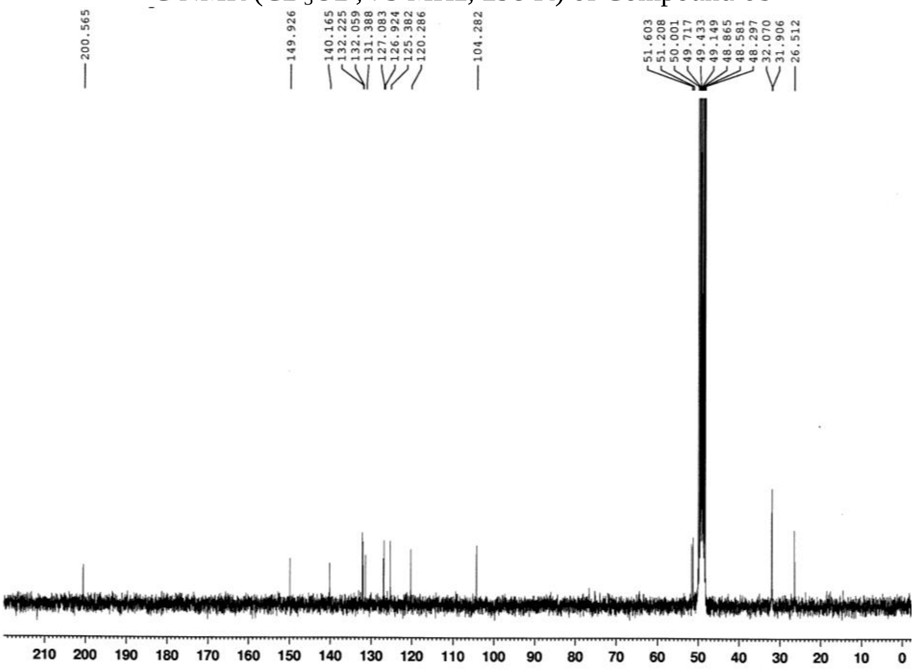

^{1}H NMR ($CDCl_3$, 300 MHz, 298 K) of Compound **6**

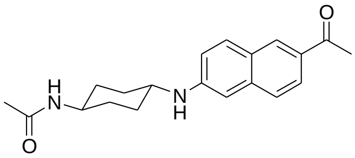

6

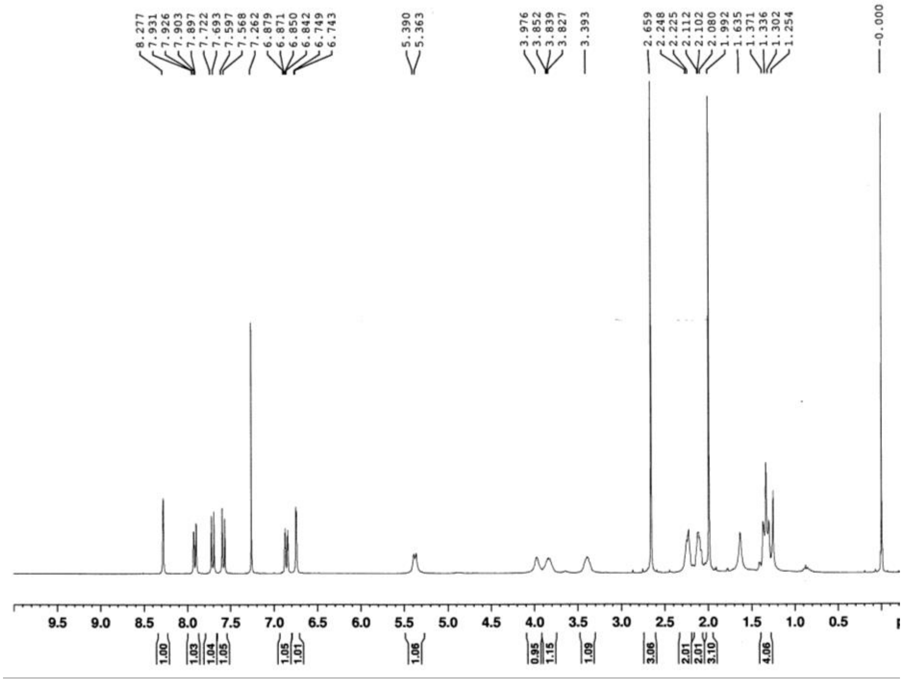

^{13}C NMR ($CDCl_3$, 75 MHz, 298 K) of Compound **6**

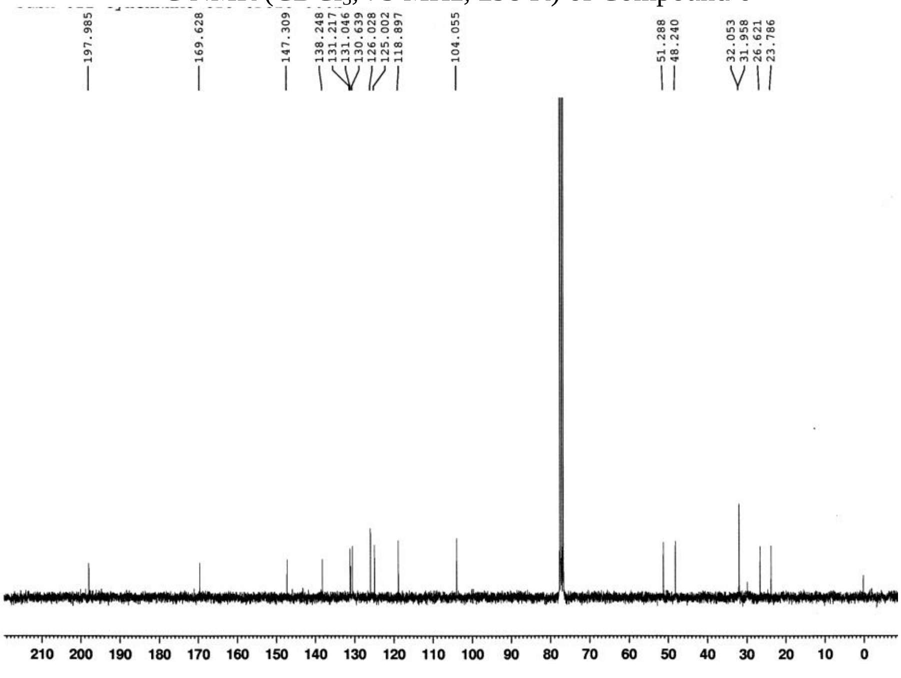

^{1}H NMR ($CDCl_3$, 300 MHz, 298 K) of Compound **8a**

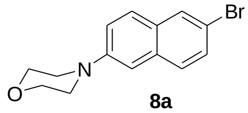

8a

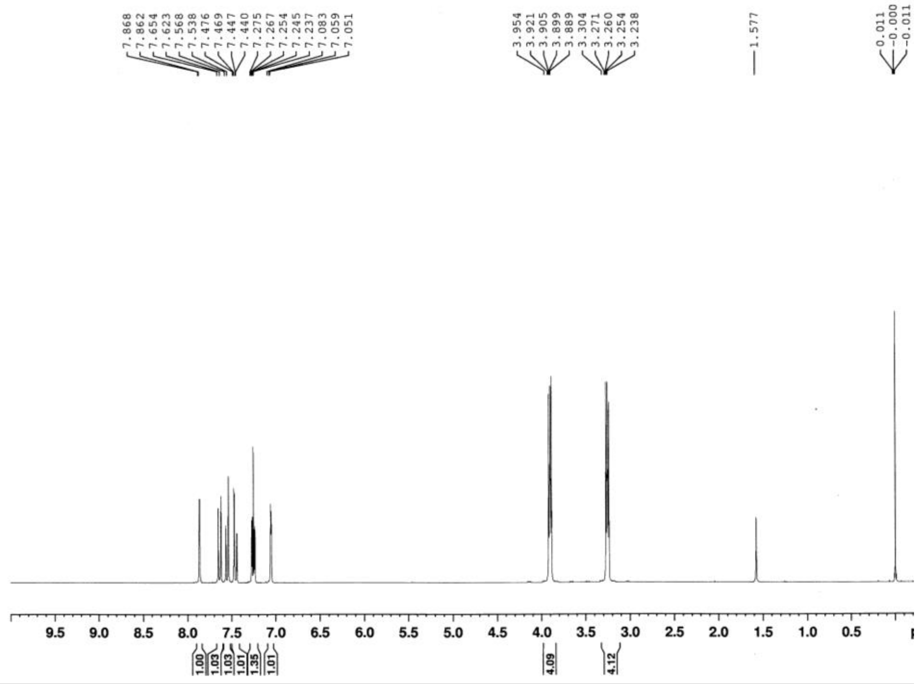

^{13}C NMR ($CDCl_3$, 75 MHz, 298 K) of Compound **8a**

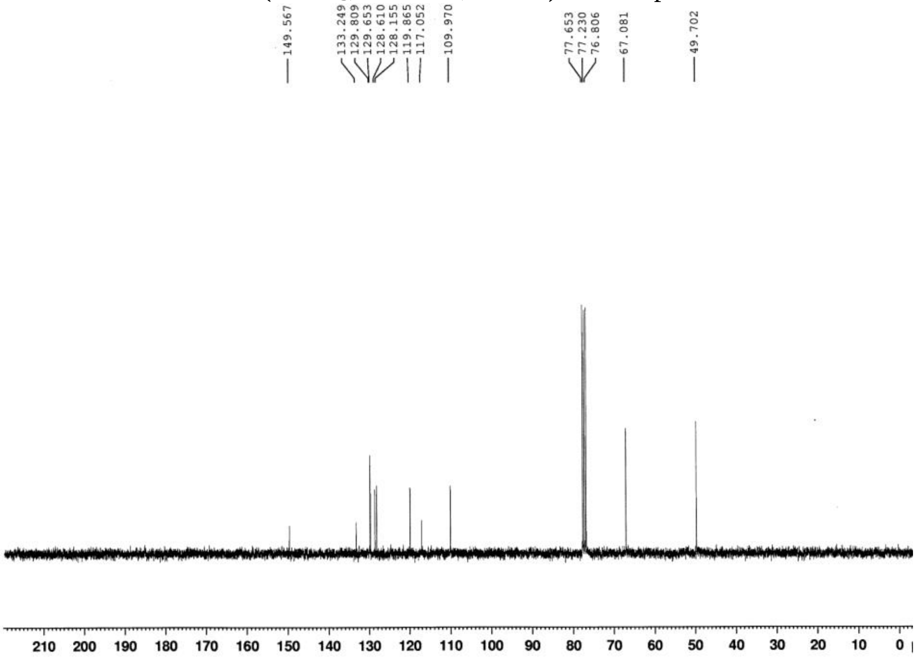

^{1}H NMR ($CDCl_3$, 300 MHz, 298 K) of Compound **8**

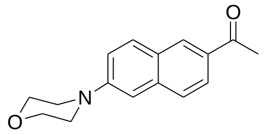

8

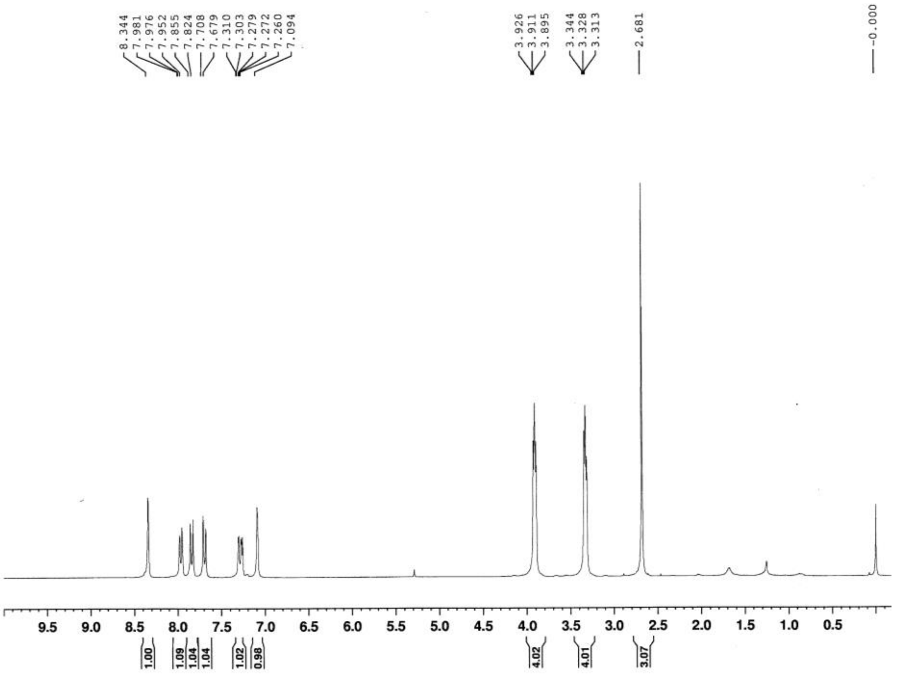

^{13}C NMR ($CDCl_3$, 75 MHz, 298 K) of Compound **8**

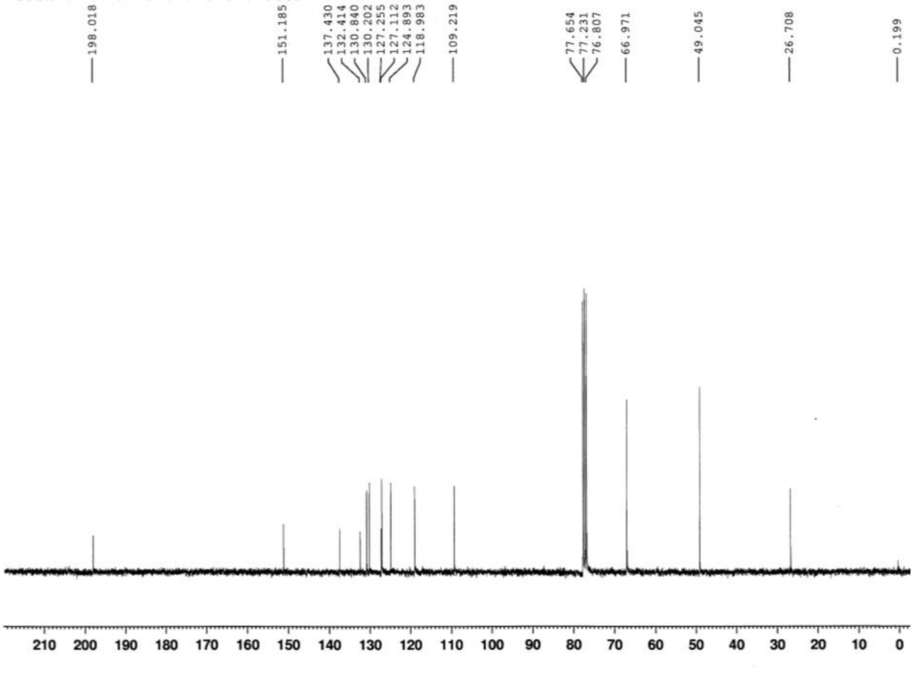

^{1}H NMR ($CDCl_3$, 300 MHz, 298 K) of Compound **9**

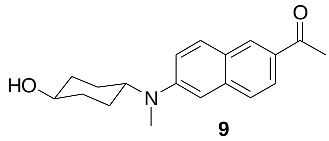

9

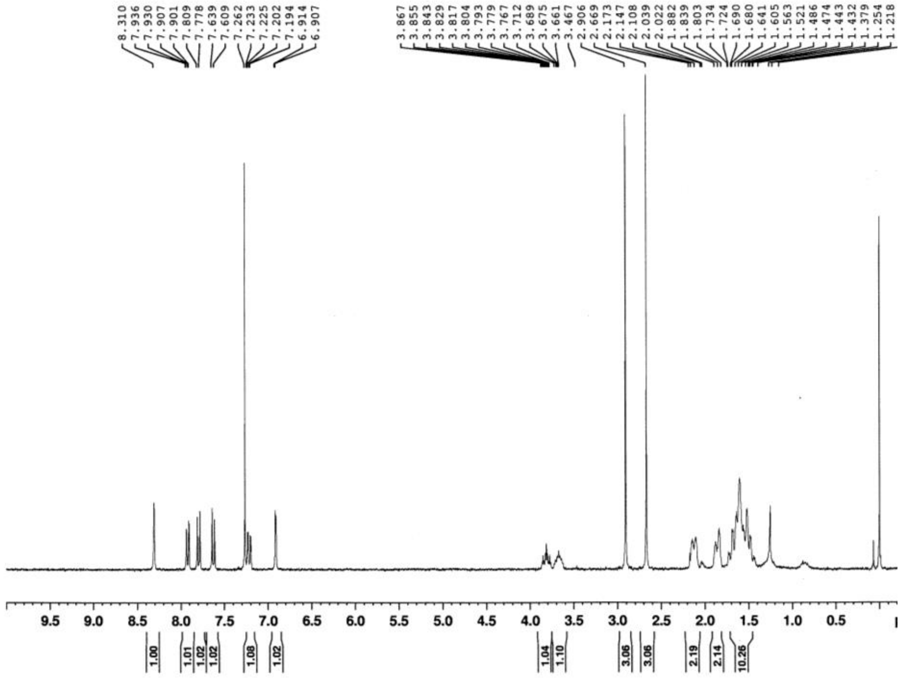

^{13}C NMR ($CDCl_3$, 75 MHz, 298 K) of Compound **9**

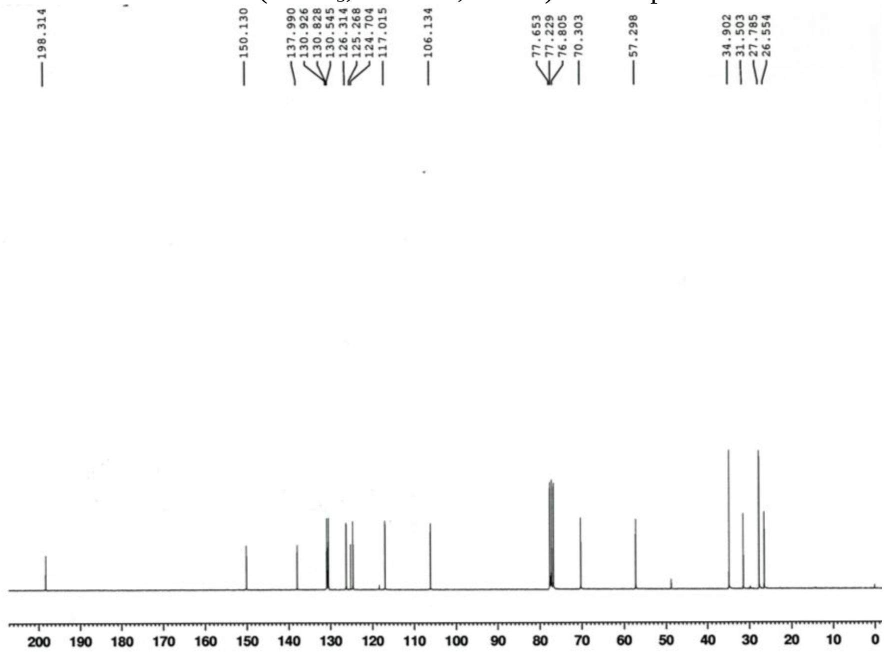

^{1}H NMR (DMSO-d_6, 300 MHz, 298 K) of Compound **10c**

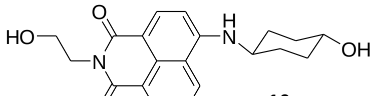

10c

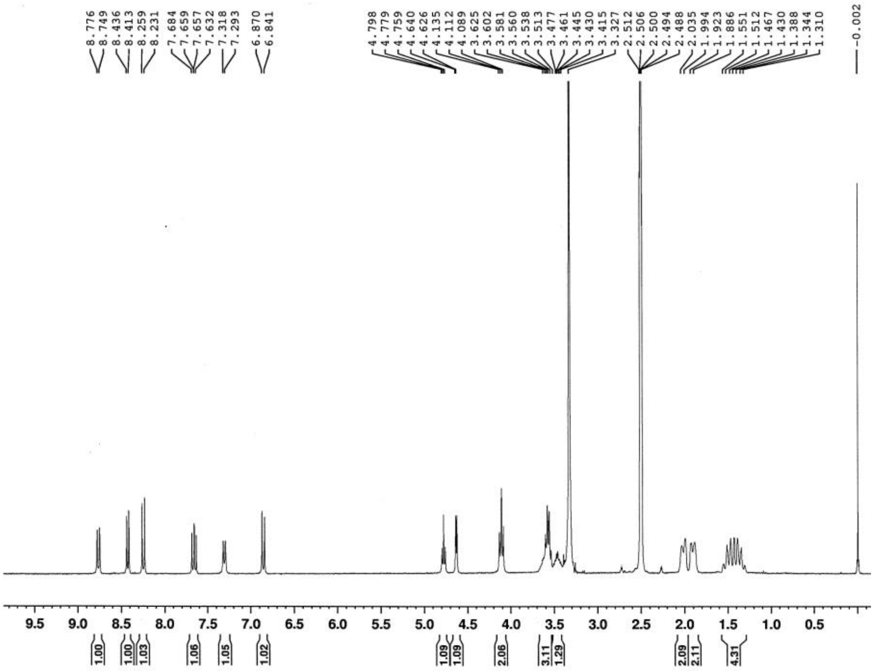

^{13}C NMR (DMSO-d_6, 125 MHz, 298 K) of Compound **10c**

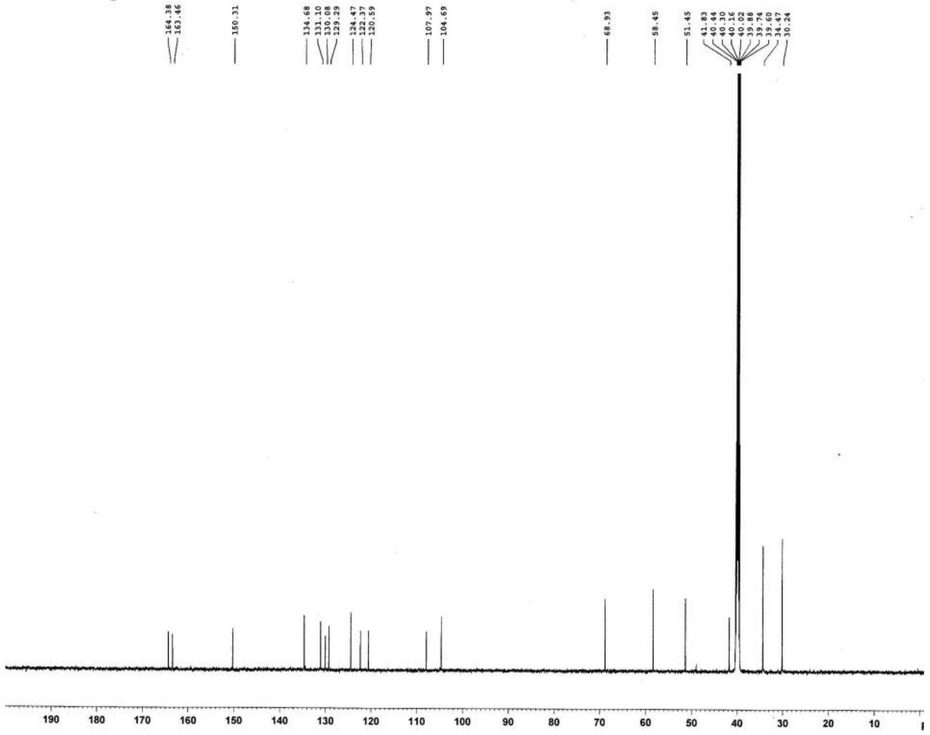

^{1}H NMR (CD_3OD, 300 MHz, 298 K) of Compound **11c**

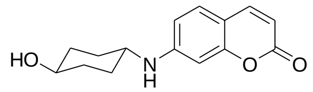

11c

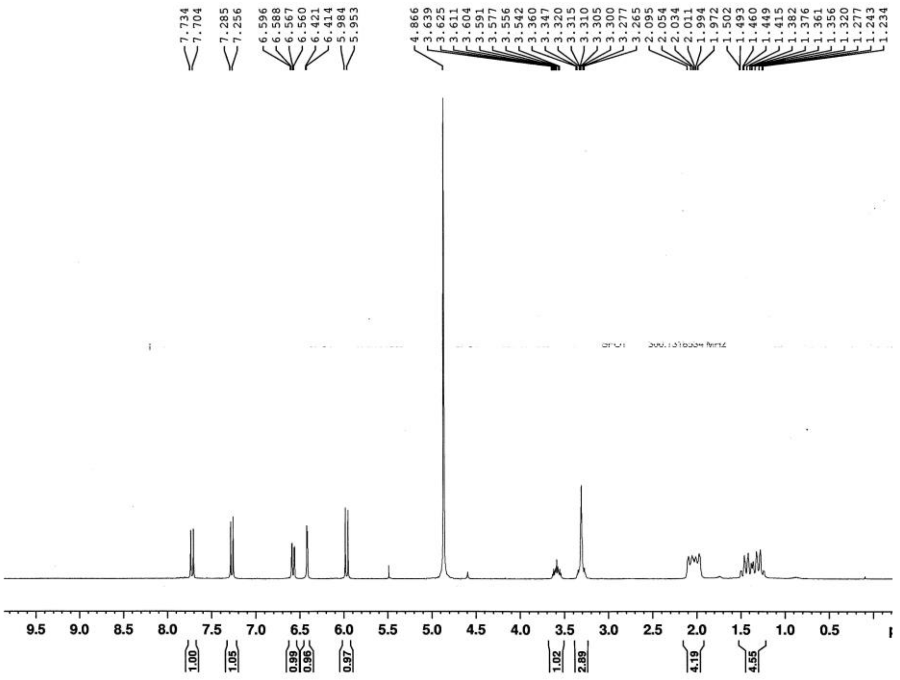

^{13}C NMR (CD_3OD, 75 MHz, 298 K) of Compound **11c**

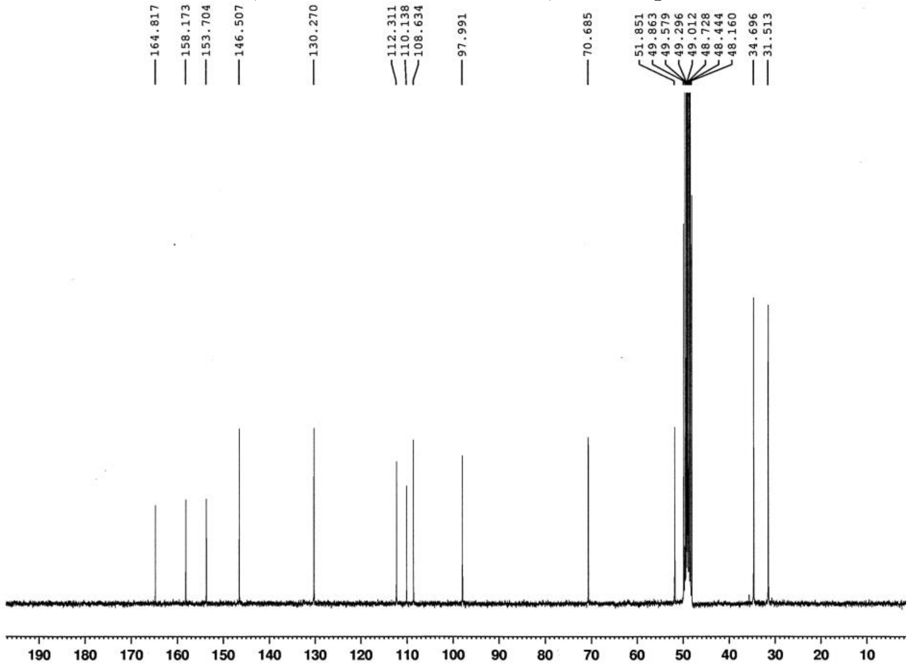

^{1}H NMR (DMSO-d_6, 300 MHz, 298 K) of Compound **12c**

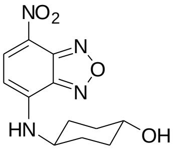

12c

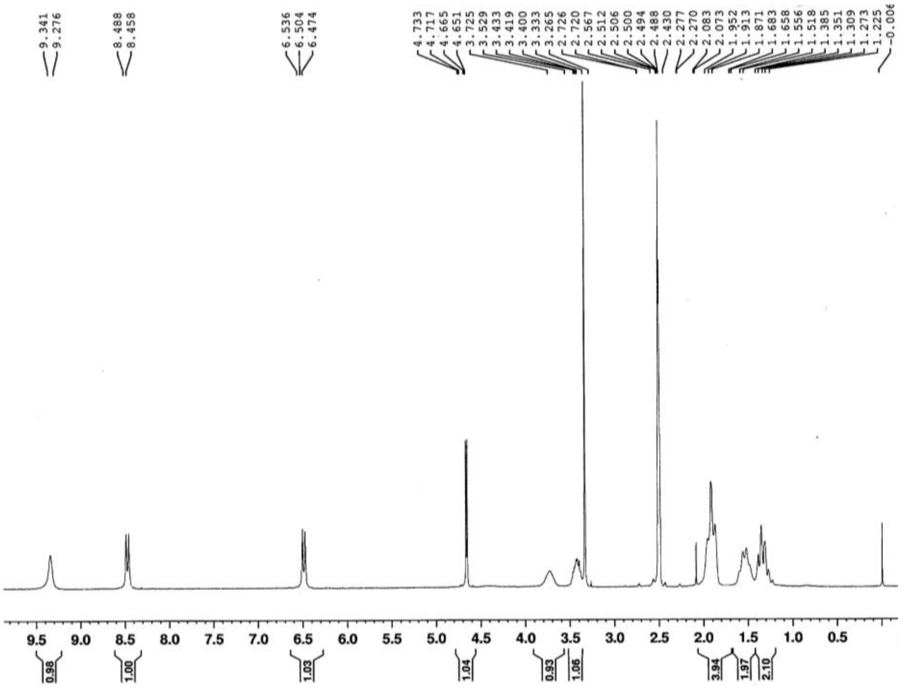

^{13}C NMR (DMSO-d_6, 125 MHz, 298 K) of Compound **12c**

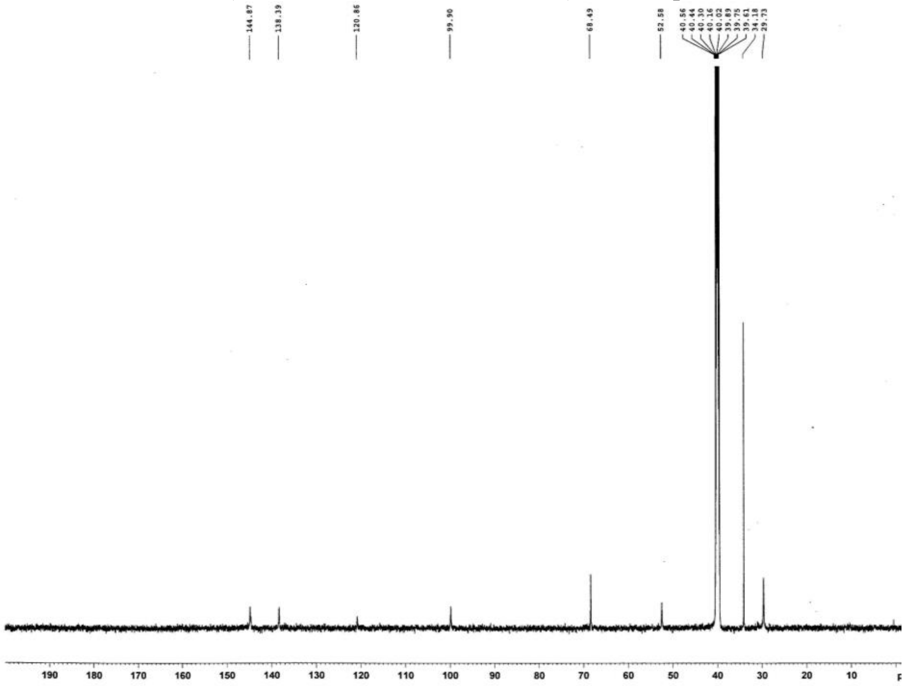

^{1}H NMR ($CDCl_3$, 300 MHz, 298 K) of Compound **13**

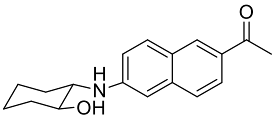

13

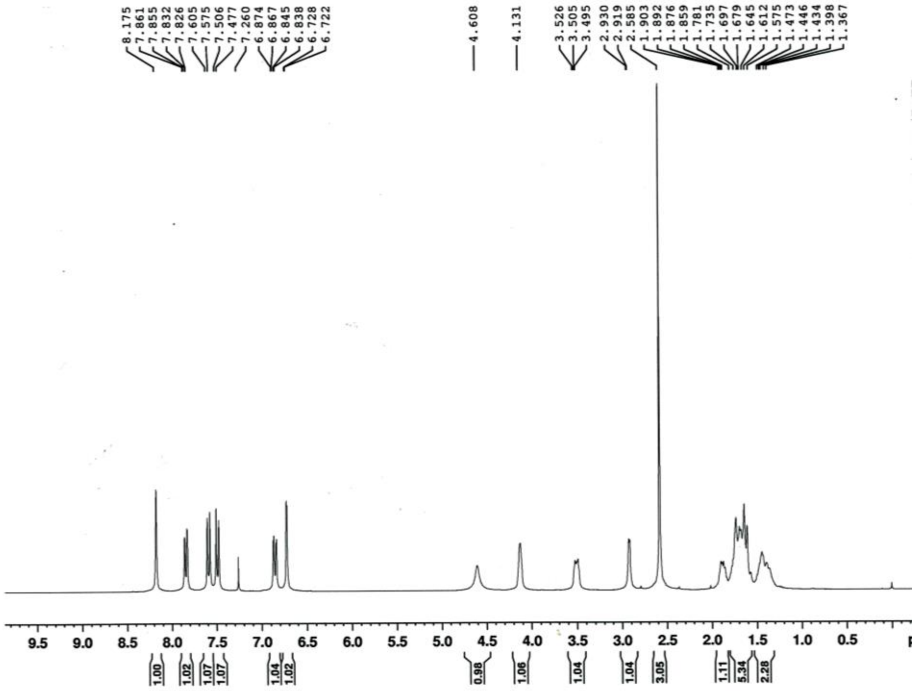

^{13}C NMR ($CDCl_3$, 75 MHz, 298 K) of Compound **13**

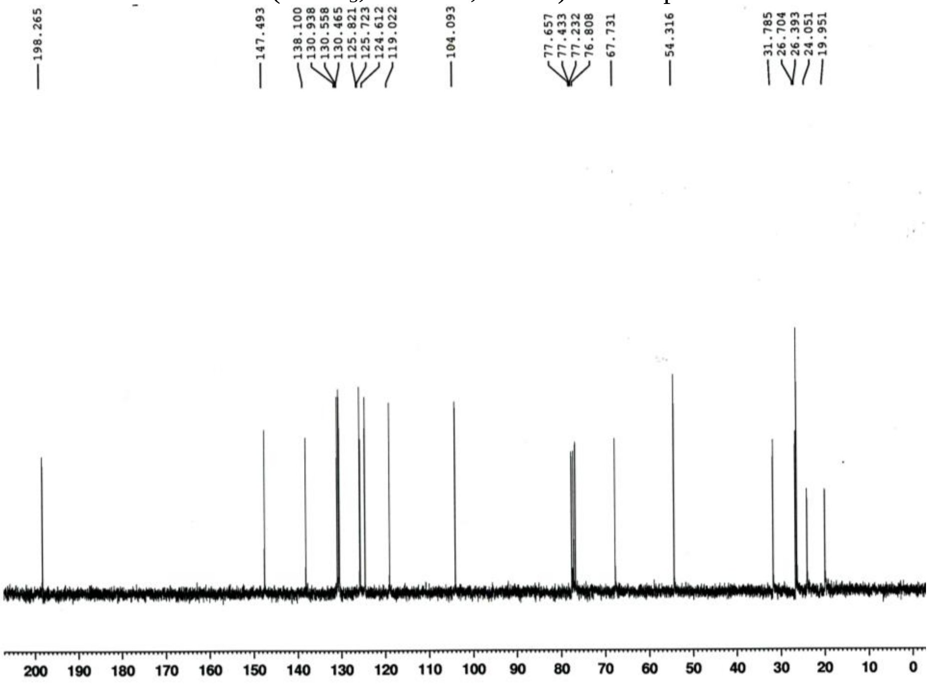

^{1}H NMR ($CDCl_3$, 300 MHz, 298 K) of Compound **14**

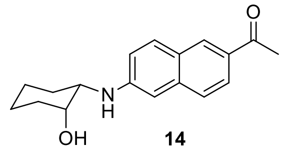

14

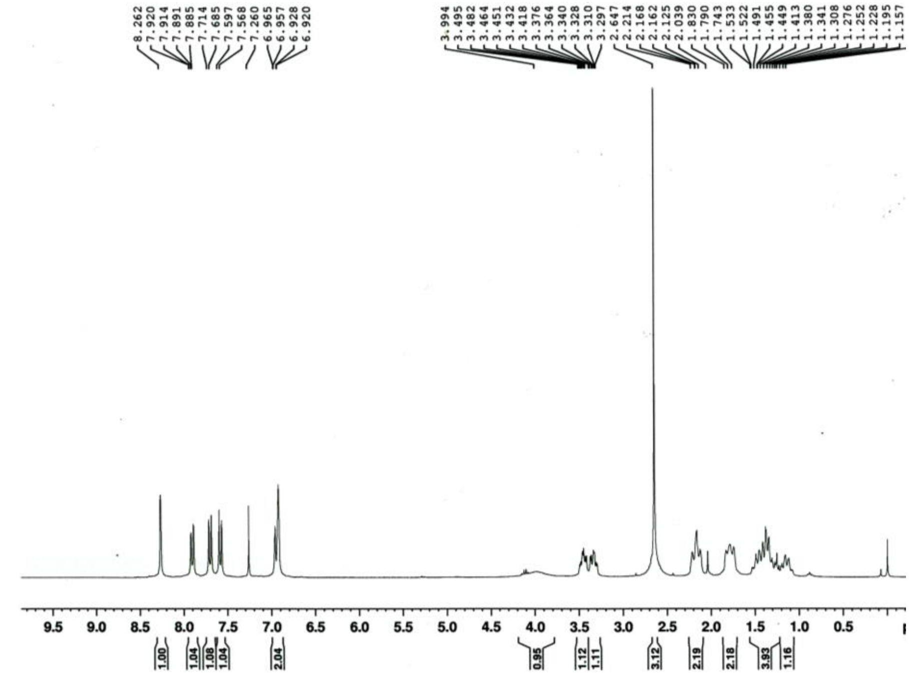

^{13}C NMR ($CDCl_3$, 75 MHz, 298 K) of Compound **14**

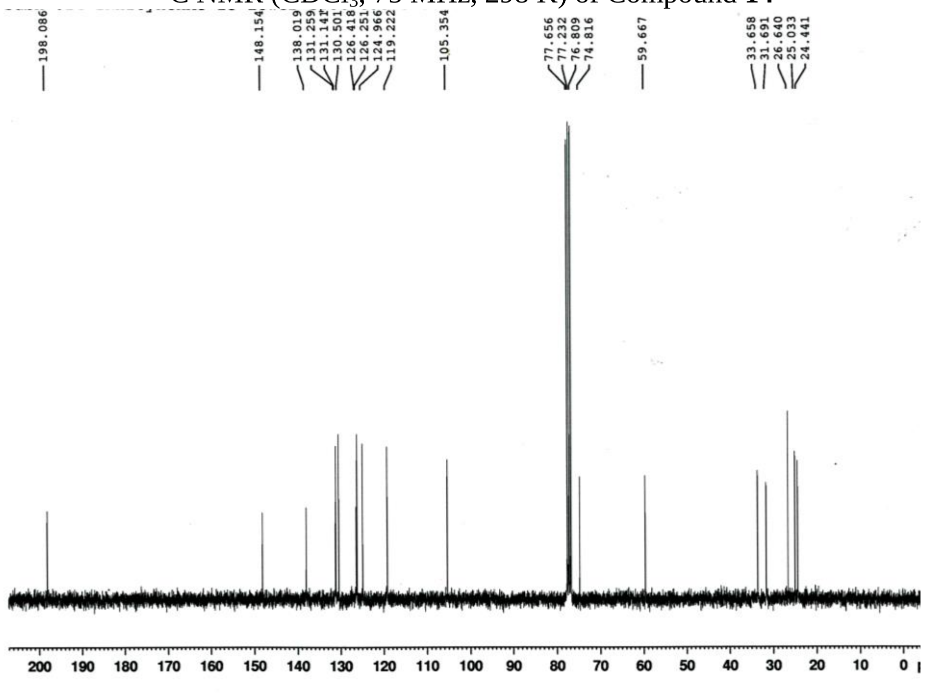

^{1}H NMR ($CDCl_3$, 300 MHz, 298 K) of Compound **15**

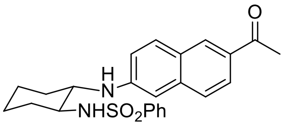

15

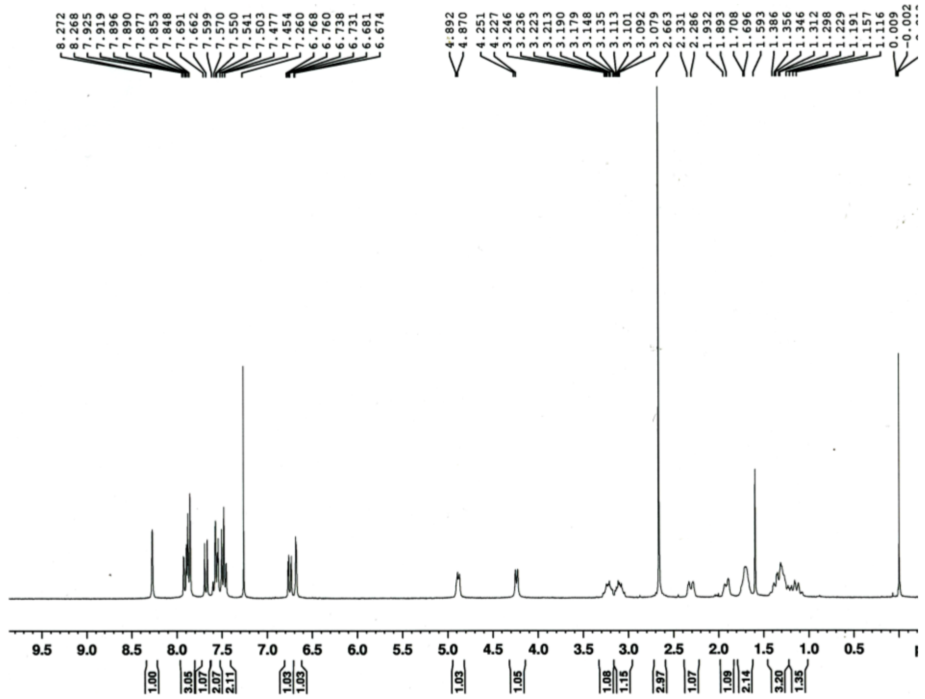

^{13}C NMR ($CDCl_3$, 75 MHz, 298 K) of Compound **15**

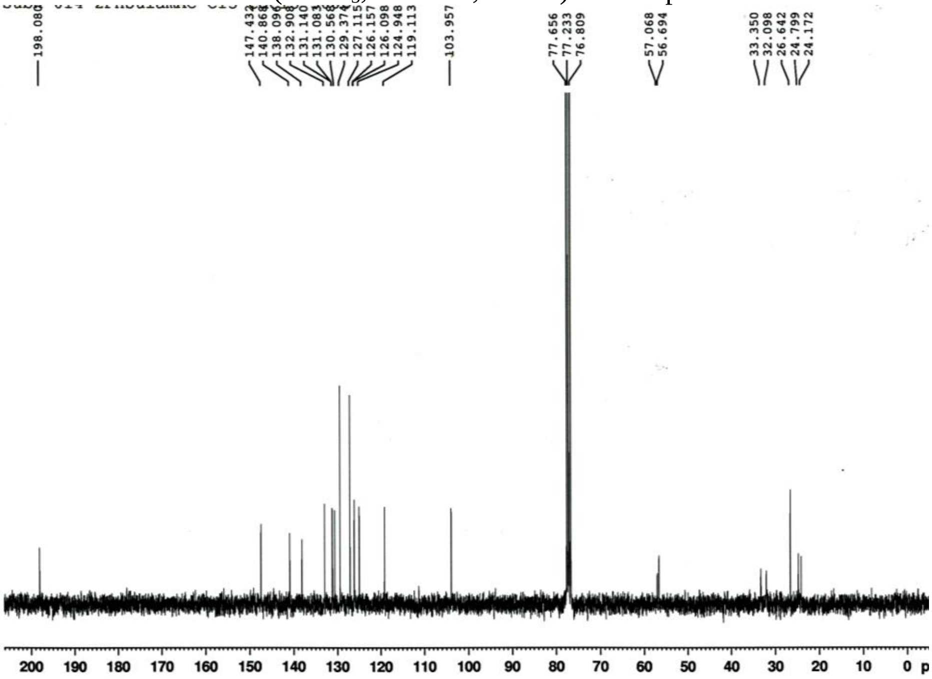

^{1}H NMR ($CDCl_3$, 300 MHz, 298 K) of Compound **16**

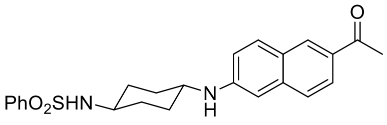

16

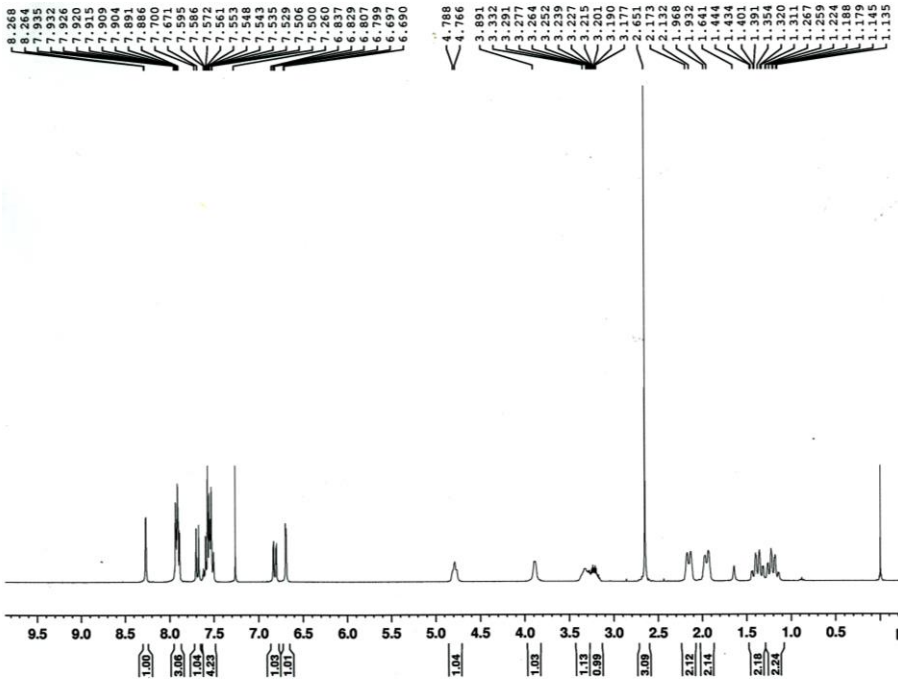

^{13}C NMR ($CDCl_3$, 75 MHz, 298 K) of Compound **16**

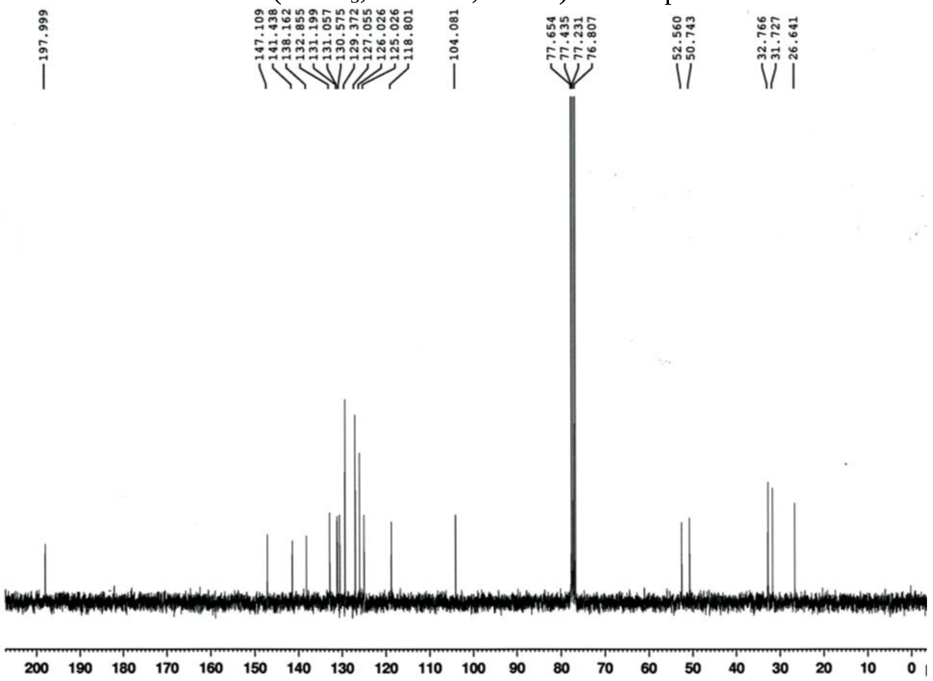

^{1}H NMR ($CDCl_3$, 300 MHz, 298 K) of Compound **17**

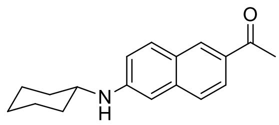

17

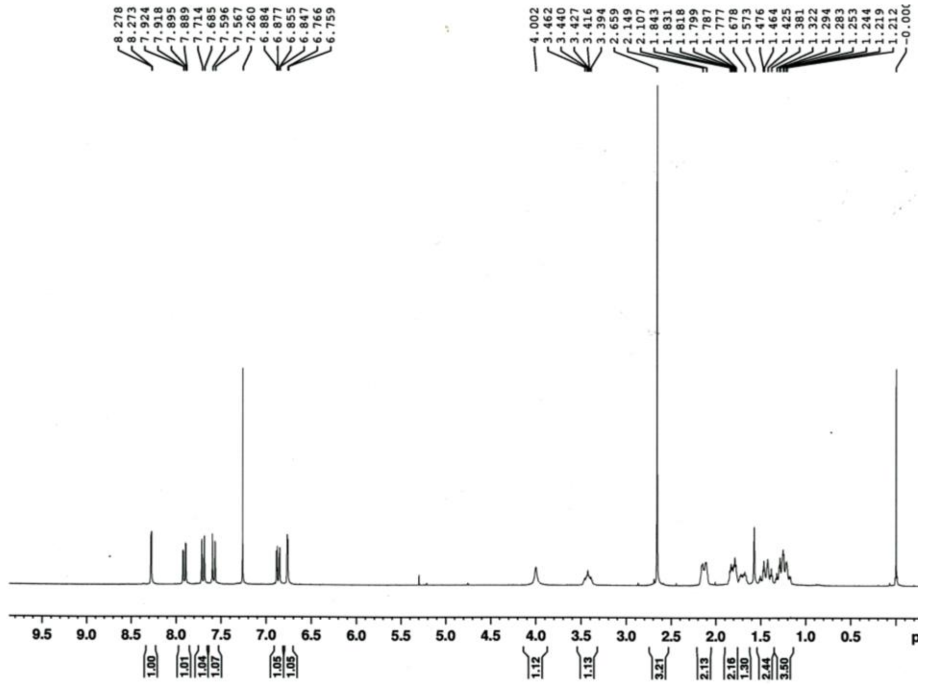

^{13}C NMR ($CDCl_3$, 75 MHz, 298 K) of Compound **17**

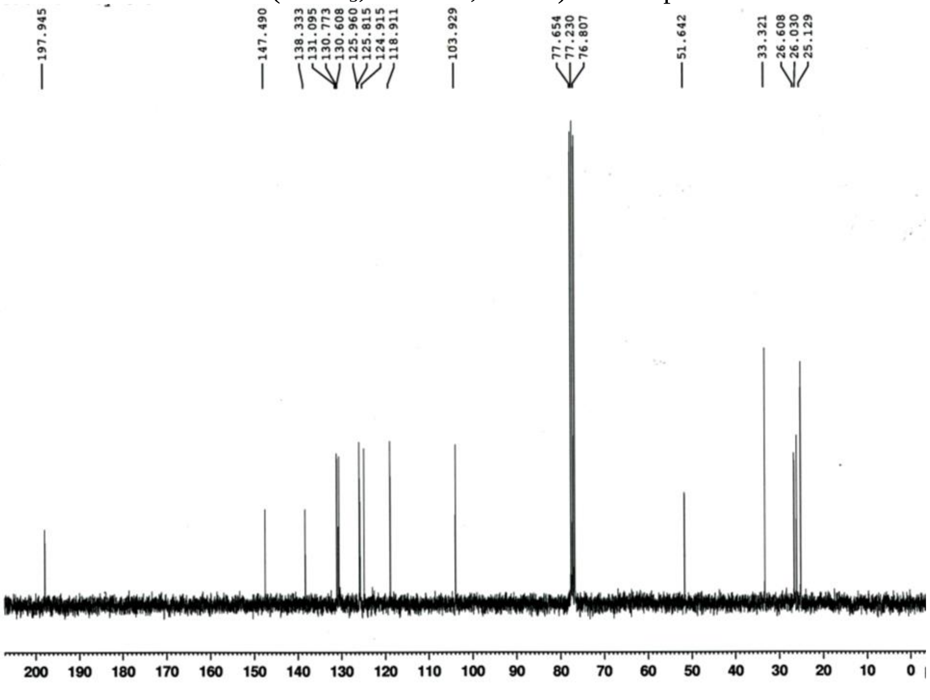

^{1}H NMR ($CDCl_3$, 300 MHz, 298 K) of Compound **18**

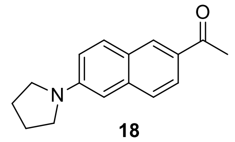

18

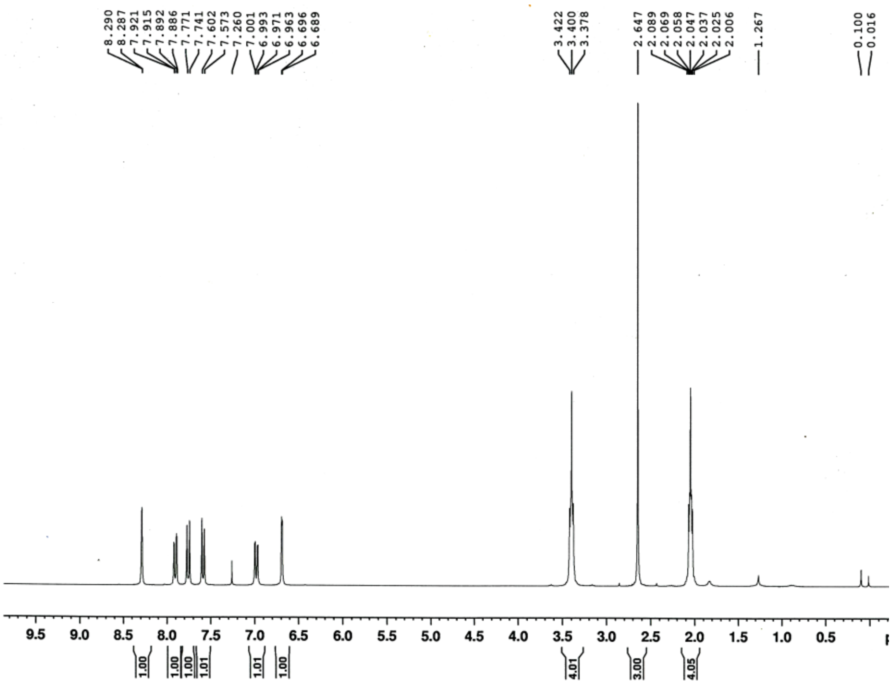

^{13}C NMR ($CDCl_3$, 75 MHz, 298 K) of Compound **18**

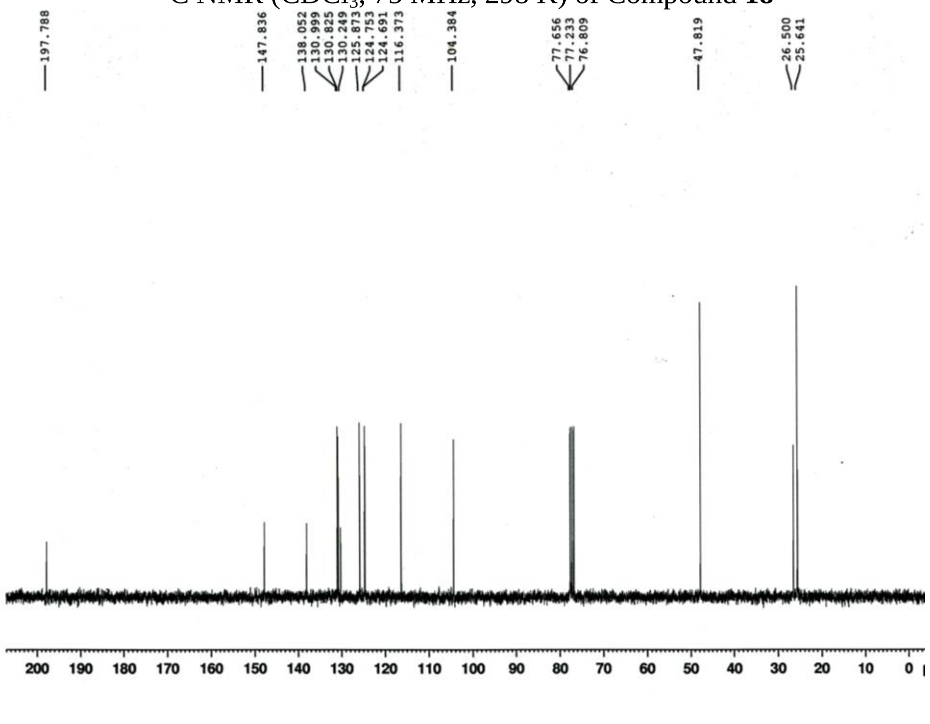

^{1}H NMR ($CDCl_3$, 300 MHz, 298 K) of Compound **21**

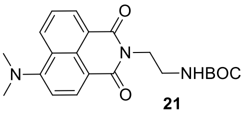

21

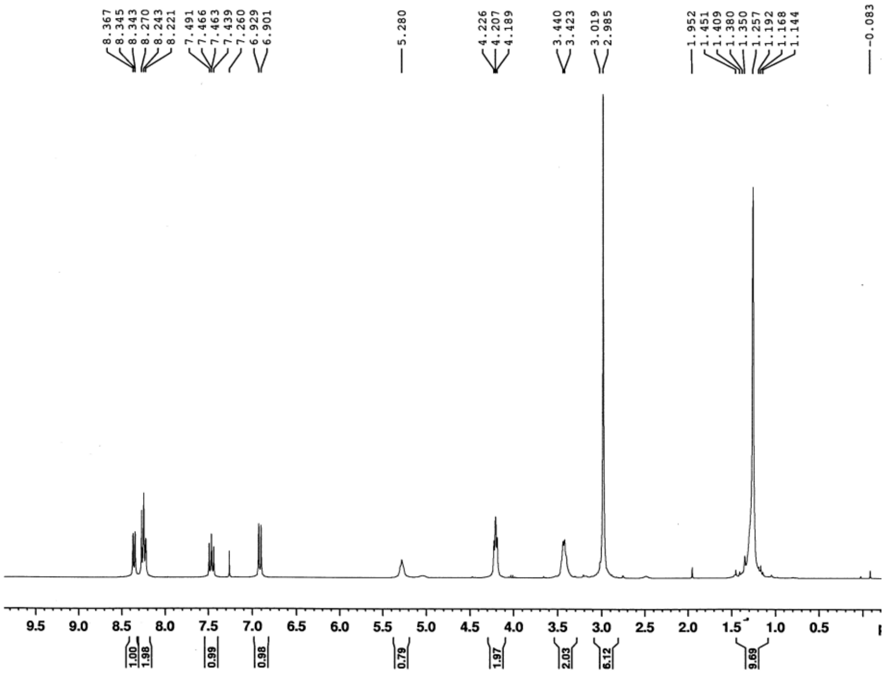

^{1}H NMR ($CDCl_3$, 300 MHz, 298 K) of Compound **23**

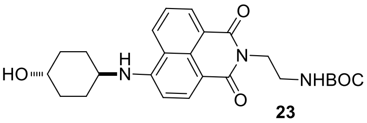

23

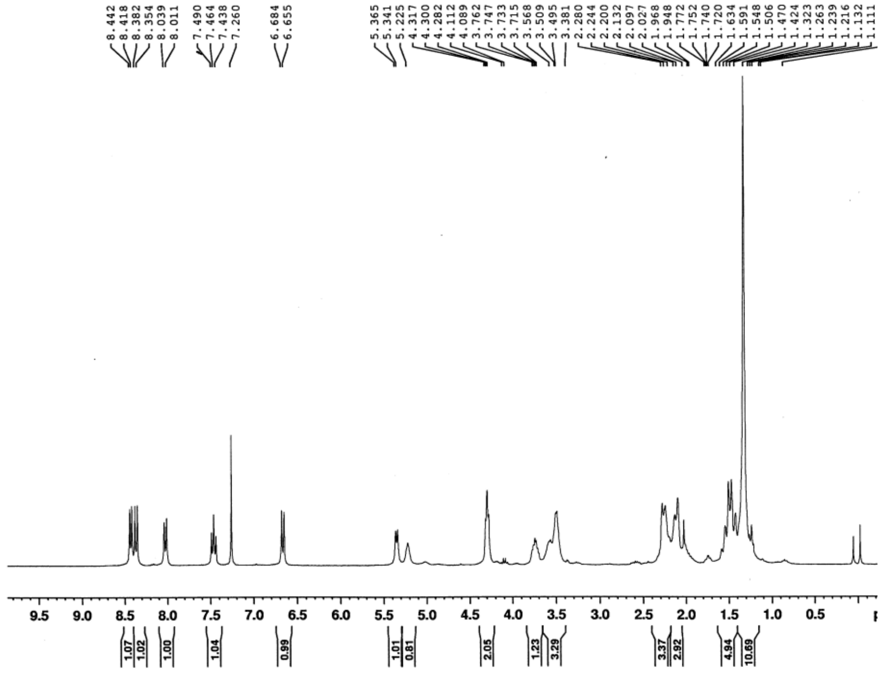

^{1}H NMR ($CDCl_3$, 300 MHz, 298 K) of **P1**

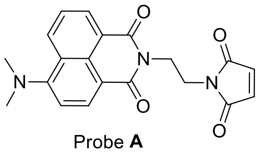

Probe **A**

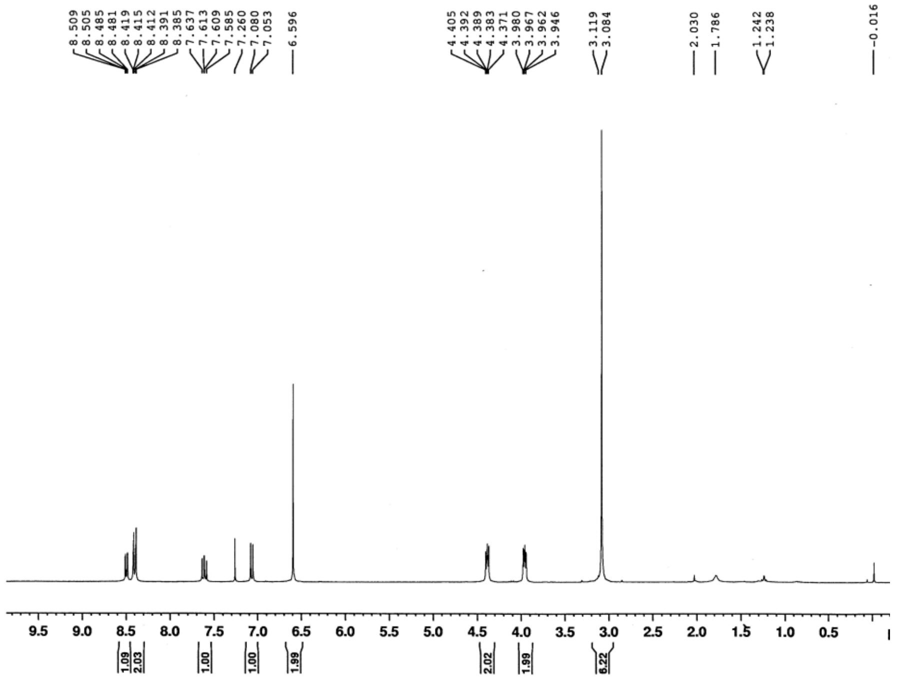

^{13}C NMR ($CDCl_3$, 75 MHz, 298 K) of **P1**

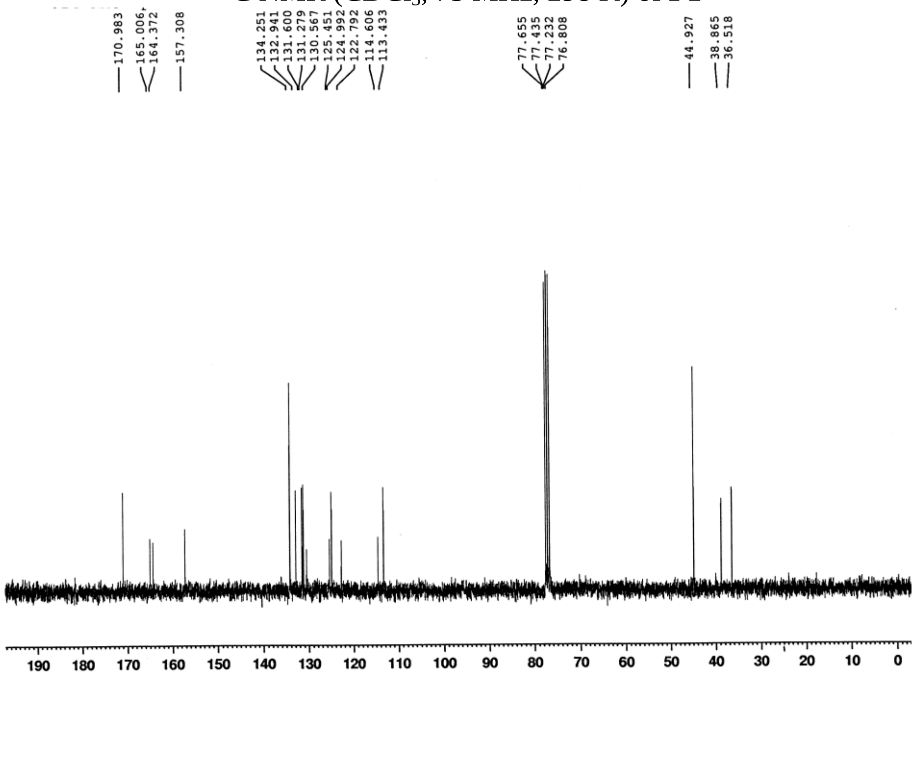

^{1}H NMR ($CDCl_3$, 300 MHz, 298 K) of **P2**

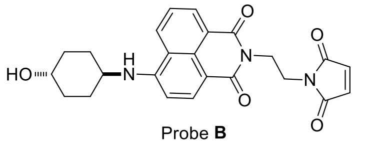

Probe **B**

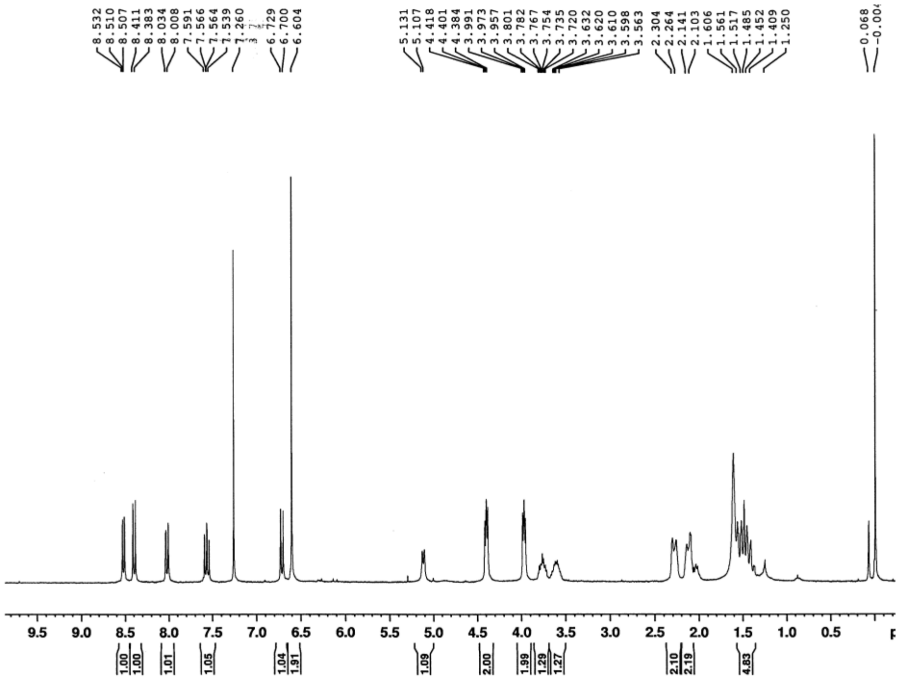

^{13}C NMR ($CDCl_3$, 75 MHz, 298 K) of **P2**

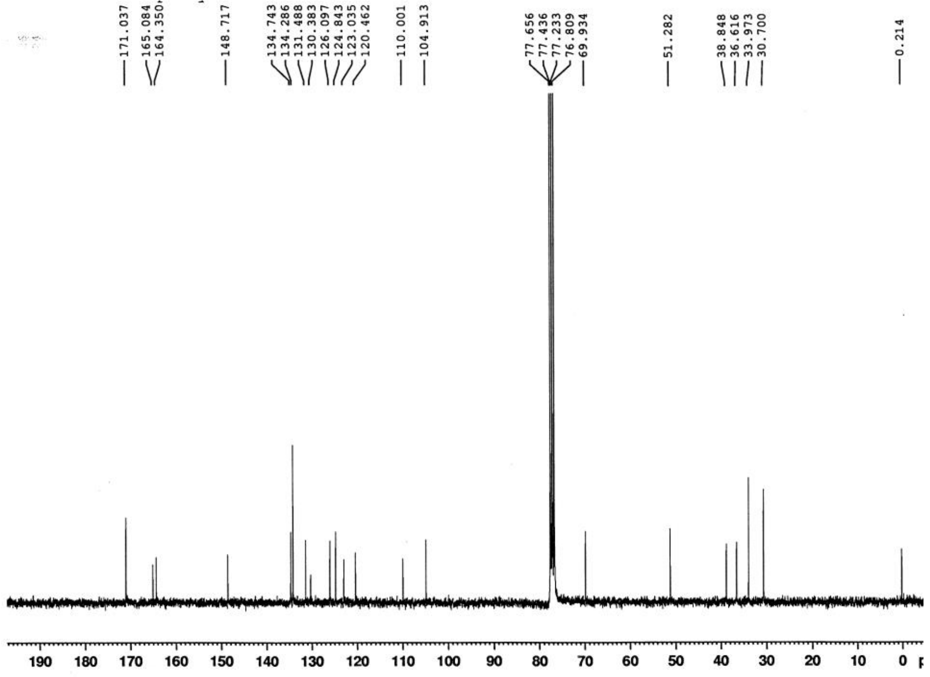
